# What Matters for C_4_ Transporters: Evolutionary Changes of Phospho*enol*pyruvate Transporter for C_4_ Photosynthesis

**DOI:** 10.3389/fpls.2020.00935

**Published:** 2020-06-30

**Authors:** Ming-Ju Amy Lyu, Yaling Wang, Jianjun Jiang, Xinyu Liu, Genyun Chen, Xin-Guang Zhu

**Affiliations:** ^1^ National Key Laboratory of Plant Molecular Genetics, CAS Center for Excellence In Molecular Plant Sciences, Institute of Plant Physiology and Ecology, Chinese Academy of Sciences, Shanghai, China; ^2^ Wisconsin Institute for Discovery & Laboratory of Genetics, University of Wisconsin, Madison, WI, United States

**Keywords:** C_4_ photosynthesis, evolution, *Flaveria*, phospho*enol*pyruvate transporter

## Abstract

C_4_ photosynthesis is a complex trait that evolved from its ancestral C_3_ photosynthesis by recruiting pre-existing genes. These co-opted genes were changed in many aspects compared to their counterparts in C_3_ species. Most of the evolutionary changes of the C_4_ shuttle enzymes are well characterized, however, evolutionary changes for the recruited metabolite transporters are less studied. Here we analyzed the evolutionary changes of the shuttle enzyme phospho*enol*pyruvate (PEP) transporter (PPT) during its recruitment from C_3_ to C_4_ photosynthesis. Our analysis showed that among the two PPT paralogs PPT1 and PPT2, PPT1 was the copy recruited for C_4_ photosynthesis in multiple C_4_ lineages. During C_4_ evolution, PPT1 gained increased transcript abundance, shifted its expression from predominantly in root to in leaf and from bundle sheath cell to mesophyll cell, and gained more rapid and long-lasting responsiveness to light. Modifications occurred in both regulatory and coding regions in C_4_ PPT1 as compared to C_3_ PPT1, however, the PEP transporting function of PPT1 remained. We found that PPT1 of a *Flaveria* C_4_ species recruited a MEM1 B submodule in the promoter region, which might be related to the increased transcript abundance of PPT1 in C_4_ mesophyll cells. The case study of PPT further suggested that high transcript abundance in a proper location is of high priority for PPT to support C_4_ function.

## Highlights

During the evolution of C_4_ photosynthesis, one of the paralogs of PPTs, i.e., PPT1, which shows lower transcript abundance in leaf but higher transcript abundance in root was recruited in multiple C_4_ lineages. Compared to its counterpart in C_3_ species, PPT1 in C_4_ species shows altered expression location, enhanced transcript abundance, increased light responsiveness, which might be related to a newly recruited MEM1 B submodule in its promoter.

## Introduction

Compared to C_3_ photosynthesis, C_4_ photosynthesis has higher light, nitrogen, and water using efficiencies (Sage and Zhu, 2011). It achieves these superior properties through a CO_2_ concentrating mechanism operating in a specialized leaf anatomical feature termed “Kranz anatomy” (Hatch, 1987). The CO_2_ concentrating mechanism involves many enzymes and metabolite transporters, which together pump CO_2_ from mesophyll cells (MC) to bundle sheath cells (BSC), creating a localized high CO_2_ environment in the BSC around ribulose bisphosphate carboxylase/oxygenase (Rubisco). All genes required for the operation of C_4_ photosynthesis are pre-existing in C_3_ ancestors and play housekeeping functions (Aubry et al., 2011). The evolution of C_4_ photosynthesis is therefore a process of recruiting and re-organizing pre-existing genes to fulfill new functions in C_4_ photosynthesis (West-Eberhard et al., 2011; Burgess et al., 2016).

The specific modifications that have occurred on the genes recruited for C_4_ photosynthesis are unknown. Comparisons of genes involved in C_4_ photosynthesis in C_4_ species and their counterparts in C_3_ species showed that these genes were modified in different aspects (Gowik and Westhoff, 2011), e.g., increasing transcript abundance [see review in (Hibberd and Covshoff, 2010)]; acquiring cell specific expression (Hatch and Osmond, 1976; Aubry et al., 2014); gaining modifications in protein coding regions resulting in suitability for C_4_ photosynthesis (Blasing et al., 2000; Paulus et al., 2013); obtaining new *cis*-elements (Gowik et al., 2004; Williams et al., 2016; Gupta et al., 2020); and having more copies (Bianconi et al., 2018).

Most current evolutionary studies on genes involved in C_4_ photosynthesis focus on enzymes directly related to the carbon shuttle enzymes, such as phospho*enol*pyvuvate (PEP) carboxylase (PEPC), phospho*enol*pyvuvate carboxykinase (PEP-CK), nicotinamide adenine dinucleotide phosphate (NADP) malic enzyme (NADP-ME), pyruvate phosphate kinase (PPDK), and malate dehydrogenase (MDH) (Westhoff and Gowik, 2004; Christin and Besnard, 2009; Christin et al., 2013; Moreno-Villena et al., 2018). However, to establish an efficient CO_2_ concentrating mechanism, C_4_ plants recruited these carbon shuttle enzymes and required metabolite transporters, but also recruited a number of proteins responsible for transport of metabolites required for C_4_ photosynthesis in both MC and BSC. In fact, compared to C_3_ photosynthesis, the extensive usage of transporters is a major feature of C_4_ photosynthesis. Quantitatively, to produce one molecule of triose phosphate for the synthesis of sucrose, only one transporter is needed in C_3_ photosynthesis, while at least 30 metabolite transport steps are involved in the NADP-ME type C_4_ photosynthesis (Weber and Von Caemmerer, 2010).

Furthermore, the flux through the transporters is much higher in C_4_ (Wang et al., 2014). In C_3_ plants, the end-product of photosynthesis, *i.e.*, triose phosphate (TP), is exported as one unit, therefore the flux through the triose phosphate transporter is 1/3 of the photosynthetic CO_2_ uptake rate. In C_4_ photosynthesis, however, the flux of metabolite transport between different compartments is higher than the photosynthetic CO_2_ uptake rate due to the leakage of CO_2_ from BSC to MC. Furthermore, C_4_ plants usually have a higher leaf photosynthetic CO_2_ uptake rate compared to C_3_ plants. Therefore, C_4_ photosynthesis demands a much higher capacity for metabolite transport (Hatch and Osmond, 1976; Von Caemmerer and Furbank, 2003). Indeed, a number of transporters on the chloroplast envelope, including PEP transporter (PPT), pyruvate transporter (BASS2), and malate transporter in MC (DIT1), all show higher transcript abundance in C_4_ species than in C_3_ species (Emms et al., 2016; Lyu et al., 2018; Moreno-Villena et al., 2018). Identifying the C_4_ paralogs of individual metabolite transporters, understanding their evolutionary modifications and the molecular mechanisms behind the increased abundance or capacity of these transporters can help better understand the emergence of C_4_ metabolism and guide the engineering of C_4_ metabolism into a C_3_ metabolic background.

**Figure 1:**
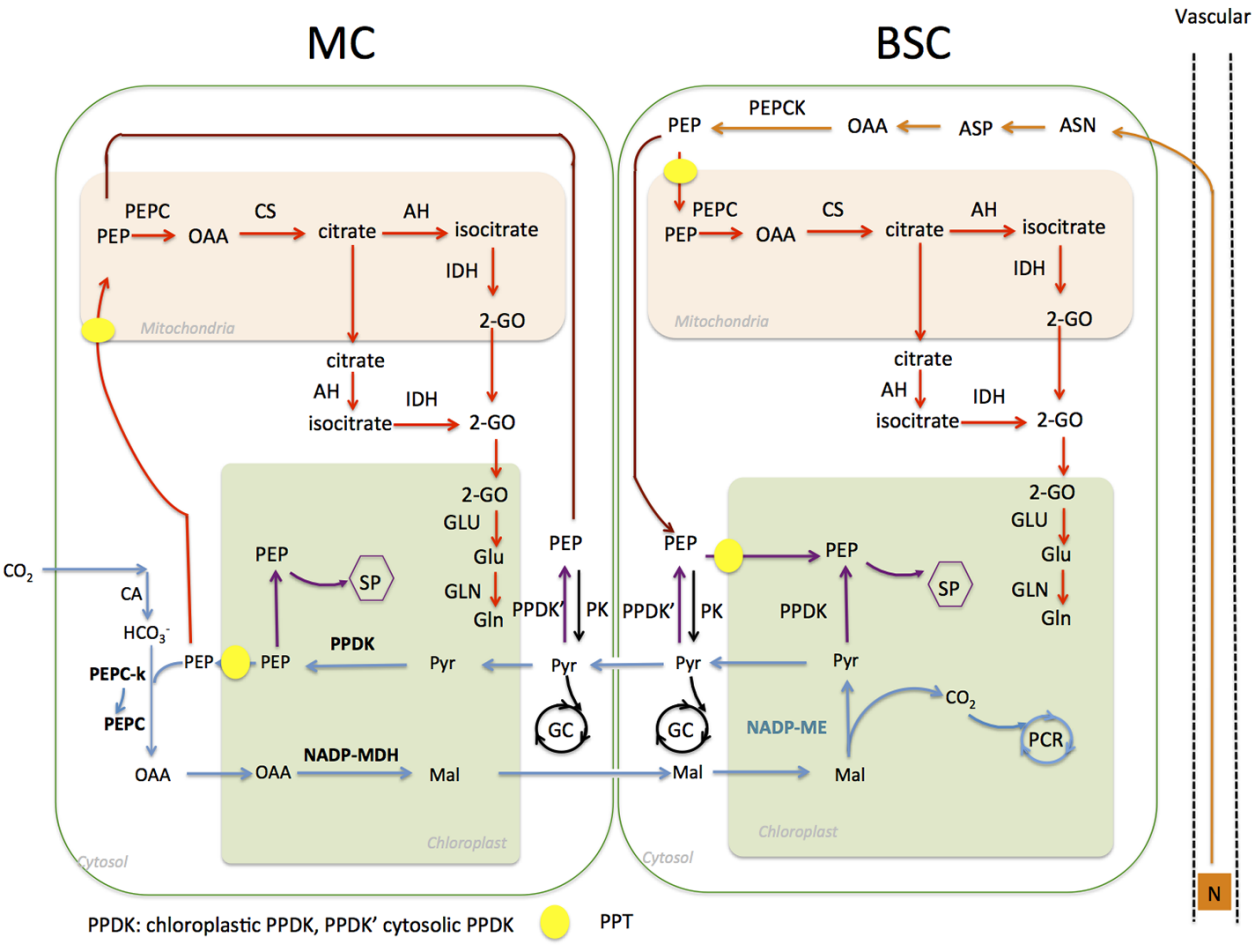
Schematic representation of phospho*enol*pyruvate (PEP) related metabolic pathways in nicotinamide adenine dinucleotide phosphate malic enzyme (NADP-ME) type C_4_ *Flaveria* species In MC, PEP is used as a substrate for PEPC catalyzed carboxylation (blue lines). It is also a substrate of the shikimate pathway (SP) in chloroplasts, which is expected to exist in both MC and BSC (purple lines); moreover, phospho*enol*pyruvate transporter (PEP) is a substrate for the citrate pathway in mitochondria (red lines) and glycolysis in cytosol (black lines). PEP is also involved in nitrogen recycling from xylem (orange lines). (Abbreviations: MC, mesophyll cell; BSC, bundle sheath cell; SP, shikimate pathway; PCR, photosynthetic carbon reduction; GC, glycolysis cycle.)

In this study, we aim to characterize the evolutionary changes of a C_4_ metabolite transporter PPT, which transports PEP (Knappe et al., 2003), a substrate for the first step of C_4_ acid formation in C_4_ photosynthesis. In fact, PEP is involved in a number of metabolic pathways in higher plants. **Figure 1** shows the reactions for which PEP is either a substrate or a product in a typical NADP-ME type C_4_ leaf. Specifically, PEP is the substrate of PEPC, and its carboxylation represents the first step of CO_2_ fixation in C_4_ photosynthesis. PEP is also involved in the shikimate pathway in chloroplasts, which generates aromatic amino acids and secondary metabolites (Fischer et al., 1997; Herrmann and Weaver, 1999). Moreover, PEP is a substrate of the citric acid cycle in mitochondria (Krebs, 1940). Recent studies show that PEP is involved in nitrogen recycling from xylem (Bailey and Leegood, 2016) and in nitrogen mobilization from aging leaves (Taylor et al., 2010).

Considering that PEP functions in multiple metabolic pathways, it is safe to infer that the PEP transporting process is crucial in plants. Here, we conducted a systematic comparison of different properties of PPT between C_3_ and C_4_ plants. Specifically, we first constructed a phylogeny of PPT in Viridiplantae, which includes 23 species spanning chlorophytes to angiosperms to infer the orthologous relationships and copy number of PPT. Then, we compared a number of properties of PPT between C_3_ and C_4_ species, including PPT gene expression, amino acid sequences, and physiological functions. Our results showed that the paralog with relatively low transcript abundance in leaf of C_3_ species was constantly recruited for C_4_ photosynthesis in multiple C_4_ lineages. In an example study in *Flaveria*, we found that PPT1 from a C_4_ species gained a MEM1 B submodule, which might contribute to the changes in transcriptional properties of PPT1 in C_4_ species. Comparing PPT1 between C_4_ and C_3_ species showed that PPT1 has dramatic modifications in the coding region, however, its metabolic function remained the same. The evolutionary changes of PPT suggest that high transcript abundance in the proper location is the key feature of transporters for C_4_ photosynthesis.

## Materials and Methods

### Construction of the Phospho*enol*pyruvate Transporter Phylogenetic Tree

**Figure 2:**
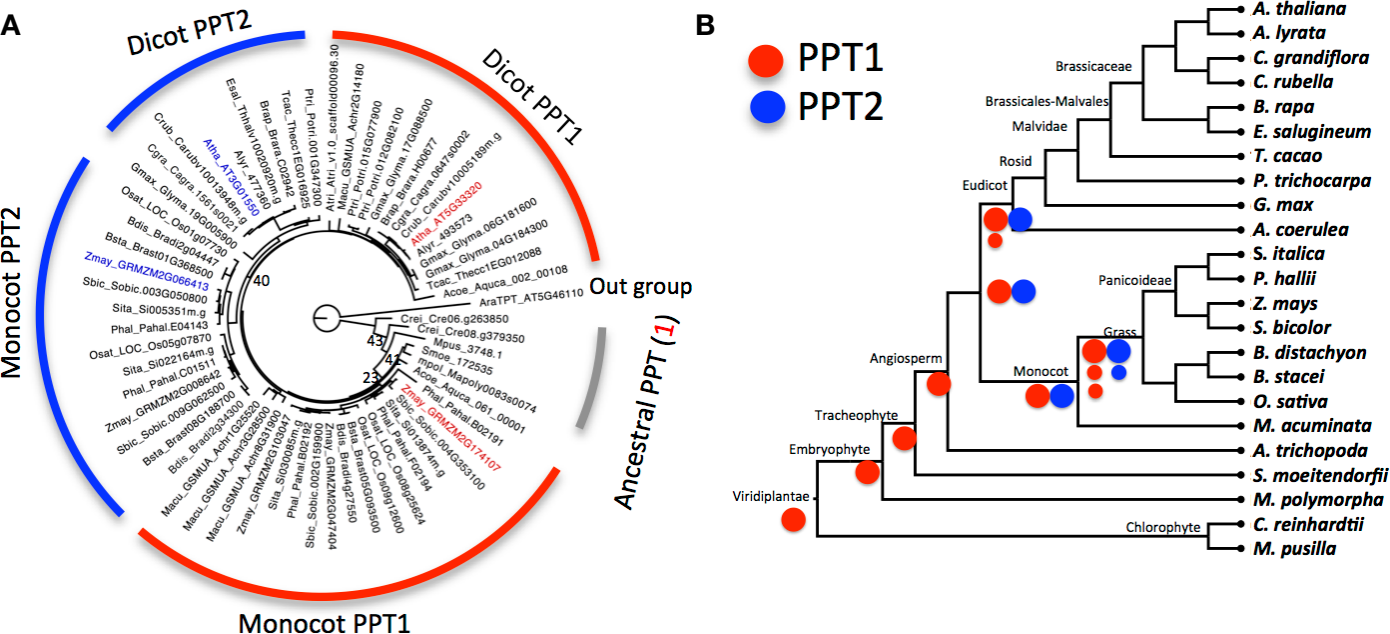
The evolution of phospho*enol*pyruvate transporter (PPT) in Viridiplantae **(A)** Gene tree of PPT family from 23 representative species of Viridiplantae. The tree was inferred from the alignment of protein sequences of PPT using the maximum likelihood method. Numbers beside each node are the bootstrap scores from 1,000 simulated trees; bootstrap scores lower than 60 in the major branch are shown (full bootstrap scores are in **Figure S1**). PPT1 of *Arabidopsis thaliana* and *Zea mays* are highlighted in red and PPT2 in blue. Triose phosphate/phosphate translocator (TPT) of *A. thaliana* is used as outgroup. **(B)** Schematic representation of the evolution of PPT1 and PPT2 based on phylogenetic relationship of species. Ancestral species have one copy of PPT that is more similar to PPT1 than to PPT2 of higher species. PPT1 has one or two copies in eudicot species and two or three copies in monocot species. Red circles represent PPT1 and blue circles represent PPT2, large circles stand for original copies, and small circles for duplicated copies after the division of monocots and dicots. The phylogenetic relationship of species is inferred from the Phytozome website.

To construct the phylogenetic tree of PPT, we used protein sequences from 23 species with genome sequences available in phytozome (http://phytozome.jgi.doe.gov/). These included representative species along the phylogeny of Viridiplantae, spanning from basal species belonging to chlorophytes (*Micromonas pusilla* and *Chlamydomonas reinhardtii*), embryophytes (*Marchantia polymorpha*), tracheophytes (*Selaginella moellendorffii*), and to higher angiosperm plants (*Amborella trichopoda*). Among these species, ten are eudicots and eight are monocots (**Figure 2**).

The genome-wide protein sequences of these 23 species were downloaded from Phytozome. We used OrthoFinder (V2.2.7) (Emms and Kelly, 2019) with default parameters to predict the orthologous groups. We found one orthologous group containing both PPT1 (AT5G33320) and PPT2 (AT3G01550) of *Arabidopsis thaliana*, therefore, proteins from this orthologous group were regarded as members of the PPT gene family and used to construct the gene tree of PPTs. We included triose-phosphate/phosphate translocator (TPT) from *A. thaliana* (AT5G46110) as an outgroup. All orthologous proteins of the PPT gene family together with the outgroup protein were aligned using MUSCLE (Edgar, 2004) with default parameters. The gene tree was constructed with RAXML software (Stamatakis, 2006) based on protein sequence alignment with the PROTGAMMAILG model. The robustness of the tree topology was evaluated by bootstrap scores, which were calculated from 1,000 independently constructed gene trees.

### Procedures Used to Survey Transcript Abundance of Phospho*enol*pyruvate Transporters From Published RNA-Seq Data

High transcript abundance is suggested as a major feature of genes recruited to support C_4_ functions (Moreno-Villena et al., 2018), so we tested whether this applies to PPT. Specifically, we compared the transcript abundance of PPT1 and PPT2 in leaf among species with different photosynthetic types; we also compared the expression patterns of PPT1 and PPT2 in different tissues and cell types. We surveyed RNA-seq data from four independent C_4_ lineages, namely: *Heliotropium*, *Mollugo*, *Neurachne*, and *Flaveria* available from 1 KP (http://www.onekp.com/blast.html). Except for *Neurachne*, which had RNA-seq data from shoot, RNA-seq data of other three genera were from mature leaves. RNA isolation, quality control, library preparation, and sequencing procedures are summarized in Johnson et al. (2012). Data collection information is available on the 1 KP website (http://www.onekp.com/samples/list.php?set). The RNA-seq source and quantification process for the *Flaveria* species were described in (Lyu et al., 2018). The RNA-seq analysis process for *Heliotropium*, *Mollugo*, and *Neurachne* followed the procedures used for *Flaveria*. Briefly, RNA-Seq data were generated using Illumina with a paired-end sequencing strategy with a read length of 90 bp. Transcripts were assembled using Trinity (version 2.02) (Grabherr et al., 2011) with default parameters except that the minimal length of transcript was restricted to be 300 nt.

Transcript abundance was analyzed by mapping short reads to assembled contigs of corresponding species and then normalizing the transcript abundance to the Fragments Per Kilobase of transcript per Million mapped reads (FPKM) using the RSEM package (version 1.2.10) (Li and Dewey, 2011). Functional annotations of transcripts from dicot species, namely, *Heliotropium*, *Mollugo*, and *Flaveria*, were determined by searching for the best hit in the protein dataset of *A. thaliana* in TAIR 10 (http://www.arabidopsis.org) by using BLAST in protein space with an E-value threshold of 1E−5. We annotated *Neurachne* transcripts by searching for the best hit in the protein dataset of *Zea mays* (**Table S6**). The protein sequences of *Z. mays* were downloaded from Phytozome 10.3 (http://phytozome.jgi.doe.gov/pz/portal.html). PPTs in the four genera were determined as the orthologs of PPTs of *A. thaliana* or *Z. mays*.

When comparing transcript abundance of PPTs in roots and leaves from C_3_ and C_4_ species, we surveyed processed RNA-seq data and identified species that have RNA-Seq data from both roots and leaves, which include two *Flaveria* species e.g., *Flaveria robusta* and *Flaveria trinervia* (Lyu et al., 2018), two Brassicaceae species, i.e., *Gynandropsis gynandra* and *Tarenaya hassleriana* (Kulahoglu et al., 2014), and 21 species in the grass family (Moreno-Villena et al., 2018). We also compared the transcript abundance of PPTs between BSC and whole leaf in C_3_ species, and between BSC and MC in C_4_ species based on processed RNA-seq data. Specifically, gene expression data of PPTs in BSC and whole leaf of *A. thaliana* were from (Aubry et al., 2014); gene expression data in BSC and MC of maize were from (Tausta et al., 2014); data of *G. gynandra* were from (Chang et al., 2012); data of *Setaria viridis* were from (John et al., 2014); and data of *Panicum virgatum* were from (Rao et al., 2016). The photosynthetic type and abbreviations of species are listed in **Table S1**.

### Quantification of Changes in Phospho*enol*pyruvate Transporter Transcript Levels Under Light Treatments in *Flaveria* Species Using Real-Time Quantitative-PCR

Given that the genus *Flaveria* includes species at different evolutionary stages of C_4_ photosynthesis, we further used this genus as a model to examine how the transcript abundance of PPT1 and PPT2 evolved along with the evolution of C_4_ photosynthesis. Specifically, we studied this in five species representing four different photosynthetic types, i.e., *F. robusta* (C_3_), *Flaveria sonorensis* (C_3_–C_4_), *Flaveria ramosissima* (C_3_–C_4_), *F. trinervia* (C_4_), and *Flaveria australasica* (C_4_). For the *Flaveria* species used in real-time quantitative (qRT)-PCR and the subsequent genomic study, *F. robusta* and *F. ramosissima*, *Flaveria palmeri*, and *Flaveria bidentis* were provided by Prof. Peter Westhoff (Heinrich-Heine-University); *F. sonorensis*, *F. australasica*, *F. trinervia*, *Flaveria kochiana*, and *Flaveria vaginata* were provided by Prof. Rowan F. Sage (University of Toronto). *Flaveria* plants were grown in soil in growth rooms with air temperature controlled to be 25°C, relative humidity 60%, photoperiod 16/8 h day/night, and photosynthetic photon flux density (PPFD) 500 μmol m^−2^ s^−1^. The *Flaveria* plants were watered twice a week and fertilized weekly. To study the gene expression differences of PPT1 and PPT2 in response to illumination, 1-month old plants were put into darkness at 6 pm. The dark-adapted plants were illuminated at 9:30 am the next day. Fully expanded leaves, usually the 2^nd^ or 3^rd^ leaf pair counted from the top, were cut after the leaves were illuminated for different time periods, *i.e.*, 0, 0.5, 2, and 4 h, and then flash frozen with into liquid nitrogen. Leaf samples were stored at −80°C before processing.

RNA was extracted following the protocol of the PureLink™ RNA kit (Thermo Fisher Scientific, USA). For qRT-PCR, 0.2–0.5 μg RNA was incubated with Superscript II Reverse Transcriptase (TransGen Biotech, Beijing) to obtain complementary DNA (cDNA). qRT-PCR was conducted following the manufacturer’s instructions of the UNICON™ qPCR SYBR Green Master Mix kit (YEASEM, Shanghai). cDNA, buffer, and primers were pipetted to the Hard-Shell PCR 96-well Plates (Bio-Rad, USA), and covered by MicroSeal ‘B’ Seal (Bio-Rad, USA). qRT-PCR was run in the BIO-RAD CFX connect system (Bio-Rad, USA). Relative transcript abundance was calculated by comparing to ACTIN7 and data were processed using the BIO-RAD CFX Maestro software (Bio-Rad, USA). For each gene, three technical and three biological replicates were performed. The primers used here are listed in **Table S2**.

### Prediction of Gene Structure and *cis*-Elements of PPT1 and PPT2 From *Flaveria* Species

The promoter sequences of PPT1 and PPT2 from four *Flaveria* species, namely, *F. robusta*, *F. sonorensis*, *F. ramosissima*, and *F. trinervia*, were obtained from the draft genome sequences of the four species. In order to detect the genomic loci of PPT1 and PPT2, we performed a BLAST search against the genome sequence by using the coding sequences (CDS) of PPT1 and PPT2 in each species as a query sequence and applying BLAST+ (v2.2.31) (Camacho et al., 2009) with E-value < 1E−5. A candidate locus of a gene is manually selected if it reports a series of gapped mapping regions with identity higher than 95%, where mapping regions represent exons and gaps represent introns. The protein sequences of PPTs from the four *Flaveria* species and *A. thaliana* were aligned with MUSCLE (Edgar, 2004), based on which the gene tree was inferred with RAXML software (Stamatakis, 2006) using the same procedure described above.

In order to quantify the transcript abundance of PPT orthologs, we generated RNA-seq data for these four species. The growth conditions of *Flaveria* plants and RNA isolation procedures were the same as those used for the qRT-PCR experiment described above. The cDNA library was constructed with NEBNext Ultra II RNA Kit (New England Biolabs, USA). RNA-seq was performed with the Illumina NovaSeq 6000 platform in the paired-end mode with a read length of 150 bp. The data were submitted to gene expression omnibus (GEO) with the accession number GSE143469. We mapped the RNA-seq reads to genome sequence of each species using STAR (V2.7) (Dobin et al., 2013) and calculated the gene expression in Transcripts per kilobase Per Million mapped reads (TPM) using RSEM (V1.3.3) (Li and Dewey, 2011). We verified the promoter sequences of the copies of PTP1 and PPT2 that showed relatively high transcript abundance in each species by PCR and sequencing. The primers used here are listed in **Table S2**.

The draft genome sequences of the four species were submitted to the National Center for Biotechnology Information (NCBI) with accession number SAMN14943594 for *F. robusta*, SAMN14943595 for *F. sonorensis*, SAMN14943596 for *F. ramosissima* and SAMN14943598 for *F. trinervia*.

### Comparison of the Amino Acid Sequences of PPT1 and PPT2

The amino acid sequences of PPT1 and PPT2 of different *Flaveria* species were predicted based on *de novo* assembled transcripts as described in (Lyu et al., 2018). Protein sequences of orthologs were aligned with MUSCLE (Edgar, 2004). We further identified consistent amino acid modifications between C_3_ and C_4_ species, which were defined as sites that showed differences between C_3_ and C_4_ species, but that were conserved within C_3_ species and also conserved within C_4_ species. These consistently identified modifications were mapped to the phylogenetic tree of *Flaveria* (Lyu et al., 2015) to identify the evolutionary stage of their appearance during C_4_ evolution in *Flaveria*. With the protein sequence information, we predicted the 3D protein structures of PPT1 of *Flaveria* species using the Iterative Threading ASSEmbly Refinement (I-TASSER) online server (Yang and Zhang, 2015).

We further tested whether PPT1 and PPT2 experienced positive selection in C_4_ species using C_3_ species and intermediate species as background. First, amino acid sequences of orthologous genes were aligned with the software MUSCLE (Edgar, 2004). Aligned protein sequences were then used to guide the codon-wise alignment of CDS with PAL2NAL (Suyama et al., 2006). After gaps and stop codons were removed, the aligned sequences were input into the PAML package (V4.8) (Yang, 2007) for positive selection tests. Phylogeny of the *Flaveria* species was inferred from our previous work (Lyu et al., 2015). Considering that the phylogeny of *Flaveria* contains two clades, we conducted the positive selection in two independent ways: either including species of both clade A and clade B, or excluding species from clade B which lacks a true C_4_ species. In this study, the positive selection test was conducted using a branch-site model (model=2, NSsites=2) under an equal nucleotide substitution condition (CodonFreq=0, all frequencies were fixed to be 1/61). The likelihood of the null hypothesis was calculated under this branch-site model with fixed dN/dS ratio (ω=1, neutral). The maximum likelihood of the alternative hypothesis was calculated under this branch-site model with flexible dN/dS ratio (ω > 1, positive selection). Then, the likelihood ratio test (LRT) was conducted between the null hypothesis and the alternative hypothesis under the chi-square distribution to accept or reject the alternative hypothesis with the “chi2” function in the PAML package. A threshold *p*-value of 0.05 [Benjamini Hochberg (BH) adjusted] was used to determine positive selection in C_4_ species.

To investigate the copy number of 13-aa elements in different *Flaveria* species, DNA was extracted from the 2^nd^ or 3^rd^ pair of leaves counted from the top following the protocol of TIANquick Midi Purification kit (TIANGEN Biotech, Beijing). The primers are listed in **Table S2**.

### Determining the Subcellular Localization of *Flaveria* PPT1 and PPT2

We further tested whether the subcellular localization of PPT1 and PPT2 are conserved between C_3_ and C_4_ species in the *Flaveria* genus. To determine the subcellular localizations of PPT1 and PPT2 from *Flaveria* C_3_ and C_4_ species, we generated fluorescence fusion proteins by tagging a green fluorescent protein (GFP) in the C-terminal end of PPTs and transiently expressed them in *Nicotiana benthamiana* (tobacco) leaves. Specifically, the CDS of PPT1 and PPT2 were amplified from cDNAs reverse transcribed from RNAs for *F. bidentis* (C_4_), and from *de novo* synthesized DNA for *F. robusta* (C_3_) by Shanghai Personalbio LLC by PCR. A CDS with the 52-amino acid (52 aa) insertion deleted, i.e., ΔFbid-PPT1, was generated *via* overlapping PCR. All primers are listed in **Table S2**. All the PCR fragments of PPT1 and PPT2 were integrated into the binary vector pCAMBIA1302 *via* homologous recombination-based in-fusion cloning (GBClonart). The promoter used was a CaMV 35S promoter. The final plasmids were verified by Sanger-sequencing (Sangon Biotech, Shanghai). The verified vectors were transformed into *Agrobacterium tumefaciens* (Agrobacterium) strain GV3101 competent cells (TransGen Biotech, Beijing). The Agrobacterium cells were cultured in liquid Luria-Bertani (LB) medium containing rifamycin and kanamycin and re-suspended in infiltration buffer [10 mM 2-(N-morpholino)ethanesulfonic acid (MES) pH5.7, 10 mM MgCl_2_, 200 μM acetosyringone] to OD_600_ ~1.0. The Agrobacterium cells were inﬁltrated into tobacco leaves with a syringe. After 36 to 48 h, the ﬂuorescence signals from leaf pavement cells were examined using a confocal fluorescence microscope (Zeiss LSM880, Germany). The autofluorescence signal from chlorophyll was used as a marker for chloroplast thylakoids, with an excitation wavelength of 488 nm and an emission wavelenth of 507 nm.

### Testing the Functional Conservation of PPT1 Between C_3_ and C_4_ Species

Finally, we tested whether the PEP transporting function of PPT1 was conserved between C_3_ and C_4_ species. We asked whether a C_4_ PPT1 can rescue the phenotype of a C_3_ PPT1 mutant. The *A. thaliana* PPT1 mutant *cue1-5*, which is an ethyl methanesulfonate (EMS) mutant harboring the R81C mutation in PPT1, was ordered from North American Services Center (NASC) (stock number N3156). Then, we introduced different *Flaveria* PPT1-GFP driven by the *35S* promoter into *cue1-5* mutant *via* an Agrobacterium-mediated ﬂoral dipping method. Agrobacterium cells transformed with the binary plasmids were cultured in Luria-Bertani media at 28°C for 48 h. Agrobacterium cells were pelleted and re-suspended in transformation buffer (50 g sucrose, 2.2 g Murashige and Skoog powder, 200 μl Silwet L-77, and 10 μl 6-BA for 1L, pH 5.8 to 6.0) to OD_600_ ~1.0. The *A. thaliana* flowers were dipped in buffer containing Agrobacterium cells and kept for 5 min, and then the plants were put under dark overnight. The floral dipping process was repeated 1 week later. After maturation, the seeds were collected and screened on ½ Murashige and Skoog (MS) agar plates containing hygromycin at a concentration of 35 mg/L. The positive T_1_ transformants were transferred to soil. The T_2_ lines were used to examine morphological phenotypes. The plants were grown in a growth chamber with a long-day condition (16 light/8 dark), a PPFD of ~100 μmol m^−2^ s^−1^, and temperature cycle of 23°C during the day and 21°C at night.

## Results

### The Evolution of Phospho*enol*pyruvate Transporter in the Viridiplantae

To investigate the evolution of the PPT, we first constructed a phylogenetic tree for PPT orthologs from 23 species in Viridiplantae (**Figure 2**). These species were selected to capture the major events in Viridiplantae evolution with one species representing a major evolutionary stage of the Viridiplantae phylogeny. Furthermore, we included 10 eudicot species with six species from the Brassicaceae family and eight monocot species with seven species from the grass family (**Figure 2**). These two families contain the most sequenced genomes; hence they can be used to study how PPTs evolved within families.

The gene tree showed that PPTs were present in all selected species. PPT had one copy in species that evolved before angiosperms, including the two chlorophyte species, *M. pusilla* and *C. reinhardtii*, as well as *M. polymorpha* and *S. moellendorffii*. The angiosperm species *A. trichopoda* also has one copy. In contrast, there were two copies in other angiosperms with one being the ortholog of *A. thaliana* PPT1 and another being the ortholog of *A. thaliana* PPT2 (**Figure 2A** and **Figure S1**). PPTs from lower species showed higher similarity with *A. thaliana* PPT1 than *A. thaliana* PPT2. Furthermore the single copy of PPT in *A. trichopoda* was in the PPT1 lineage of dicots, suggesting that PPT1 was the ancestral copy and PPT2 was a derived copy that originated after the split of *A. trichopoda* from other angiosperm species (**Figure 2B**). There were one or two copies of PPT1 and a single copy of PPT2 in dicot species, whereas there were two or three copies of PPT1 and one or two copies of PPT2 in grass species, consistent with an extra whole genome duplication (WGD) event in monocot species (Jiao et al., 2014). We observed more rapid evolution of PPT2 compared to PPT1; furthermore, PPT2 in dicots, especially in Brassicaceae, showed faster evolution than in monocots. The physiological significance and underlying mechanisms behind these different evolutionary speeds are unknown.

### The Evolution of Phospho*enol*pyruvate Transporters in Transcript Abundance and Tissue Specificity of Expression Along the Emergence of C_4_ Species

**Figure 3:**
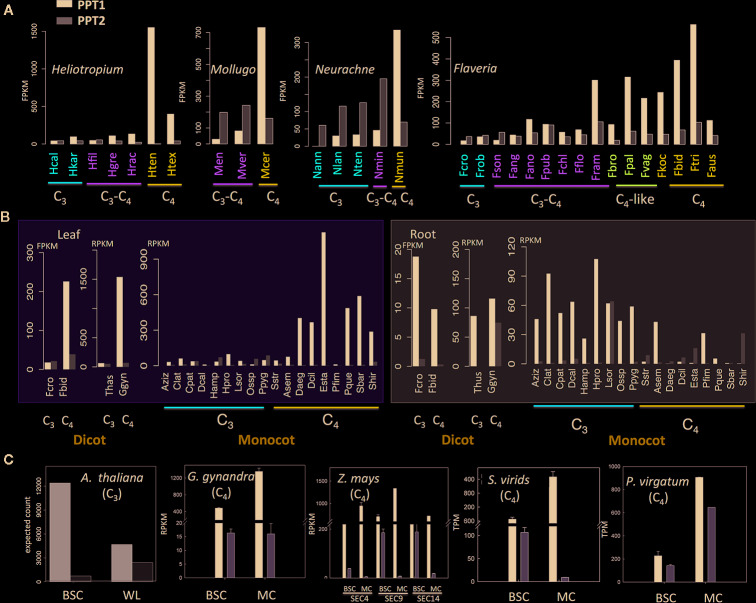
The evolution of transcript abundance of PPT1 and PPT2 from C_3_ to C_4_ species **(A)** The transcript abundance of PPT1 and PPT2 along the evolution of C_4_ photosynthesis in four genera. Photosynthetic types are marked with different colors, red: C_3_; green: C_3_–C_4_; purple: C_4_-like; blue: C_4_. **(B)** The transcript abundance of PPT1 and PPT2 in leaf and root. **(C)** The transcript abundances of PPT1 and PPT2 in BSC and whole leaf or MC in one C_3_ species (*Arabidopsis thaliana*) and four C_4_ species. All the data are from published RNA-seq data; the data source is detailed in the *Materials and Methods* section. (Abbreviations: BSC, bundle sheath cell; WL, whole leaf; MC, mesophyll cell. Species abbreviations are listed in **Table S1**.)

We further compared the expression abundance between PPT1 and PPT2. First, we examined the transcript abundances of PPT1 and PPT2 in a few sets of species that are evolutionarily closely related but have different photosynthetic types. These species are from four genera with each representing an independent C_4_ lineage. Among these four genera, three are dicots, i.e., *Flaveria*, *Heliotropium*, and *Mollugo*, and one is a monocot, i.e., *Neurachne* (**Figure 3A**). The RNA-seq data for the *Flaveria* species are from the 1,000 plants project (1 KP) (Matasci et al., 2014) and (Mallmann et al., 2014), with data from leaf samples, and have been demonstrated to be comparable in FPKM (Lyu et al., 2018). RNA-seq data for the other three genera are also from 1 KP (Matasci et al., 2014). In the analysis, data from mature leaves were used. The comparison showed that in C_3_ species, PPT2 displayed higher transcript abundance than PPT1 except in the two C_3_ species in *Heliotropium*, namely, *Heliotropium calcicola* (Hcal) and *Heliotropium karwinsky* (Hkar) (**Figure 3A**). The higher expression of PPT2 over PPT1 was also shown in C_3_–C_4_ species of *Mollugo* and *Neurachne*, as well as in C_3_–C_4_ species in *Heliotropium,* i.e., *Heliotropium filiforme* (Hfil) and *Flaveria* i.e., *F. sonorensis* (Fson) (**Figure 3A**). During the transition from C_3_ to C_4_ photosynthesis, however, we observed an increase of transcript abundance in PPT1 while the transcript abundance of PPT2 remained similar, resulting in a higher expression abundance of PPT1 compared to PPT2 in C_4_ species.

Though PPT1 in leaves of C_3_ species did not show higher transcript abundance than PPT2, for the dicot C_3_ species, PPT1 showed higher transcript abundance than PPT2 in root (**Figure 3B**); the same pattern was also found in most monocot C_3_ species with *Lasiacis sorghoidea* (Lsor) as an exception (**Figure 3B**). In C_4_ monocot species, the transcript abundance of PPT1 was not always higher than that of PPT2 in root; furthermore, the PPT expression levels in root were generally lower in C_4_ as compared to C_3_ species (**Figure 3B**). Therefore, PPT1, the copy recruited to support C_4_ photosynthesis, did not have higher expression levels than PPT2 in the leaf tissue of C_3_ plants; however, the gene had higher transcript abundance than PPT2 in root. During the evolution of C_4_ photosynthesis, the transcript abundance of PPT1 was decreased in root and increased in leaf, implying a major shift in tissue specificity.

Considering that C_4_ photosynthesis occurs in two cell types, which is a major evolutionary innovation, we further examined the changes in cellular specificity of PPT expression during C_4_ evolution. For this purpose, we compared the transcript abundance of PPT1 and PPT2 in BSC and whole leaf in one C_3_ species and that of BSC and MC in four C_4_ species (**Figure 3C**). RNA-seq data from transcript residency on ribosomes (Aubry et al., 2014) shows that PPT1 had a higher expression level in BSC than in the whole leaf in *A. thaliana*, whereas PPT2 displayed the opposite pattern, which is consistent with earlier histochemical localization of the PPT promoter (Knappe et al., 2003): PPT1 localized in BSC and root, especially in root tip, while PPT2 localized in MC. In all C_4_ species examined in this study, the transcript abundance of PPT1 was consistently higher in MC than in BSC (**Figure 3C**). In contrast, PPT2 showed no clear cell type specificity between the two cell types (**Figure 3C**). Therefore, during the evolution of C_4_ photosynthesis, PPT1 shifted its cellular specificity from dominantly BSC to dominantly MC.

We also examined the expression patterns of PPTs based on transcriptomic data available in GENEVESTIGATOR (March, 2018), which includes four C_3_ species, i.e., *A. thaliana*, *Oryza sativa*, *Solanum lycopersicum*, and *Glycine max*, and two C_4_ species, i.e., *Z. mays* and *Sorghum bicolor*. We investigated the expression with a focus on developmental scale, in which the average was calculated from samples at the same development stage regardless of tissue type and cell type, and on the scale of cell type. PPT1 showed higher expression than PPT2 in general based on developmental stage (**Figure S2**). In C_3_ species, either PPT2 or PPT1 together with PPT2 showed high expression in leaf, whereas PPT1 was dominant in root, with an exception in rice, in which PPT1 and PPT2 had comparable transcript levels (**Figure S2**). The dominant role of PPT1 was more obvious in root tip in C_3_ species. In C_4_ species, the expression patterns of PPT1 and PPT2 switched between leaf and root and between MC and BSC, which is in line with the above results.

### The Changes in Transcriptional Regulation of Phospho*enol*pyruvate Transporter During Evolution From C_3_ to C_4_ Photosynthesis

**Figure 4:**
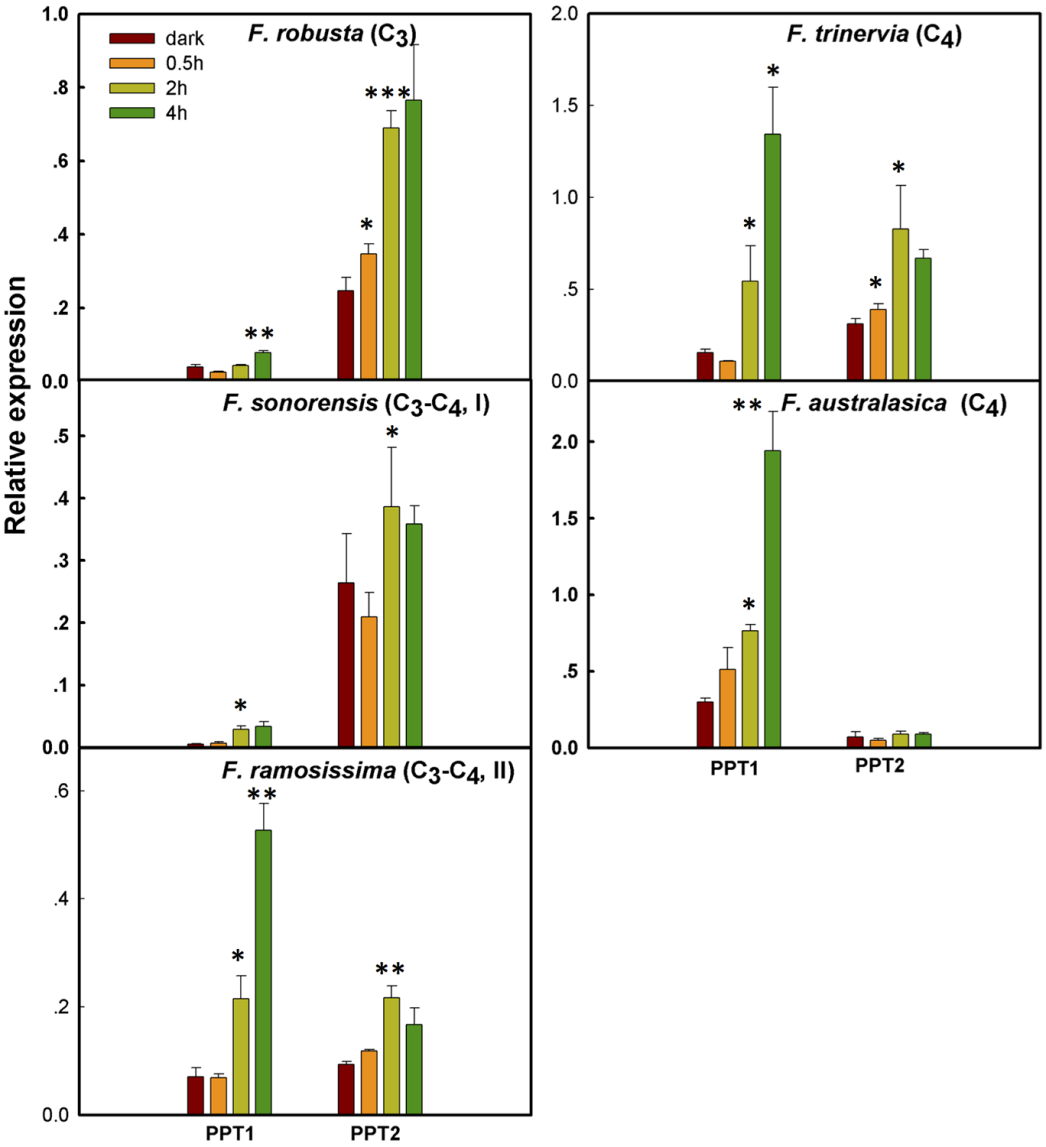
The change in light responsiveness of PPT1 and PPT2 along the evolution of C_4_ photosynthesis in the genus *Flaveria* Real-time quantitative (qRT)-PCR was used to quantify the transcript abundance of PPT1 and PPT2 in mature leaves after 0, 0.5, 2, and 4 h upon illumination. Significance levels represent the significance of the differences between the transcript abundance at a time point compared to that at the preceding time point (*t-*test; *: 0.05–0.01, **: 0.01–0.001, ***: < 0.001).

The mechanism by which PPT1 gained new expression patterns to support C_4_ photosynthesis, e.g., shifting its tissue specificity from primarily in root to primarily in leaf, and shifting its cellular specificity from predominantly in BSC to predominantly in MC is unknown. Examination of the expression patterns of PPTs between BSC and MC in four segments of maize shows that PPT1 has higher transcript abundance in MC than in BSC (**Figure 3C**). Given that the leaf MC typically receives more light than BSC (Xiao et al., 2016), one possibility is that the C_4_ PPT1 might have acquired light-responsive *cis-*elements, which enables PPT1 to show light-dependent transcript accumulation patterns. To test the possibility, we first examined the light responsiveness of PPT1 and PPT2 along the C_4_ phylogeny. Specifically, we compared the transcript abundance of PPT1 and PPT2 in mature leaves after 0, 0.5, 2, and 4 h of illumination. We quantified the transcript abundance using qRT-PCR in five *Flaveria* species, representing different photosynthetic types, *i.e.*, C_3_ photosynthesis, *F. robusta*; type I C_3_–C_4_ species, *F. sonorensis*; type II C_3_–C_4_ species, *F. ramosissima*; and C_4_ species, *F. trinervia* and *F. australasica* (Sage et al., 2012) (**Figure 4**). Our results demonstrated a gradual increase in the speed of changes of PPT1 transcript abundance to light from C_3_ to C_3_–C_4_ intermediate to C_4_ species. Specifically, the transcript abundance of PPT1 did not show significant up-regulation (P < 0.05, *t*-test) until 4 h under illumination in the C_3_ *F. robusta*, whereas significant up-regulation of PPT1 transcript abundance was observed at 2 h in C_3_–C_4_ species. In the C_4_ species *F. australasica*, the transcript abundance of PPT1 was up-regulated at 0.5 h under illumination with marginal significance (P=0.075, *t*-test). Therefore, during C_4_ evolution, PPT1 acquired new mechanisms enabling it to be rapidly up-regulated upon illumination.

We further examined the patterns of increase in transcript abundance of PPT upon illumination change along the evolution from C_3_ to C_4_ species. Type I C_3_–C_4_ species showed the maximal PPT1 transcript abundance at 2 h under illumination, while the transcript abundance of PPT1 in type II C_3_–C_4_ and C_4_ species kept increasing even after 4 h under illumination (**Figure 4**). The light responsiveness of PPT2 showed an opposite pattern as compared to PPT1 along C_4_ evolution. Specifically, in the C_3_ *F. robusta*, PPT2 showed significantly higher transcript abundance than PPT1. An up-regulated expression level of PPT2 in *F. robusta* was observed at 0.5 h under illumination, and a further increase was observed until 2 h. Nevertheless, in both C_3_–C_4_ species and the C_4_ species *F. trinervia*, significantly increased expression of PPT2 was not detected until 2 h under illumination. Although PPT2 was induced at 0.5 h under light in C_4_ species *F. trinervia*, in another C_4_ species *F. australasica*, the transcript abundance of PPT2 showed no significant up-regulation under the illumination. Therefore, during evolution PPT1 gained not only higher transcript abundance in leaf, in particular in the MC, but also a more rapid and long-lasting response to light illumination, while PPT2 gradually lost its light responsiveness.

### C_4_ PPT1 Promoter Acquired MEM1 B Submodule But Not in C_3_ and C_3_–C_4_ Species

**Figure 5:**
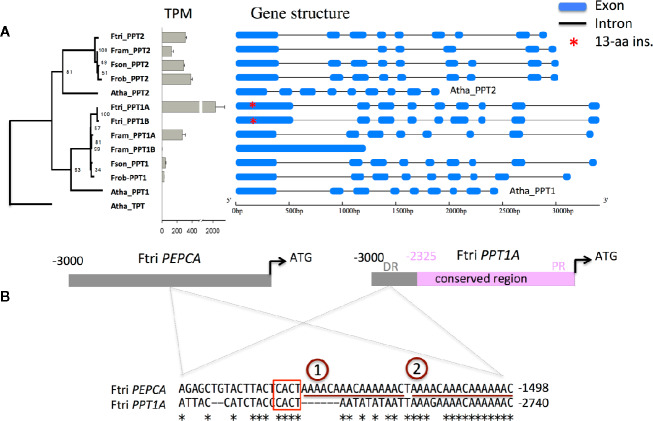
Promoter of C_4_ PPT1 has a MEM1 B submodule that might have contributed to the increased transcript abundance of PPT1 **(A)** Shows transcript abundance and gene structure of PPT1 and PPT2 in four *Flaveria* species. The gene tree of PPTs was constructed based on the alignment of protein sequences of PPT1 and PPT2 using a maximum likelihood method. Numbers on each node show bootstrap scores from 1,000 independent simulated trees. Transcript levels of *Flaveria* PPT1 and PPT2 based on RNA-seq data were shown in Transcript per kilobase Per Million mapped reads (TPM) in grey bars with error bar showing standard error estimated based on three replicates. *Flaveria trinervia* and *Flaveria ramosissima* have two copies of PPT1 but only one of the copies has TPM higher than 2. The 4*13-aa insertion occurred at the first exon of PPT1 in *F. trinervia* and is shown as red star. **(B)** The MEM1 B submodule of *PEPCA* gene promoter from *F. trinervia* is also present in the promoter of PPT1 from *F. trinervia*. The sequence alignment shows the MEM1 B submodule of the *PEPCA* from *Flaveria rinervia*, which has two copies of “AAAACAAACAAAAAC.” Asterisks represent identical nucleotides. (Abbreviations: Ftri, *F. trinervia*; Fram, *F. ramosissima*; Fson, *F. sonorensis*; Frob, *F. robusta*; Atha, *A. thaliana*; DR, distal region; PR, proximal region.)

Changes in transcriptional responses to external stimuli can be driven by changes in gene regulatory mechanisms. We tested whether C_4_ PPT1 might have acquired new *cis*-elements that are responsible for the altered expression patterns. Based on the draft genome sequences of four *Flaveria* species, we found that there are two copies of PPT1 in *F. ramosisisma* (C_3_–C_4_, II) and *F. trinervia* (C_4_), and one copy in *F. robusta* (C_3_) and *F. sonorensis* (C_3_–C_4_, I). The promoter sequences (3 kbp upstream of the start codon) of two *F. trinervia* PPT1 are same, however, only one of these two copies was expressed. This copy also showed the highest expression level among the four species (with a TPM of 2,053); we name this copy *PPT1A* (**Figure 5A**). In *F. ramosissima*, one of the two copies of PPT1 has no intron and showed very low transcript abundance with a TPM of 1 (*PPT1B*), while the TPM of another copy was 272 (*PPT1A*). Moreover, the promoter sequences of the two PPT1s from *F. ramosissima* are not conserved with a sequence identity of only 11%. For PPT2, all species have one copy, which also show comparable transcript abundance in the four species (**Figure 5A**). In terms of genomic structure, both PPT1 and PPT2 have nine exons in most *Flaveria* species and *A. thaliana*, with the exception that in *F. ramosissima* there are eight exons in PPT1 and six exons in PPT2 (**Figure 5A**).

Further examination of the promoter structure shows that there is a highly conserved region between the proximal region of PPT1 promoter from *F. trinervia* (−2,325 to −1 bp upstream from the start codon) and that from *F. ramosissima*. The conserved region was divided into two parts by an insertion in *F. ramosissima*. Moreover, the two conserved parts were also observed in the promoters of PPT1 from *F. robusta* and *F. sonorensis* (**Supplemental File 3**). We further found a mesophyll expression module (MEM1) B submodule at the distal region (−2,783 to −2740 bp from the start codon) of the PPT1 promoter from *F. trinervia*, but this MEM1 B submodule was not present either in the counterpart in PPT1 from the other three *Flaveria* species, or the counterpart in PPT2 from any tested *Flaveria* species (**Figure 5B** and **Supplemental File 3**). The MEM1 B submodule is responsible for the increased expression level of the *PEPCA* (Akyildiz et al., 2007) and the *CA3* in *Flaveria* C_4_ species, therefore, the same MEM1 B *cis*-element in the promoter of C_4_ PPT1 might confer its higher expression. However, neither submodule A of MEM1, which is present in *PEPCA* (Akyildiz et al., 2007), nor submodule A of MEM1-like, which is present in *CA3* (Akyildiz et al., 2007), was observed in the C_4_ *PPT1A* promoter. It is possible that other *cis*-element(s) that have same function with the A submodule may also be present in the promoter of C_4_ PPT1 to realize the specific expression of PPT1 in MC.

### Changes in the Physiological Functions of Phospho*enol*pyruvate Transporters During C_4_ Evolution

**Figure 6:**
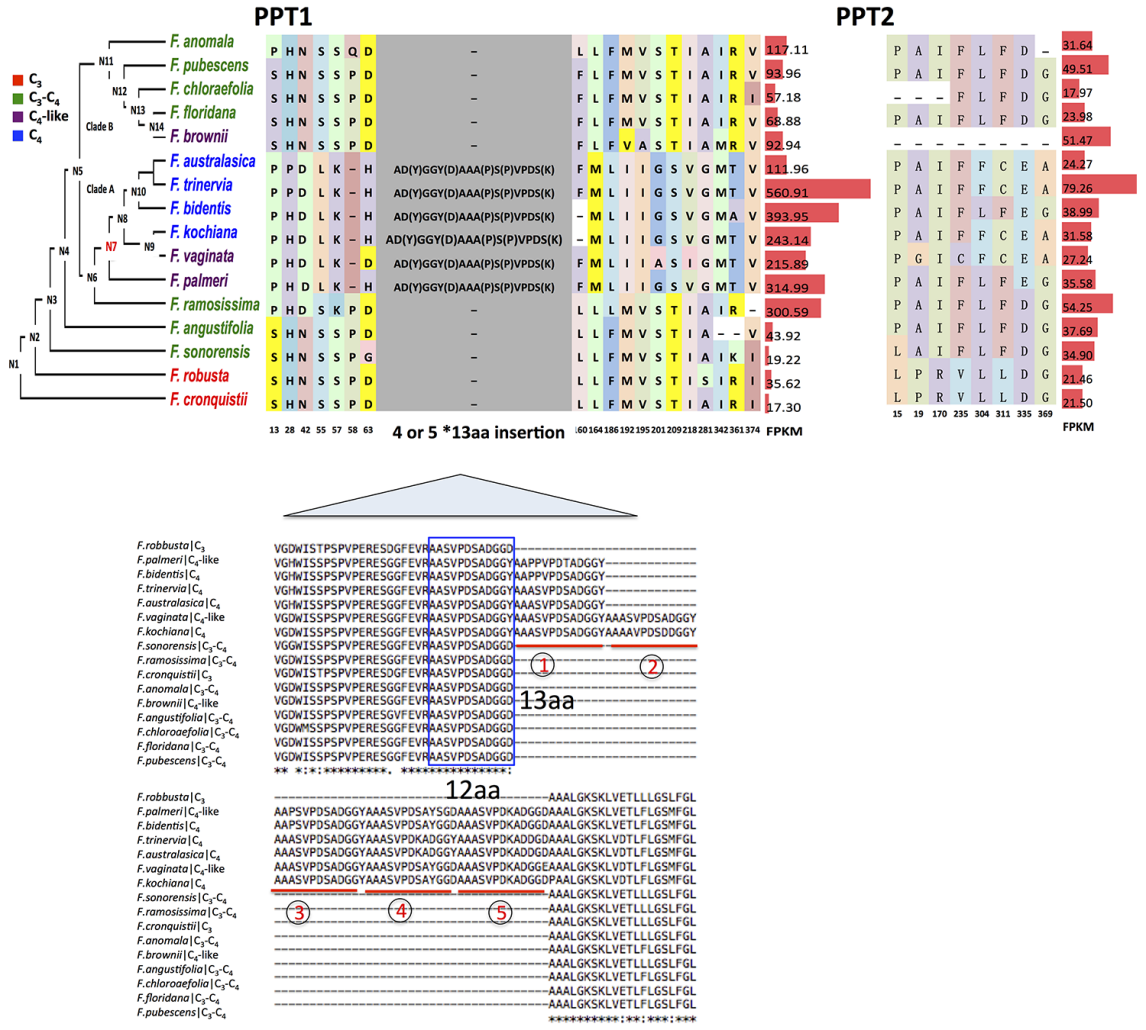
The comparison of PPT1 and PPT2 protein sequences in the genus *Flaveria* Amino acid changes of PPT1 and PPT2 among different species in *Flaveria* genus are shown on the phylogeny. Transcript abundance calculated as Fragments Per Kilobase of transcript per Million reads mapped (FPKM) is displayed on the right with red bars. Numbers below amino acids are the aligned locations. The “-” symbols show an alignment gap. PPT1 (406 amino acids in *Flaveria cronquistii*) shows more frequent amino acid changes than PPT2 (417 amino acids in *F. cronquistii*). An insertion composed of four or five 13-aa elements occurs at the ancestral node “N7” (marked in red) on the phylogenetic tree. The sequence of the 13-aa segment is variant AAA(P)SVPDS(K)AD(Y)GGY(D) at four sites. A 12-aa element (blue frame) at the N-terminal end of the insertion is present in all species, which should be the origin of the 13-aa element.

We finally examined the functional changes of PPTs between C_3_ and C_4_ species. Usually the functional changes of a protein are underlined by changes in the amino acid sequence. Here we examined the changes in amino acid sequences of PPT1 and PPT2 from 16 species in the genus *Flaveria*, which covers C_3_, C_3_–C_4_, C_4_-like and C_4_ species. Because of a lack of genome reference for some *Flaveria* species, the protein sequences of PPT1 and PPT2 were predicted based on *de novo* assembled transcripts for those species, and the genes that showed the highest sequence similarity with PPT1 and PPT2 from *F. robusta* were selected for comparison; therefore, only one copy of PPT1 and PPT2 from each *Flaveria* species were compared. We specifically examined the number of consistent amino acid modifications, which were defined as sites that have the same amino acid sequences in C_4_ species but differ with those in C_3_ species. The results show that PPT1 had more consistent amino acid modifications than PPT2 when the sequences from C_4_ and C_3_ species were compared. Specifically, the amino acid sequence of PPT1 had 19 consistent amino acid modifications between C_3_ and C_4_ species; in contrast, PPT2 exhibited eight consistent amino acid modifications (**Figure 6**). To test whether these modifications were specific adaptations gained during evolution of C_4_ photosynthesis, we performed a positive selection test in protein coding sequences of C_4_ species against that of C_3_ species in the genus *Flaveria*. PPT2 showed a signal of positive selection in C_4_ species; however, the two predicted positive selected sites of PPT2 were neither C_4_ specific nor C_4_ consistent modifications (**Figure S3**). In contrast, PPT1 showed no signal of positive selection in C_4_ species, suggesting that the consistent mutations observed in C_4_ species may occur by chance during evolution. Though we did not identify any particular amino acid sequence under positive selection, PPT1 however showed a large insertion acquired at the common ancestor of C_4_-like and C_4_ species in clade A. The insertion segments had either four (*F. palmeri*, *F. bidentis*, *F. trinervia*, and *F. australasica*) or five (*F. vaginata* and *F. kochiana*) repeats with each repeat comprising 13 amino acids, i.e., a 13-aa element (**Figure S4**).

To investigate whether the 13-aa element is also present in PPT1 of other C_3_ and C_4_ species, we compared amino acid sequences of PPT1 from other C_3_ and C_4_ species, including three C_3_ species, namely, *A. thaliana*, *T. hassleriana* and *F. robusta*, and six C_4_ species, *F. bidentis*, *F. trinervia*, *G. gynandra*, *S. bicolor*, *Z. mays*, and *S. italica*. The alignment shows that the 13-aa-element insertion is only present in *Flaveria* C_4_ species (**Figure S5**). We determined that the insertion might have been generated by slipping mispairing during DNA synthesis as reported in *Z. mays* (Wessler et al., 1990) (**Figure S6**). We found that the 12-aa segment in *F. ramosissima* missed one alanine at the N-terminal end compared to the 13-aa segment (**Figure 6**). The DNA sequence encoding the 12-aa segment could form a stable hairpin structure (**Figure S6A**). Coincidently, there is a triplet alanine following the C-terminal end of the 12-aa segment (**Figure 6** and **Figure S6A**). In the coding sequence of the 12-aa segment and the triplet alanine (termed 15-aa segment), a 6-bp nucleotide sequence “GCGGCG” appears both at the head and the tail of the 15-aa segment. It is possible that the hairpin structure may shorten the distance between the “GCGGCG” at the 5′ end and “GCGGCG” at the 3′ end (**Figure S6B**), which facilitated “slipping mispairing” during DNA synthesis resulting in formation of the 4x13-aa insertion (**Figure S6C**).

**Figure 7:**
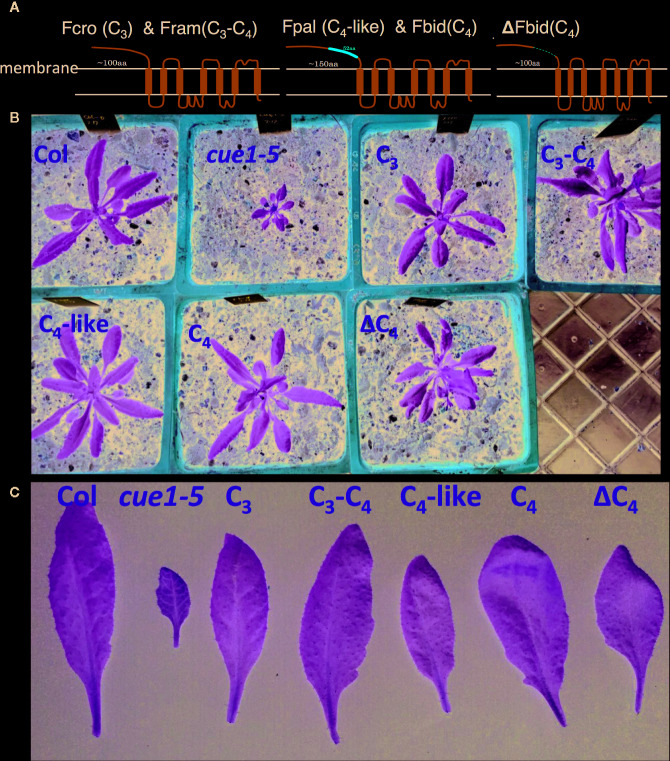
*Flaveria* PPT1 complements the phenotype of *A. thaliana* PPT1 loss-of-function mutant *cue1-5* **(A)** The position of a 52-aa insertion in protein sequence of *Flaveria bidentis* (C_4_) PPT1. The insertion is predicted to be located at the non-membrane-portion. **(B, C)** *Arabidopsis thaliana cue1-5* shows reticulate leaf phenotype and decreased rosette size; PPT1 from different photosynthetic types of *Flaveria* species rescues the phenotype of *A. thaliana cue1-5*. PPT1 from *F. bidentis* without the insertion (ΔC_4_) also recovers the phenotype of the *A. thaliana cue1-5*. (Abbreviations: Fcro, *F. cronquistii*; Fram, *F. ramosissima*; Fpal, *F. palmeri*; Fbid, *F. bidentis*.)

Given these changes of amino acid sequences in PPT1 during C_4_ evolution, we tested whether the function of PPT1 was conserved between C_3_ and C_4_ species using a genetic approach by expressing *Flaveria* PPT1 in a C_3_ *A. thaliana* PPT1 loss-of-function mutant *cue1-5* (Li et al., 1995). Specifically, we expressed PPT1-GFP driven by a *35S* promoter in *cue1-5* through gene engineering (**Figure 7A**). The PPT1 used was from four different *Flaveria* species, including one C_3_ species *Flaveria cronquistii*, two intermediate species *F. ramosissima* (C_3_–C_4_) and *F. palmeri* (C_4_-like), and one C_4_ species *F. bidentis* (C_4_). The results showed that the PEP transporting function of PPT1 from all four species complemented the reticulate leaf phenotype and small rosette size of *cue1-5* (**Figures 7B, C**), indicating that PPT1 was functionally conserved in leaf in these different *Flaveria* species and *A. thaliana*. One caveat is that the 35S promoter might not be sensitive enough to detect potential differences in the affinities of PPT1 from different species. So, there still might be physiological significance of the altered amino acids in the PPT1 sequence, which needs detailed enzymatic studies to elucidate.

Given that there exists an insertion in the C_4_ and C_4_-like species in the *Flaveria* genus in the protein-coding region of PPT1, we further tested whether this insertion affected the function of PPT1. We explored this question by removing the 4x13-aa insertion in *F. bidentis* (C_4_) (ΔFbidPPT1 for short) and expressing it in *cue1-5 A. thaliana* (**Figure 7A**). The transgenic plant *ΔFbidPPT1*/*cue1* showed the same phenotype as *FbidPPT1*/*cue1* (**Figures 7B, C**), suggesting that the insertion had no effect on the PEP transport function of PPT1 in C_3_ leaf. It was likely, therefore, that this extra insertion had no influence on the structure of PPT1 in the thylakoid membrane. Indeed, protein structure prediction using I-TASSER showed that the insertion site lies in the outer membrane portion of FbidPPT1 (**Figure 7A**), which might not influence the functional path required for PEP transport in PPT1 in the thylakoid membrane.

Considering that coding sequences can potentially harbor *cis-*elements responsible for cell specificity, as in NAD-ME in *G. gynandra* (Brown et al., 2011), we further checked whether the extra insertion affects the subcellular location of PPT1 by using transient expression of PPT1-GFP in tobacco leaves. Transient transgenic experiments showed that both FbidPPT1 and ΔFbidPPT1 were localized in chloroplasts (**Figures S7A–C**), suggesting that the insertion had no impact on the subcellular localization of PPT1. Further experiments showed that PPT1 and PPT2 from both C_3_ and C_4_ *Flaveria* species were localized to chloroplast (**Figure S7**). Therefore, all the results from sequence analysis and functional tests based on transgenic experiments suggested that though there were major changes in the amino acid sequences in PPT1 during C_3_ to C_4_ evolution, these changes neither changed the PEP transporting function of PPT1, nor altered the localization of PPT1. The potential role of these changes may be involved in the transport efficiency of PPT1, which needs more detailed biochemical studies.

## Discussion

This study presented a comparative survey of PPT, one of the metabolite transporters involved in C_4_ photosynthesis. The analysis showed that though PPT1 had lower transcript abundance in leaf compared to PPT2, it was recruited to support C_4_ photosynthesis in multiple C_4_ lineages. During C_4_ evolution, PP1 switched its expression from predominantly in root to in leaf and from predominately in BSC to in MC; it also acquired increased responsiveness of expression to light induction, which might be related to a newly recruited MEM1 B submodule in the PPT1 promoter in the *Flaveria* C_4_ species. PPT1 also shows major changes in amino acid sequences during C_4_ evolution, though they do not change the PEP transporting function. In this section, we discuss these findings in terms of their implications for C_4_ photosynthesis.

### Factors Contributing to Recruitment of PPT1 Instead of PPT2 for C_4_ Function

*Potential of high expression*: studies on the evolution of C_4_ genes identified a number of properties associated with the recruited paralogs for C_4_ function, which include relatively high expression levels (Moreno-Villena et al., 2018), availability of gene copies, which provide a fast route to increase gene expression (Bianconi et al., 2018), and suitable enzyme catalytic properties *via* accumulated mutations in the coding region (Christin et al., 2013). In the case of PPT, in terms of transcript abundance, though PPT1 had lower transcript abundance than PPT2 in leaves, PPT1 had very high transcript abundance in root, especially in the root tip (**Figure 3** and **Figure S2**). Based on the data from GENEVESTIGATOR, the total transcript abundance of PPT1 was higher than PPT2 regardless of tissue type (**Figure S2**), suggesting that some pre-existing regulatory mechanism can confer higher transcript abundance of PPT1 than PPT2. On the other hand, we found that PPT1 had a higher or the same copy number with PPT2 in angiosperms (without considering *A. trichopoda*), as well as in *F. trinervia*. Having more gene copies can free up one copy to acquire new regulatory or catalytic properties required for C_4_ photosynthesis without jeopardizing its native role in C_3_ plants. In fact, gene duplication and neofunctionalization have been recognized as major factors contributing to evolution of C_4_ photosynthesis (Monson, 2003; Emms et al., 2016).

*Protein properties*: though PPT1 and PPT2 are functionally redundant in terms of their role of transporting PEP in *A. thaliana*, PPT1 shows lower specificity to PEP and higher permeability to 2-phosphoglycerate, i.e., there are differences in protein properties between PPT1 and PPT2 (Knappe et al., 2003). Here we observed more amino acid changes in PPT1 than PPT2 during evolution in *Flaveria* (**Figure 6**). We also found a larger insertion with either four or five repeated 13-aa elements in C_4_-like and C_4_ species in *Flaveria* clade A species, but this element was not observed in PPT1 from other C_4_ species, such as *G. gynandra* and *Z. mays* (**Figure S5**). Accumulation of new mutations may contribute to the suitability of PPT1 for C_4_ photosynthesis. The observation that PPT1 from either C_3_ or C_4_ *Flaveria* rescued the reticulate leaf phenotype of *cue1* showed that the PEP transport function of PPT1 was not altered between C_3_ and C_4_ plants (**Figure 7**). In the future, more detailed functional studies of catalytic properties of PPT1 from different C_3_ and C_4_ species are needed to test whether the acquired amino acid modifications in PPT1 have particular sequence variations that make it more suitable to function in a C_4_ context, e.g., increased specificity or transport rate.

### Mechanisms Underlying Establishment of New Expression Patterns of PPT1

*Gaining new cis-elements*: the function of C_4_ photosynthesis requires high expression of required genes upon light induction in specific cell types (Sheen, 1999). Reports show that the expression levels of C_4_ related genes are usually up-regulated upon light induction in C_4_ plants, while they are not necessarily up-regulated in C_3_ plants, suggesting that the mechanisms controlling the light-induced of expression of C_4_ related genes were acquired during C_4_ evolution (Christin et al., 2013; Xu et al., 2016). Here we showed that, during C_4_ evolution, PPT1 shifted its expression from predominantly in root to in leaf, and from predominantly in BSC to in MC (**Figures 3B, C**). We also found that PPT1 gained faster and long-lasting light induction during evolution (**Figure 4**). Moreover, we found that PPT1 gained a MEM1 B submodule (**Figure 5**) in its promoter region, which controls the high transcript abundance of *PEPCA* and *CA3* in MC in C_4_ *Flaveria* species (Akyildiz et al., 2007; Gowik et al., 2017). Notably, a similar MEM1 B submodule was also identified at 1,500 bp upstream of the start codon of PPDK (**Supplemental File 3**).

Considering that PPDK catalyzes generation of PEP, while PEPC uses PEP as its substrate, possessing a common *cis-*element in PPT1, PEPC, and PPDK enables coordinated up-regulation of expression of these proteins for efficient C_4_ photosynthesis. This re-utilization of shared *cis-*regulatory elements can be realized through transposon-mediated movement of *cis-*elements between genes (Cao et al., 2016). Interestingly, in an earlier study of the origin of crassulacean acid metabolism (CAM), Zhang et al. (2016) showed that the expression levels of PEPC, PEPC kinase, and PPDK are strongly co-regulated during day and night, and thus might have gained common transcriptional regulatory mechanisms enabling them to be co-recruited to support CAM. Therefore, co-recruiting common *cis*-regulatory elements might have played critical roles during the evolution of both C_4_ and CAM.

**Figure 8:**
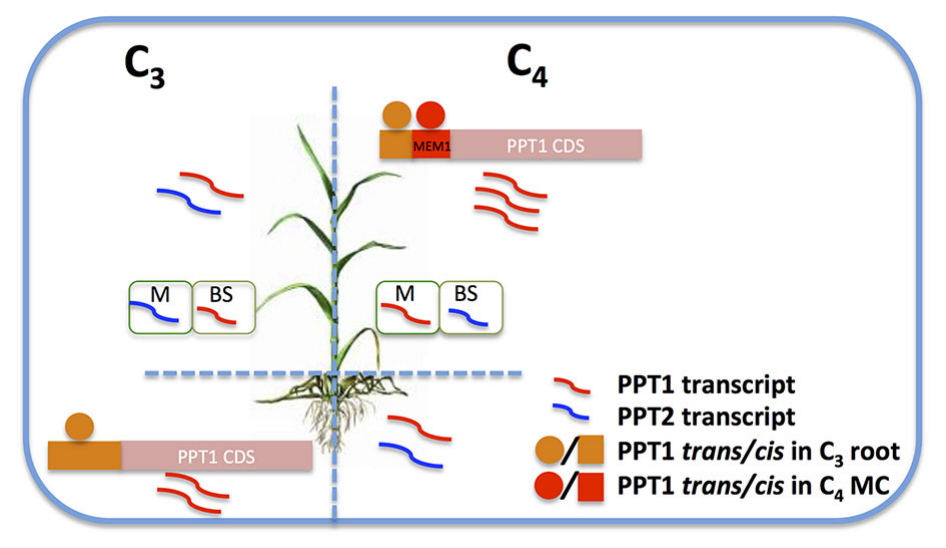
A hypothesis of the increased transcript abundance of PPT1 in C_4_ leaf MC. In C_3_ species, PPT1 (red curved line) shows predominant expression in root and leaf BSC, while PPT2 (blue curved line) predominates in leaf MC. PPT1 was recruited to C_4_ photosynthesis by gaining a dramatic increase of the transcript level in leaf MC, resulting in a switched transcript pattern compared to C_3_ species in terms of leaf and root, MC and BSC. The enhanced transcript abundance of PPT1 in C_4_ leaf MC may be a result of using *trans*-elements (red circle) in C_4_ MC by recruiting new *cis*-elements (red rectangle) such as MEM1 B submodule, or/and using the original regulatory mechanisms presented in C_3_ root (orange circle and rectangle). (Abbreviations, MC, mesophyll cell; BSC, bundle sheath cell.)

*Using root regulatory mechanisms*: PPT1 showed higher transcript abundance in root in C_3_ species, and it is possible that the increased transcript abundance of PPT1 in leaf may be a result of using pre-presenting regulatory mechanisms in root, including both *cis*- and *trans*-elements, as illustrated in **Figure 8**. Kulahoglu et al. (2014) showed that a module in root includes genes with high transcript abundance in *T. hassleriana*, a C_3_ species in the Cleomaceae family, while at the same time they show high transcript abundance in leaves of the C_4_ species *G. gynandra*, which is from the same family (Kulahoglu et al., 2014). This root module was recruited to support C_4_ with carbonic anhydrase and DIC1. The same scenario might underlie the increased transcript abundance of PPT1 in C_4_ leaf.

## Data Availability Statement

Publicly available datasets were analyzed in this study. This data can be found here: GSE54339 for Lyu et al. (2018), SRP036637 and SRP036837 for Kulahoglu et al., BioProject PRJNA395007 for Moreno-Villena et al. 2018, SRA066236 for Aubry et al. (2014), SRA012297 for Li et al. (2010), GSE54272 for Tausta et al. (2014), SRP052802 for Denton et al. (2017), SRP009063 for Chang et al. (2012), PRJEB5074 for John et al. (2014), and SRX1160366 for Rao et al. (2016).

## Author Contributions

X-GZ, GC and M-JL designed the project and wrote the paper. M-JL did bioinformatics analysis and qRT-PCR. YW, JJ, and XL conducted the transgenic experiments.

## Funding

This work was sponsored by Shanghai Sailing Program (17YF421900), Strategic Priority Research Program of the Chinese Academy of Sciences (Grant No. XDB27020105), National Science Foundation of China (31870214, 31701139, 31500988), National Research and Development Program of Ministry of Science and Technology of China (2019YFA0904600).

## Conflict of Interest

The authors declare that the research was conducted in the absence of any commercial or financial relationships that could be construed as a potential conflict of interest.
