# Supplementary material for: What Matters for C4 Transporters: Evolutionary Changes of Phosphoenolpyruvate Transporter for C4 Photosynthesis
Source: Front Plant Sci. 2020 Jun 30;11:935. doi: 10.3389/fpls.2020.00935 (PMC7338763; doi:10.3389/fpls.2020.00935)
Supplement: Supplementary file 1 [file DataSheet_1.docx]

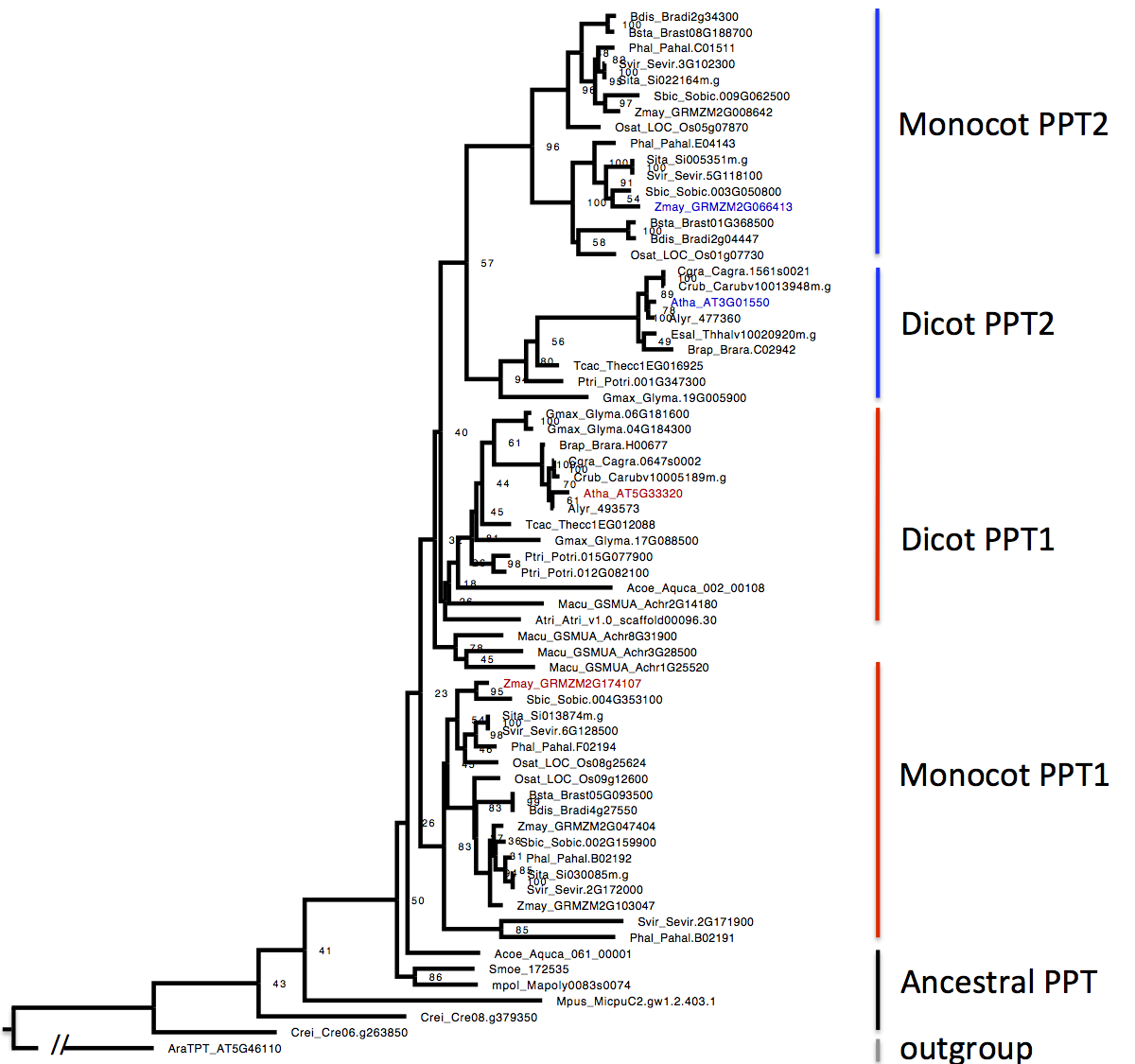


**Figure S1. Gene tree of PPT family from 23 representative species of Viridiplantae**

The tree was inferred from the alignment of protein sequences of PPTs based on maximum likelihood method. The numbers besides each node are the bootstrap scores from 1000 bootstrap samplings. PPT1 of *A. thaliana* and *Z. may*s are highlighted in red and PPT2 in blue. Triose phosphate/phosphate translocator (TPT) of *A. thaliana* is used as an outgroup.


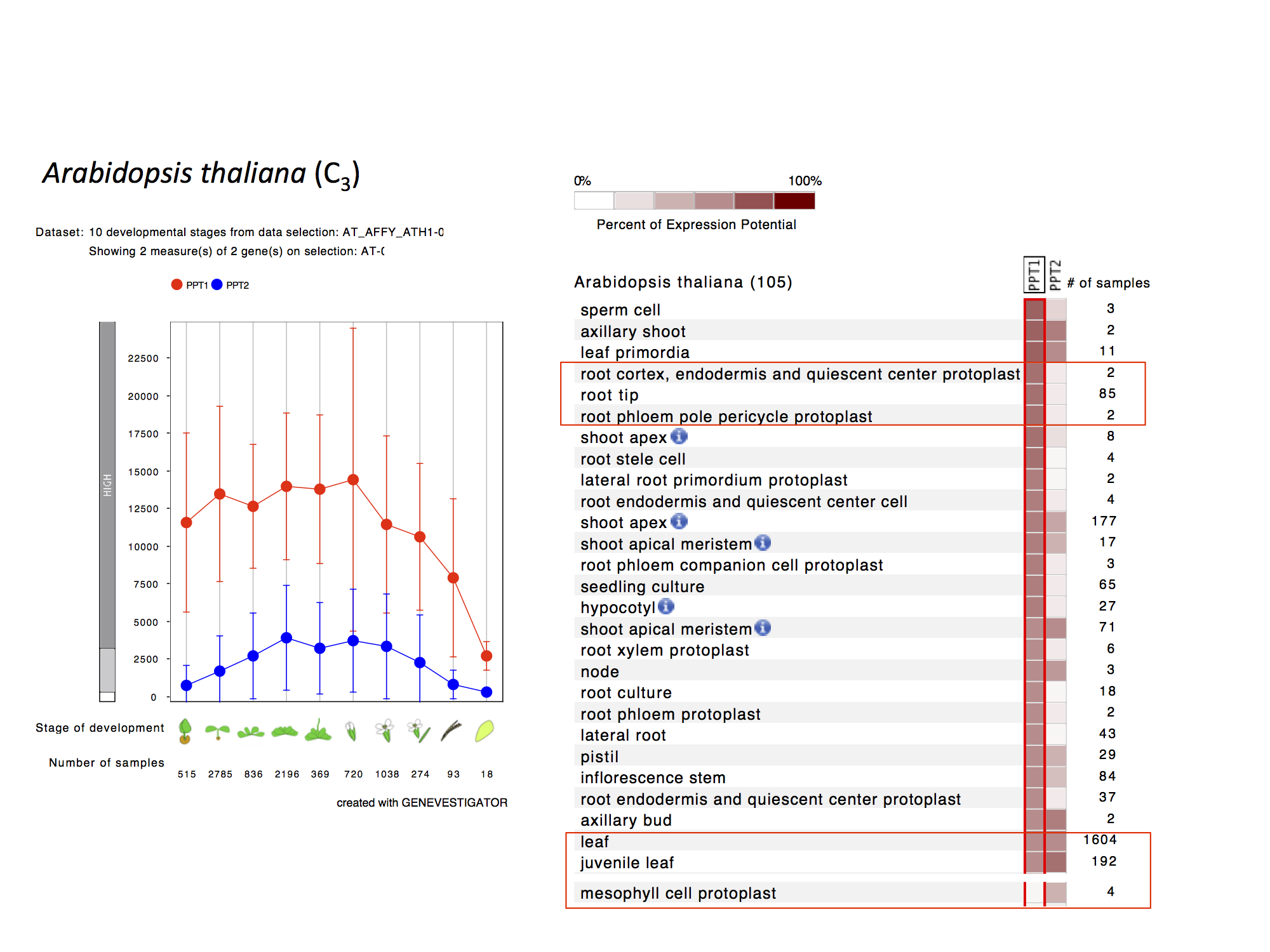


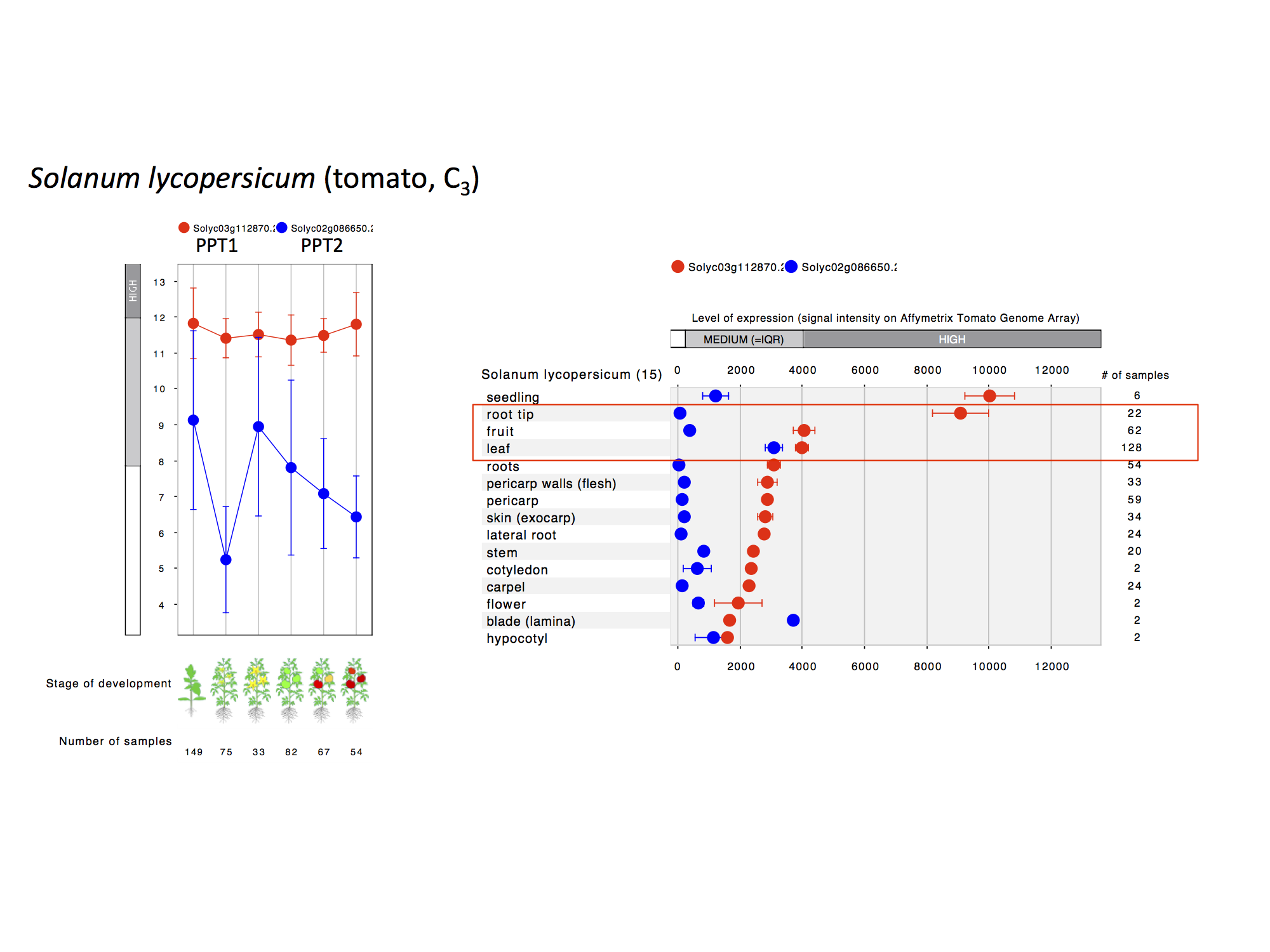


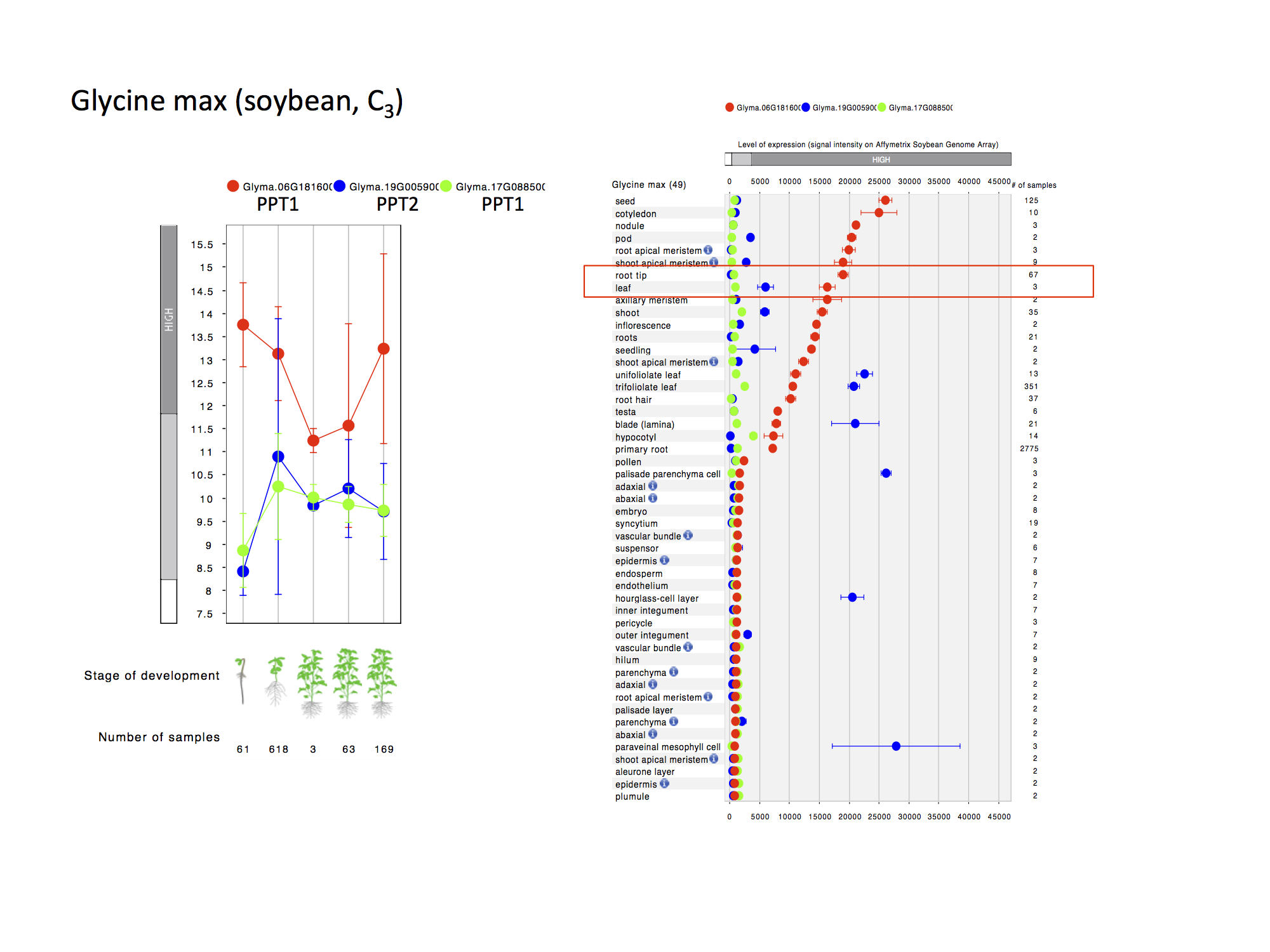


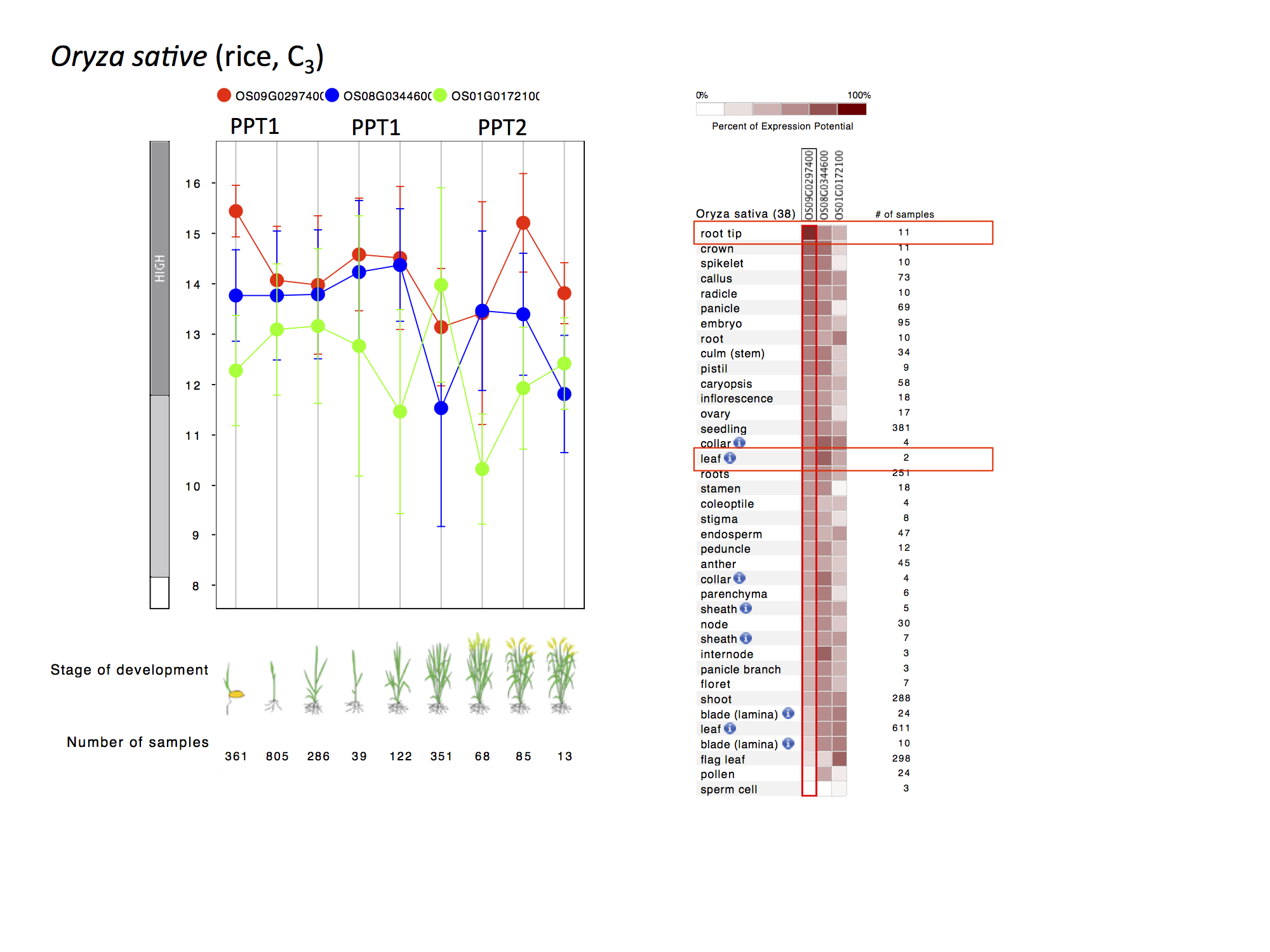


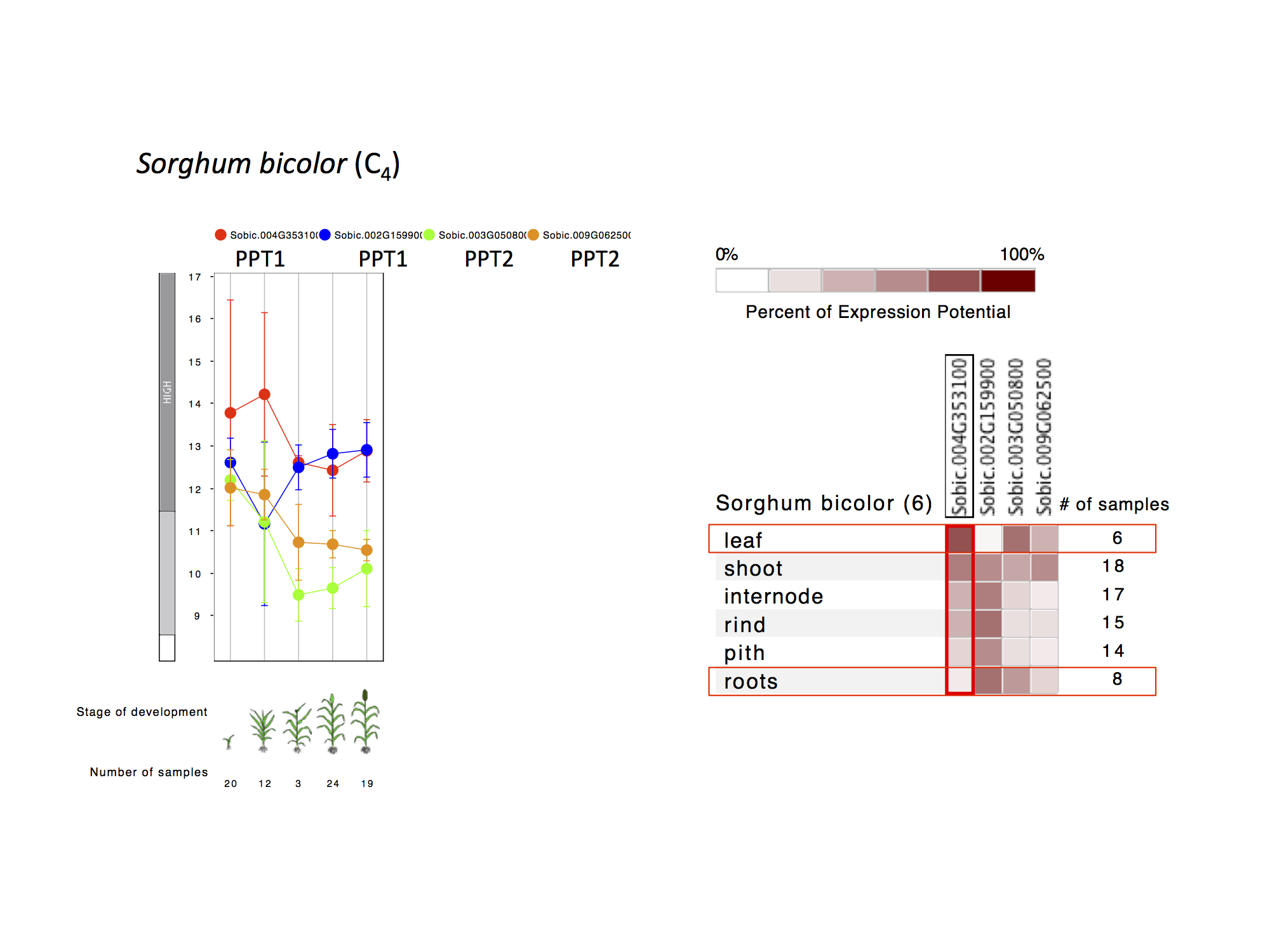


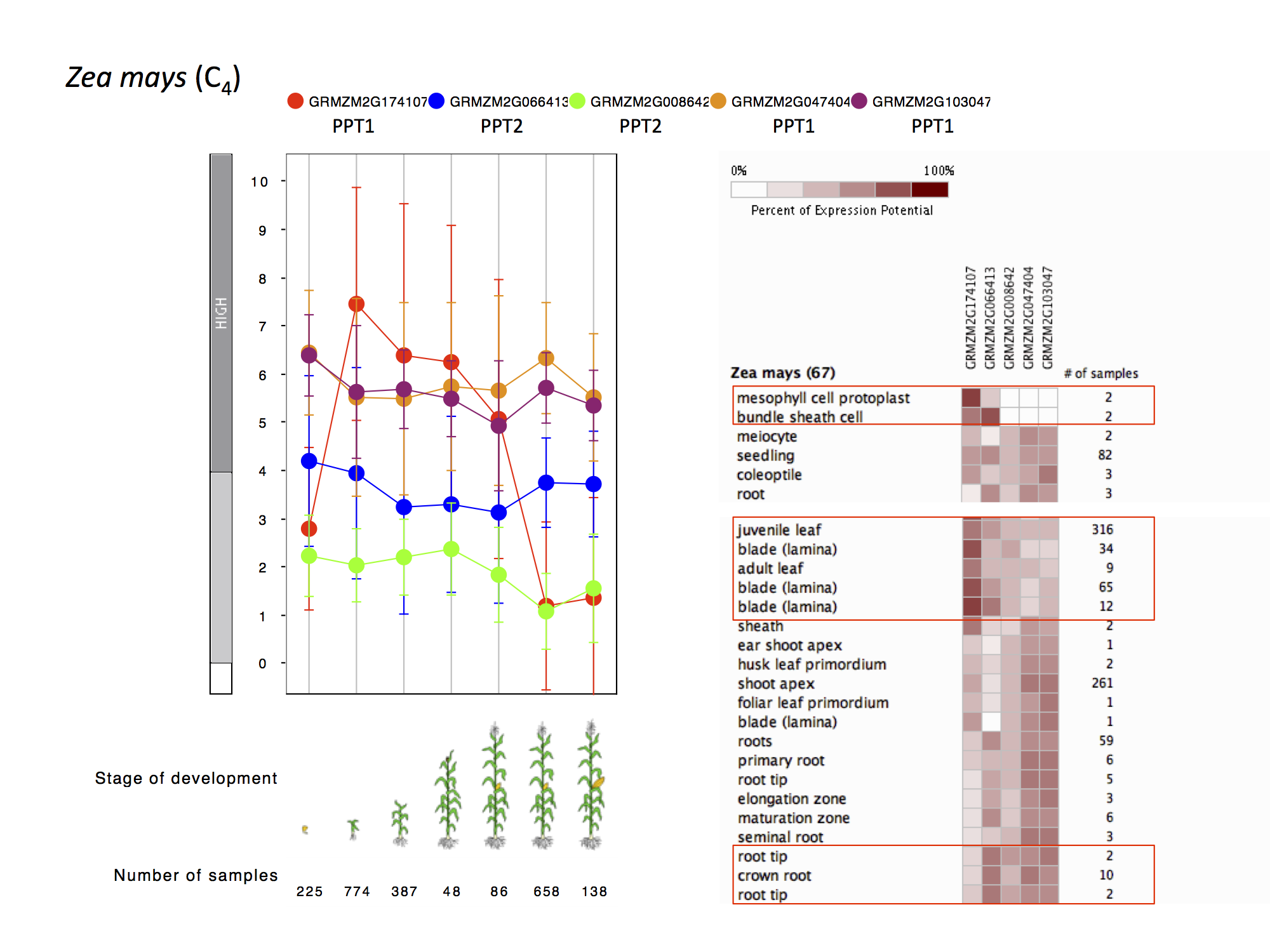

**Figure S2. The expression pattern of PPTs in C_3_ and C_4_ species based on GENEVESTIGATOR database**

We examined the expression patterns of PPT1 and PPT2 in four C_3_ and two C_4_ species on the scales of development stages and cell types. The species were labelled on the left top of each figure. For each species, the left panel shows the expression patterns of PPT1 and PPT2 on developmental scale, in which the average was calculated from samples at the same development stage regardless of tissue type and cell type. And the right panel shows the expression patterns of the PPT1 and PPT2 in different organs and cells types, which was arranged as the same order of that in the figure of development. The expression patterns of PPT1 and PPT2 were summarized in the table. Filled colors represent: red: PPT1 dominant; blue: PPT2 dominant; orange: PPT1 and PPT2 showing comparable expression level; white: data not available.

**Figure S3. Two amino acid showing signal of positive selection in the amino acid sequences of PPT2 fron C_4_ species**

Figure shows the alignment of protein sequences of PPT2 from 12 species that have full *de novo* assembled PPT2 coding sequences. Open reading frame and protein sequences were predicted based assembled transcript. Positive selection was performed using CDS branch-site model in PAML package based on the alignment of coding sequence and phylogeny of the 12 species. The phylogeny of the 12 species was subtracted from the phylogenetic tree of *Flaveria* species in our previous work (Lyu et al, 2015). The predicted two positive selected sites in C_4_ species of PPT2 were marked in red frame.

**Figure S4. The insertion of 13-aa element in the protein sequences of PPT1 from C_4_-like and C_4_ species in clade A**

(A) Amplified PPT1 based on PCR from juvenile and mature leaves of C_4_ and C_4_-like species.

(B) The alignment of PPT1 coding sequences from different *Flaveria* species. The number of 13-aa copies were confirmed by Sanger sequencing.


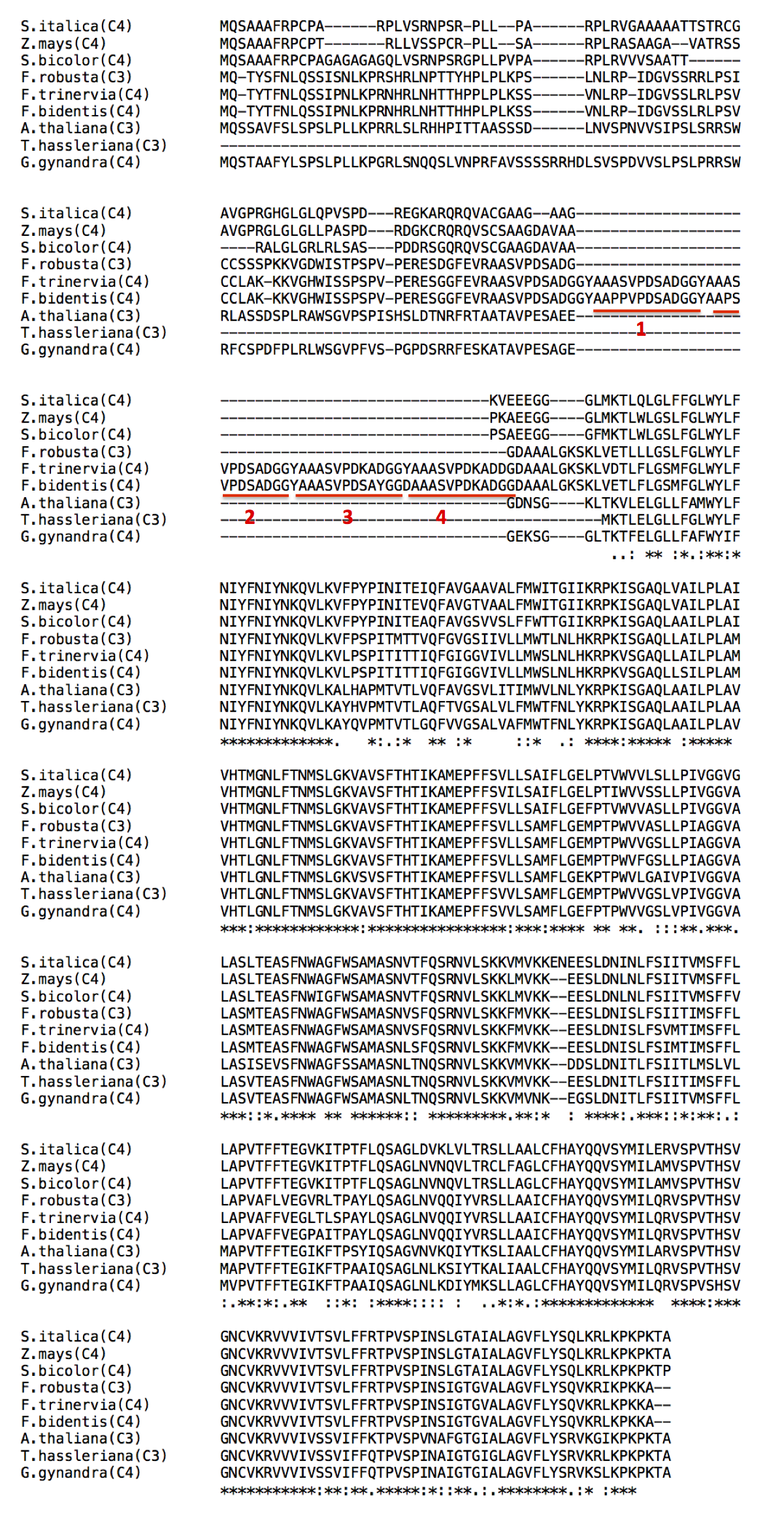


**Figure S5. The 13-aa element insertion is not universal.**

Figure shows the alignment of PPT1 amino acid sequences from different species, including three C_3_ species, namely, *A. thaliana*, *T. hassleriana* and *F. robusta*, and six C_4_ species, including *F. bidentis*, *F. trinervia, G. gynandra*, *S. biocolor*, *Z. mays* and *S. italica*. The alignment shows that the 13-aa-element insertion is only presents in the *Flaveria* C_4_ species.


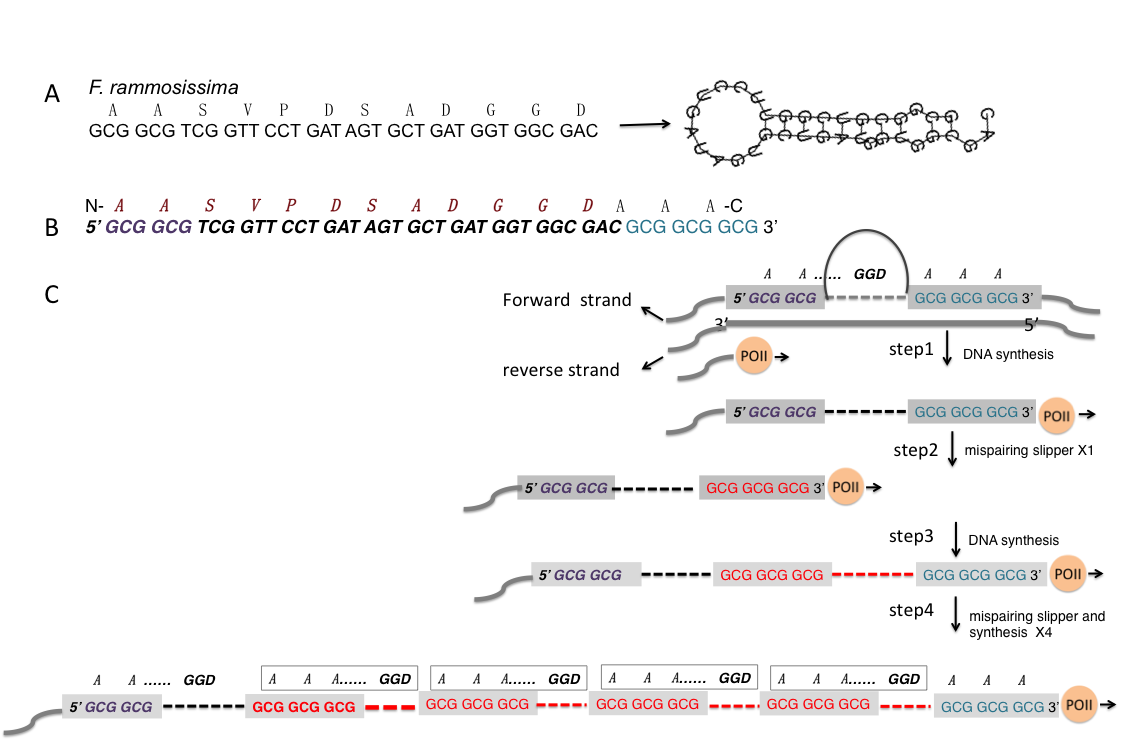


**Figure S6. A proposed slipping mispairing mechanism explaining the 4x13-aa insertion of PPT1 in *Flaveria*** C_4_ **species**

(A) The 12-aa sequence that exists in *F. ramosissima*, which is similar to the 13-aa segment but missing one Alanine at the N-terminal. The DNA that encodes the 12 amino acids is predicted to form a stable hairpin structure, which shortens the distance between the “GCGGCG” at 5’ end and “GCGGCGGCG” at 3’ end.

(B) There is a triplet Alanine encoded by “GCGGCGGCG” (in blue) following the C-terminal end of the 12-aa segment in *F. ramosissima*.

(C) The 4*13-aa insertion might be generated by a misparing mechanism during DNA synthesis. When Polymerase II leads DNA synthesis to the 3’GCGGCGGCG labelled in blue (step1), it slips back to the 5’GCGGCG labeled in purple (step 2), and re-synthesize the nucleotides between 5’GCGGCG and 3’ GCGGCGGCG, which results in an extra 13-aa sequence with a triplet Alanine encoded by “GCGGCGGCG” at 5’ end labelled in red (step3). Four copies of 13-aa segment are generated as a result of four times of slipping back.

**Figure S7. Subcellular localization of PPT1 and PPT2 in *Flaveria* species**

(A) GFP-fused PPT1 CDS from *Flaveria* species were transiently transform into wild tobacco. Green fluorescence is given from GFP, and red color is the fluoresce given by chloroplast. (B) GFP-fused PPT1 CDS from *F. bidentis* by removing the four 13-aa-element insertion sequence transiently transformed into wild tobacco.

**Table S1. Abbreviations of species used in this study**

| Abbr. | Species | PS. Type^#^ | Abbr. | Species | PS. Type^#^ |
| --- | --- | --- | --- | --- | --- |
| Hcal | *Heliotropium calcicola* | **C_3_** | **Aziz** | *Acroceras zizanioides* | **C_3_** |
| Hkar | *Heliotropium karwinsky* | **C_3_** | **Clat** | *Chasmanthium latifolium* | **C_3_** |
| Hfif | *Heliotropium filiforme* | **C_3_-C_4_** | **Cpat** | *Cyrtococcum patens* | **C_3_** |
| Hgre | *Heliotropium greggii* | **C_3_-C_4_** | **Dcal** | *Danthonia californica* | **C_3_** |
| Hrac | *Heliotropium racemosum* | **C_3_-C_4_** | **Hamp** | *Hymenachne amplexicaulis* | **C_3_** |
| Hten | *Heliotropium tenuifolium* | **C_4_** | **Hpro** | *Homopholis proluta* | **C_3_** |
| Htex | *Heliotropium texanum* | **C_4_** | **Lsor** | *Lasiacis sorghoidea* | **C_3_** |
| Mpen | *Mollugo pentaphylla* | **C_3_-C_4_** | **Ossp** | *Steinchisma* sp. | **C_3_** |
| Mver | *Mollugo verticillata* | **C_3_-C_4_** | **Pssp** | *Poa* sp. | **C_3_** |
| Mcer | *Mollugo cerviana* | **C_4_** | **Ppyg** | *Panicum pygmaeum* | **C_3_** |
| Nann | *Neurachne annulari* | **C_3_** | **Sstr** | *Sacciolepis striata* | **C_3_** |
| Nlan | *Neurachne lanigera* | **C_3_** | **Asem** | *Alloteropsis semialata* | **C_4_** |
| Nten | *Neurachne tenuifolia* | **C_3_** | **Daeg** | *Dactyloctenium aegyptium* | **C_4_** |
| Nmin | *Neurachne minor* | **C_3_-C_4_** | **Dcil** | *Digitaria ciliaris* | **C_4_** |
| Nmun | *Neurachne munroi* | **C_4_** | **Esta** | *Echinochloa stagnina* | **C_4_** |
| Fcro | *Flaveria cronquistii* | **C_3_** | **Pfim** | *Paspalum fimbriatum* | **C_4_** |
| Frob | *Flaveria robusta* | **C_3_** | **Pque** | *Panicum queenslandicum* | **C_4_** |
| Fson | *Flaveria sonorensis* | **C_3_-C_4_** | **Sbar** | *Setaria barbata* | **C_4_** |
| Fang | *Flaveria angustifolia* | **C_3_-C_4_** | **Shir** | *Stipagrostis hirtigluma* | **C_4_** |
| Fano | *Flaveria anomala* | **C_3_-C_4_** | **Chas** | *Cleome hassleriana* | **C_3_** |
| Fpub | *Flaveria pubescense* | **C_3_-C_4_** | **Cgyn** | *Cleome gynandra* | **C_4_** |
| Fchl | *Flaveria chlorofolea* | **C_3_-C_4_** |  |  |  |
| Fflo | *Flaveria floridana* | **C_3_-C_4_** |  |  |  |
| Fram | *Flaveria ramosisima* | **C_3_-C_4_** |  |  |  |
| Fbro | *Flaveria brownii* | **C_4_-like** |  |  |  |
| Fpal | *Flaveria palmeri* | **C_4_-like** |  |  |  |
| Fvag | *Flaveria vaginata* | **C_4_-like** |  |  |  |
| Fkoc | *Flaveria kochiana* | **C_4_** |  |  |  |
| Fbid | *Flaveria bidentis* | **C_4_** |  |  |  |
| Ftri | *Flaveria trinervia* | **C_4_** |  |  |  |
| Faus | *Flaveria australasica* | **C_4_** |  |  |  |

^#^: Photosynthetic type.

**Table S2. Primers used in this study**

| **qRT-PCR** | |
| --- | --- |
| ACTIN7-F | GTGCTGGATTCTGGAGATGGTG |
| ACTIN-R | TTCGGCGGTGGTGGTGA |
| Frobusta-PPT1-F | TAGTGGAAGGAGTCAAATTTACCCC |
| Frobusta-PPT1-R | CACGCTTCACACAGTTACCCACA |
| Fsonorensis-PPT1-F | AGTCAAATTTACCCCTGCATACCTG |
| Fsonorensis-PPT1-R | ATGCTAAAATTACCACCACACGCTTC |
| Framosissima-PPT1-F | GGTTGCTTCGCTTTTACCAATTGCT |
| Framosissima-PPT1-R | TTCTTGCTGAGAACATTACGCGACT |
| Ftrinervia_PPT1_F | TGACTCTTAGCCCTGCATACCTC |
| Ftrinervia_PPT1_R | ATATAGGAAACCTGCTGATAGGCAT |
| Faustralasica-PPT1-F | TGACTCTTAGCCCTGCATACCTCC |
| Faustralasica-PPT1-R | ACCACACGTTTCACACAGTTACCAA |
| Frobusta-PPT2-F | CCTTTACTGAGGCTTCATTCAACTGG |
| Frobusta-PPT2-R | AAGGACAAGATCGTCATAACCGAGA |
| Fsonorensis-PPT2-F | CAATGGCTGCAAATGTCACGAAC |
| Fsonorensis-PPT2-R | AAAGACAAGATCGTCATAACCGAGA |
| Framosissima-PPT2-F | CTTCACTGAGGCTTCATTTAACTGG |
| Framosissima-PPT2-R | AAAGACAAGATCGTCATAACCGAGA |
| Ftrinervia_PPT2_F | TGGCTGCAAATGTCACGAAC |
| Ftrinervia_PPT2_R | ACAAGATCGTCATAACCGAGA |
| Faustralasica-PPT2-F | CAATGGCTGCAAATGTCACGAAC |
| Faustralasica-PPT2-R | AAAGACAAGATCGTCATAACCGAGA |
| **Verify promoter sequence of PPT1 and PPT2 in four species** | |
| Frobusta_PPT1_P_F | GACATGAGAAACTCAAACAAA |
| Frobusta_PPT1_P_R | TATTCTTGGGATATCAGATTTGGC |
| Framosissima_PPT1_P_F | CATGTTTTGAGGATCGACCCA |
| Framosissima_PPT1_P_R | ATGTATATGTTTCTTCGAGGT |
| Ftrinervia_PPT1_P_F | CTCCCGATTTGTACACCATTACCAT |
| Ftrinervia_PPT1_P_R | TGGGGTTTTGCTTTGTTTTATATCGTT |
| Fsonorensis_PPT1_P_F | AAAGTTAGAATAGCCCTAAACAGC |
| Fsonorensis_PPT1_P_R | ATACCGTAAATGAAGGTGTCT |
| Frobusta_PPT2_P_F | TGATTACTCCCCATAGAACTAGCAT |
| Frobusta_PPT2_P_R | ATTTTGCTTGTGTATATGTGGCCTA |
| Framosissima_PPT2_P_F | GTGCATAGGCTCTATTTTAAGCTTC |
| Framosissima_PPT2_P_R | ATTTATGGCTGTCATTTAGTCCC |
| Ftrinervia_PPT2_P_F | ATCCCCATCAACAGAGATAGAGCTT |
| Ftrinervia_PPT2_P_R | ACATTTATGGCTGTCATTTGGTCCC |
| Fsonorensis_PPT2_P_F | CAATAAATAGTGTGCATCCGTTG |
| Fsonorensis_PPT2_P_R | ATTTATGGCTGTCATTTAGTCACC |
| **Check 13aa insertion on cDNA(for all species used)** | |
| PPT1-F | CCTATTGATGGTGTTTCGAGCCTT |
| PPT1-R | CTTGCTAAGAACATTACGCGACT |
| **Check 13aa insertion on DNA (for all species used )** | |
| PPT1-F | CTTAGGTTGCCTTCAGTATGCTGT |
| PPT1-R | CTTAGGTTGCCTTCAGTATGCTGT |
| **Genometyping** | |
| cue1-5-F | CCGCCGTATTCTCCCTCT |
| cue1-5-R | AGCGAACAACAAGCCAAG |
| Frobusta, Framosissima, Fbidentis, Fpalmeri-PPT1-F | GAGCTCGGTACCCGGGGATCCATGCAAACATACACCTTCAATCT |
| Frobusta, Framosissima, Fbidentis, Fpalmeri-PPT1-F | TTCTTCTCCTTTACTGccCCCGGGAGCCTTCTTAGGCTTTAAGCG |
| Fbidentis, Fpalmeri-PPT2-F | GAGCTCGGTACCCGGGGATCCATGGAGAGTTGTGCGTTAACG |
| Frobusta, Framosissima-PPT2-F | GAGCTCGGTACCCGGGGATCCATGGAGAGTTGTGCATTAACGAT |
| Frobusta, Framosissima, Fbidentis, Fpalmeri-PPT2-R | TTCTTCTCCTTTACTGccCCCGGGAGCAGCCTTTGGCTTTATCCG |
| ΔFbidentis-PPT-F | GCTGATGGTGGCGACGCGGCGGCGTTGG |
| ΔFbidentis-PPT1-R | CGCCGCCGCGTCGCCACCATCAGCACTGTC |
