# Supplementary material for: What Matters for C4 Transporters: Evolutionary Changes of Phosphoenolpyruvate Transporter for C4 Photosynthesis
Source: Front Plant Sci. 2020 Jun 30;11:935. doi: 10.3389/fpls.2020.00935 (PMC7338763; doi:10.3389/fpls.2020.00935)
Supplement: Supplementary file 2 [file DataSheet_1.pdf]

```
>AraTPT_AT5G46110
--MESRVLLRATANVVG-------------IPKLRRPIGAIHRQFS--------TASSSS
FSVKPIGGIGEGANLI----SGRQLRPILLLDSSAINGGEKREILKPVKAAAAEGGDTAG
DAKVGF-----------LAKYPWLVTGFFFFMWYFLNVIFNILNKKIYNY--FPYPYFVS
VIHLFVGVVYCLISWSVGLPK--RAPIDSNLLKVLIPVAVCHALGHVTSNVSFAAVAVSF
THTIKALEPFFNAAASQFIMGQSIPITLWLSLAPVVLGVAMASLTELSFNWLGFISAMIS
NISFTYRSIFSKKAMTD-----------MDSTNVYAYISIIALFVCIPPAIIVEG-----
-P----KLLNHGFADAIAKVGMTKFISDLFWVGMFYHLYNQLATNTLERVAPLTHAVGNV
LKRVFVIGFSIVIFGNKISTQTGIGTGIAIAGVAMYSIIKAKIEEEKRVTR--KESIESS
>Alyr_477360_380
--MFALTFITPNPRLPS---------------------------------------PLFV
AKSIPESA------------LSRRSIAFSSYHWRP----NLRFNGFKLKSATVPENVEGG
DSESGS-----------LVKGLKL--GGMFGVWYLLNIYYNIFNKQVLRV--YPYPATVT
AFQLGCGTLMIAVMWLLKLHP--RPKFAPSQFTAIVQLAAAHTLGNLLTNVSLGRVNVSF
THTIKAMEPFFTVLLSVLLLGEWPSLWTVCSLLPIVAGVSLASFTEASFNWIGFCSAMAS
NVTNQSRNVLSKKFMVGKE--------AMDNINLFSVITIISFISLVPVAILIDG--FKL
TP----WDLQIATSQGLS---VKEFCIMSLLAGVCLHSYQQVSYMILEMVSPVTHSVGNC
VKRVVVITSSILFFKTPVSPLNSIGTATALAGVYLYSRAKRV-----KVKQNPKTS----
>Alyr_493573_406
--MQSSAVFSLSPSLPL---------LKPRRLSLRHHSLTTAS-----------SDLNIS
PNVVSIPSLSRRSWRL----ASSDSPLRAWSGVPSSISHSLDTNRFKTAATAVPESAEEG
DNSGK------------LTKILEL--GLLFAMWYLFNIYFNIYNKQVLKA--LHAPMTVT
LVQFAVGSVLITIMWVLNLYK--RPKISGAQLAAILPLAVVHTLGNLFTNMSLGKVSVSF
THTIKAMEPFFSVLLSAMFLGETPTPWVLGAIVPIVGGVALASISEVSFNWAGFLSAMAS
NLTNQSRNVLSKKVMVKKD--D-----SLDNITLFSIITLMSLVLMAPVTFFTEG--IKF
TP----SYIQSA---GVN---VQQIYTKSLIAALCFHAYQQVSYMILARVSPVTHSVGNC
VKRVVVIVSSVIFFKTPVSPVNAFGTGIALAGVFLYSRVKGI-----KPKP--KTA----
>Atha_AT3G01550.1_383
--MFALTFLNPNPRLPS---------------------------------------PLFL
AKSTPESALSR---------RSRAFSSSNSYPWRP----NLRFNGFKLKSATVPENVEGG
DLESGS-----------LVKGLKL--GGMFGVWYLLNIYYNIFNKQVLRV--YPYPATVT
AFQLGCGTLMIAIMWLLKLHP--RPKFSPSQFTVIVQLAVAHTLGNLLTNVSLGRVNVSF
THTIKAMEPFFTVLLSVLLLGEWPSLWIVCSLLPIVAGVSLASFTEASFNWIGFCSAMAS
NVTNQSRNVLSKKFMVGKD--------ALDNINLFSIITIISFILLVPLAILIDG--FKV
TP----SHLQVATSQGLS---VKEFCIMSLLAGVCLHSYQQVSYMILEMVSPVTHSVGNC
VKRVVVITSSILFFKTPVSPLNSIGTATALAGVYLYSRAKRV-----QVKPNPKMS----
>Atha_AT5G33320.1_408
--MQSSAVFSLSPSLPL--------LKPRRLSLRHHPITTAASS----------SDLNVS
PNVVSIPSLSRRSWRL----ASSDSPLRAWSGVPSPISHSLDTNRFRTAATAVPESAEEG
DNSGK------------LTKVLEL--GLLFAMWYLFNIYFNIYNKQVLKA--LHAPMTVT
LVQFAVGSVLITIMWVLNLYK--RPKISGAQLAAILPLAVVHTLGNLFTNMSLGKVSVSF
THTIKAMEPFFSVLLSAMFLGEKPTPWVLGAIVPIVGGVALASISEVSFNWAGFSSAMAS
NLTNQSRNVLSKKVMVKKD--D-----SLDNITLFSIITLMSLVLMAPVTFFTEG--IKF
TP----SYIQSA---GVN---VKQIYTKSLIAALCFHAYQQVSYMILARVSPVTHSVGNC
VKRVVVIVSSVIFFKTPVSPVNAFGTGIALAGVFLYSRVKGI-----KPKP--KTA----
>Cgra_Cagra.1561s0021.1_381
--MFPLTFVTSNPRLPS-----------------------------------------PF
FVVKSTA-------------LSRRSIPFSSSTSFPWRPNLYRSNGFKLKSATAPEDVDGG
DSKSAS-----------LVKGLKL--GGMFGVWYLLNIYYNIFNKQVLRV--YPYPATVT
AFQLGCGTLMIAIMWLLKLHP--RPKFAPSQFTAIAQLAAAHTLGNLLTNVSLGRVNVSF
THTIKAMEPFFTVLFSVLLLGEWPSLWIVCSLLPIVAGVSLASFTEASFNWIGFCSAMAS
NVTNQSRNVLSKKFMVGKE--------ALDNINLFSVITIISFVLLVPVAIIMDG--FKL
TP----SQLQIATSQGVS---VKEFCVMSLLAGVCLHSYQQVSYMILEMVSPVTHSVGNC
VKRVVVITSSIMFFQTPVSPLNAIGTATALAGVYLYSRAKRV-----KVKPNPKGC----
>Cgra_Cagra.0647s0002.1_413
--MQSSAVFSLSPSLPL---------LKPRRLSLRHPLTTAASSSSSSNL----NDLNVS
PNVVSIPSLSRRSWRL----ASSDSPLRAWSGVPSSISHSFDSNRFKTAATAVPESADDG
EKSGK------------LTKMLEL--GLLFAMWYLFNIYFNIYNKQVLKA--LHAPMTVT
LVQFAVGSVLITVMWALNLYK--RPKISGAQLAAILPLAVVHTLGNLFTNMSLGKVSVSF
THTIKAMEPFFSVLLSAMFLGEAPTPWVIGAIIPIVGGVALASISEVSFNWAGFTSAMAS
NLTNQSRNVLSKKVMVKKD--D-----SLDNITLFSIITLMSLVLMAPVTFFSEG--IKF
TP----SYIQSV---GVN---VQQIYTKSLIAALCFHAYQQVSYMILARVSPVTHSVGNC
VKRVVVIVSSVIFFKTPVSPVNAFGTGIALAGVFLYSRVKGI-----KPKP--KTA----
>Crub_Carubv10013948m_381
--MFPLTFVTSNPRLPS-----------------------------------------PF
FVVKSTA-------------LSRRSIPFSSSTSFPWRPNLYRSNGFKLKSATAPEDVDGG
DSKSES-----------LVKGLKL--GGMFGVWYLLNIYYNIFNKQVLRV--YPYPATVT
AFQLGCGTLMIAILWLLKLHP--RPKFAPSQFTAIAQLAAAHTLGNLLTNVSLGRVNVSF
THTIKAMEPFFTVLFSVLLLGEWPSLWIVCSLLPIVAGVSLASFTEASFNWIGFCSAMAS
NVTNQSRNVLSKKFMVGKE--------ALDNINLFSVITIISFVLLVPVAIIMDG--FKL
TP----SQLQIATSQGVS---VKEFCVMSLLAGVCLHSYQQVSYMILEMVSPVTHSVGNC
VKRVVVITSSIMFFQTPVSPLNAIGTATALAGVYLYSRAKRV-----KVKPNPKGC----
>Brap_Brara.C02942.1_383
--MLLITPY----------------------PRLVSPL--------------------LA
```

```
TKSTPESSFTRRARA-----SSSSSSSSSSSYHWPFLTPKRRLNGFKLKSATVPGDVESG
S----------------LVKGLKL--GGMFGVWYLLNIYYNIFNKQVLRV--FPYPATVT
AFQLGCGTLMISIMWLLKLHP--RPKVTPSQLPAILQLAAAHTLGNLLTNVSLGRVNVSF
THTIKAMEPFFTVLFSVLLLGEWPSLWIVCSLLPIVAGVSLASFTEASFNWIGFCSAMAS
NVTNQSRNVLSKKFMVEKE--------ALDNINLFSIITIISFVLLVPVAILIDG--FKF
TP----SHLQLATSQGLT---VKEFCLMSLLAGVCLHSYQQVSYMILEMVSPVTHSVGNC
VKRVVVIASSILFFKTPVSPLNSIGTATALAGVYLYTRAKRI-----KPNPNSKSS----
>Brap_Brara.H00677.1_407
--MQSSAVFSLSPSLPL---------LKPRRLSLRHPVTVTASS----------NLNVSP
PNVVSVPPLPRRSWRL----ASSDSPLRAWSGLPSVSSPSLDTNRFKTAATAVPENAEEG
EGSGK------------MTKVLEL--GLLFAMWYLFNIYFNIYNKQVLKA--LHAPMTVT
LVQFAVGSVLITFMWALNLYK--RPKISAAQLAAILPLAVVHTLGNLFTNMSLGKVSVSF
THTIKAMEPFFSVVLSAMFLGEVPTPWVIGSIIPIVGGVALASVTEVSFNWAGFLSAMAS
NLTNQSRNVLSKKVMVKKD--D-----SLDNITLFSIITLMSLFLMAPVTFFSEG--IKF
TP----SYIQSA---GVN---VQQIYTKSLIAALCFHAYQQVSYMILARVSPVTHSVGNC
VKRVVVIVSSVIFFKTPVSPVNAFGTGIALAGVFLYSRVKRI-----KPKP--KTA----
>Tcac_Thecc1EG016925t1_408
--MQSTA-LALSPTIPF-------FKPLNKNTNPRYSPLSNLSS----------LKPLDL
SSIHGLSCQKACY-------LSSWSSFNARISESFVVSNGRNDDSFKVRAASVPENKGET
KDSSG------------LSGTLQL--GAMFAIWYLLNIYFNISNKQVLKV--YTFPATVT
AFQLGCGTLMILIMWASNLYH--RPKLTRSQLAAIIPLAVIHTLGNLLTNVSLGQVNVSF
THTIKAMEPFFTVLFSVLLLGEWPTLWILSSLIPVVGGVALASFTEVSFNWIGFSSAMAS
NVTNQSRNVLSKKFMVRKE--D-----TLDNINLFSVITIISFILLVPTAILLEG--VKF
TP----SYLQSAANQGLN---VKELCIRSLLAGFCFHSYQQVSYMILQMVSPVTHSVGNC
VKRVVVIVSSVIFFQTPVSPINSLGTAVALAGVFLYSKAKRM-----KPKP--KAA----
>Esal_Thhalv10020920m_382
--ML----LTPYPRLPS---------------------------------------PLLA
AKSNPESSLTR---------RARASYSTSNSYYWLFLRPNRRFNGFKLKSATVPENVEGD
VESGG------------LVKGLKL--GAMFGVWYLLNIYYNIFNKQVLRV--YPYPATVT
AFQFGCGTLMIAIMWLLKLHP--RPKIAPSQLPAIVQLAAAHTLGNFLTNVSLGRVNVSF
THTIKAMEPFFTVLFSVLLLGEWPSLWIVCSLLPIVAGVSLASFTEASFNWIGFCSAMAS
NVTNQSRNVLSKKFMVEKD--------ALDNINLFSVITIISFILLVPVAILIDG--FKF
TP----SHFQLATSQGLT---VKEFCFMSLLPGVCLHSYQQVSYMILEMVSPVTHSVGNC
VKRVVVIASSILFFQTPVSPLNSIGMATALAGVYLYTRAKRA-----KVKPNPKSC----
>Tcac_Thecc1EG012088t1_412
--MQSAA-FSLSSPSSF---------PSLKPRIFGSNLRFDPVRVSSSFSASKRHDLSAS
SNVVSVPSLPKRSWRLS---SSSGLPLRAWNSVPS----DSKAERFEVRATAAESAGEGE
KAGN-------------LMKTLEL--GLLFGLWYLFNIYFNIYNKQVLKV--FHYPVTVT
VIQFAVGTVLVALMWTFNLYK--RPKITGVQLAAILPLALVHTLGNLFTNMSLGKVAVSF
THTIKAMEPFFSVVLSAMFLGELPTVWVVGSLVPIVGGVALASVTEASFNWAGFWSAMAS
NLTNQSRNVLSKKVMVKKE--D-----AMDNITLFSIITVMSFILLAPAAIFMEG--VKF
TP----SYLQSA---GLN---VKEVVVRSLIAALCFHAYQQVSYMILQRVSPVTHSVGNC
VKRVVVIVSSVFFFKTPVSPINSLGTGIALAGVFLYSRVKRI-----KPKA--KAA----
>Ptri_Potri.001G347300.2_411
--MLRTA-LALSLSTPV--------------LRPQKPLYVHSSPRF--------SPCINL
PSLKPLDTVCCHGFSRRKT-SLSGCCLSCNTRISAFICRNHGYGSIKVRAASVPDSTGEF
EKSSD------------AARTMQL--GAMFGIWYLLNIYFNIFNKQVLKV--YPFPATIT
AFQVGCGTVMIIIMWALNLCN--RPKLTRPQILAILPLAVAHTFGNLLTNVSLGKVAVSF
THTIKALEPFFTVLFAALFLGETPAFWVLSSLVPLVGGVGLASLTEVSFNWIGFCSAMAS
NVTNQSRNVFSKKLMVNKE--E-----TLDNVNLFSVITIISFILLVPAAIFMEG--FKF
TP----SYLQSAANQGLN---VKELCIRSLLAGFCFHSYQQVSYMILQMVDPVTHAVGNC
VKRVVVIVSSVIFFQTPVSPINSIGTAMALAGVFLYSRAKRV-----KSKTKPKAA----
>Ptri_Potri.012G082100.1_414
--MQSTA-FTPSSSSSL---------SPLKPRRLVANPIYSLPSRFDPIRAFSSSSSKRH
DPDSNNVVFPRRSWS-----LSSASNSSLSRPWNPLVS-ERKMERFEVKATAVPESAGEG
KEKSS------------LTKTLEL--GLLFGLWYLFNIYFNIYNKQVLRV--FPNPVTIT
AAQFTVGTVLVACMWTFNLYK--KPKVSGAQLAAILPLAVVHTLGNLFTNMSLGKVAVSF
THTIKAMEPFFSVVLSAMFLGEMPTLWVVGSIIPIVGGVALASVTEASFNWAGFWSAMAS
NLTNQSRNVLSKKVMLKKE--E-----SMDNITLFSIITIMSFILLAPVTIFMEG--VKF
TP----AYLQSV---GLN---VKEVYTRAFLAALCFHAYQQVSYMILQRVSPVTHSVGNC
VKRVVVIVSSVLFFKTPVSPINSLGTGIALAGVFLYSRVKSI-----KPKP--KTA----
>Ptri_Potri.015G077900.1_416
--MQSTA-FTFSPSPSL--------LKPRRLISSSSTATYSLPPRFDPIRAFSSSKRYDL
DSNNVVFPRRSWSLSS----ASNSSLSRPWNPLPPLVS-ESKTERFEVRATAVPESAGEG
EEKSS------------LVKTLEL--GLLFGLWYLFNIYFNIYNKQVLKV--FPNPVTVT
AVQFAVGTVLVVFMWTFNLYK--KPKISGAQLAMILPLAVVHTLGNLFTNMSLGKVAVSF
THTIKAMEPFFSVVLSAMFLGEMPTLWVVGSLLPIVGGVALASVTEASFNWAGFWSAMAS
NLTNQSRNVLSKKVMVKNE--E-----SMDNITLFSIITIMSLVLLAPVTIFMEG--VKF
TP----AYLQSA---GLN---VKQVYTRSLIAALCFHAYQQVSYMILQRVSPVTHSVGNC
VKRVVVIVSSVFFFKTPVSPINSLGTGVALAGVFLYSRVKRI-----KPKP--KTA----
>Gmax_Glyma.19G005900.1_408
MSPYSLSLLASVSPKMH---------TLLSFSSSHSPHLPNHNITK--------SNNKLS
HSINGLSSC-----------PSSPLLPSATKSSPFLISTSKIASFRVLAASSIPDARSDE
PAKTSD-----------FLKTLQL--GAMFATWYLLNIYYNIYNKQVLKV--YPFPATIT
```

AFQFGFASLVINLVWTLNLHP--RPSISGSQFAAILPLAVAHTMGNLLTNISLGKVAVSF
THTIKAMEPFFTVVLSALLLGEMPTFWVVSSLVPVVGGVALASMTEVSFNWIGFTTAMAS
NVTNQSRNVLSKKLMTNEE--E-----TLDNINLYSVITIISFLLLVPCAILVEG--VKF
SP----SYLQSAASQGLN---VRELCVRSVLAAFCFHAYQQVSYMILQMVSPVTHSVGNC
VKRVVVIVSSVIFFQIPVSPVNTLGTGLALVGVFLYSRAKRI-----KSVQ--KTN----
>Gmax_Glyma.06G181600.1_406
--MQSAA-FTFSPSLPL---------RNPSPNSWRRPSLSL-------------RLHAKH
SNNNNNSNSTEGVNSNGVSSTSFTRRSWTLLPSSSFKFRPLPSSPPRAAENAVPESAAAP
VENP-------------LFKTLEL--GALFGLWYLFNIYFNIYNKQVLKA--FHYPVTVT
VVQFAVGTVLVAFMWGLNLYK--RPKLSGAMLGAILPLAAVHTLGNLFTNMSLGKVAVSF
THTIKAMEPFFSVILSAMFLGEFPTPWVVGSLVPIVGGVALASVTEASFNWAGFWSAMAS
NVTNQSRNVLSKKAMVKKE--D-----SMDNITLFSIITVMSFFLLAPVAIFMEG--VKF
TP----AYLQSA---GVN---VRQLYIRSLLAALCFHAYQQVSYMILQRVSPVTHSVGNC
VKRVVVIVSSVIFFQTPVSPVNAFGTAIALAGVFLYSRVKRI-----KAKP--KTA----
>Gmax_Glyma.04G184300.1_396
--MQSAA-FTFSPSLPL---------RNPSPNYWRRPSLSL-------------RLSAKH
GNSNSDDVNSNG--------VSSTFFTRRSWTLPPSSSFKFRPLPPRAAESAVPESAPVE
NP---------------LFKTLEL--GALFGLWYLFNIYFNIYNKQVLKA--FHYPVTVT
VVQFAVGTVLVAFMWGLNLYK--RPKLSGAMLGAILPLAAVHTLGNLFTNMSLGKVAVSF
THTIKAMEPFFSVVLSAMFLGEFPTPWVVGSLVPIVGGVALASVTEASFNWAGFWSAMAS
NVTNQSRNVLSKKAMVNKE--D-----SMDNITLFSIITVMSFFLLAPVAIFMEG--VKF
TP----AYLQSA---GVN---VRQLYIRSLLAALCFHAYQQVSYMILQRVSPVTHSVGNC
VKRVVVIVSSVIFFQTPVSPVNAFGTAIALAGVFLYSRVKRI-----KAKP--KTA----
>Gmax_Glyma.17G088500.1_382
--MQSTV-FSLSPSLSL----------------PKQPL----------------KRSVSV
STSKLNVILTP---------SSFPCQPCSLAYSPSFKLAPSHFHPFHARATSVPESSAGN
T----------------LLNTLEL--GALFGLWILFNIYFNIYNKQVLKV--YHFPLTVS
TLQFAVGSLFVAFMWSFNLYK--RPKVSGAQLAAILPLALVHTLGNLFTNMSLGQVAVSF
THTIKAMEPFFSVLLSAMFLGEAPTAWVVGSLVPIVGGVALASATEASFNWAGFWSAMAS
NLTNQSRNVLSKKVMVNKE--E-----SMDNITLFSIITVMSFLLSVPVTLLMEG--VKF
TP----AYLQSA---GLN---VNEVYIRSLLAALCFHAYQQVSYMILQKVSPVTHSVGNC
VKRVVVIVSSVIFFQTPVSPINALGTAIALAGVFLYSRVKRL-----KP----KTT----
>Acoe_Aquca_061_00001.1_408
--MSSTTAFTFSSTSSP--------FLKTRKTSFQNPN----------------PNCYRF
NPVRLSTTSKSLDLCLRS--SPLCSSSSRWSSIPSLVTDREMKNDFKTKASSSDGGASAD
EVETEKAGA--------LTKTLQL--GAYFGLWYLFNIYFNIYNKQVLKV--FPFPVTIT
TVQFAVGTVIVFFMWALNLHK--KPQITTSQLVAILPLAMVHTLGNLFTNMSLGKVAVSF
THTIKAMEPFFSVLFSALFMGEMPSLLVVSSLLPIVGGVALASMTEASFNWAGFWAAMAS
NVTFQSRNVLSKKFMVKKD--Q-----SMDNINLFSIITIMSLFLLFPVAFFMEG--VKV
SP----AYLQSA---GLN---VRDLGTKALLAGLCFHAYQQVSYMILQRVSPVTHSVGNC
VKRVVVIVTSVLFFRTPVSPINSLGTGIALAGVFLYSRIKSL-----KPKP--KAA----
>Acoe_Aquca_002_00108.1_405
--MEST--FALSPSIPF---------LKSRNPNLNLRFNYLNLSS---------NPSFNL
NSVNGVSSY-----------SSSSLQFSSWCYFPCIVNDRRRMEKFNVKATLASHDTNEE
EVERPSTTST-------LIKTLEL--GVLIGLWYILTIMFNIYNKQVLTV--YPFPLTIS
TLHFGMGTVIVLLMWTFNLHK--KPKISRSQLLAIIPLAIAHTLGNLFTNMSLGKVSVSF
THTIKAMEPFFSVVLSAMFLGEIPTFWVVSSLLPVVGGVALASFTEVSFNWIGFSTAMAS
NLTNQSRNVLSKKFMIKKEG-E-----YLDNVTLLSIITIMSFILMAPVSLLIEG--FRF
TP----AGLLSS---GLD---VKDIYVKTFVAGLFYHAYQQVAYIILERVSPVTHSVSNC
VKRIAIIVASVLFFQSPVSLINSFGTGVAIAGVFLYSRLKQV-----KSKP--KID----
>Sita_Si013874m_398
--MQSAAAFRPCPARPL------VSRNPSRPLLPARPL----------------RVGAAA
AATTSTRCGA----------VGPRGHGLGLQPVSPDREGKARQRQVACGAAGAAGKVEEE
GGG--------------LMKTLQL--GLFFGLWYLFNIYFNIYNKQVLKV--FPYPINIT
EIQFAVGAAVALFMWITGIIK--RPKISGAQLVAILPLAIVHTMGNLFTNMSLGKVAVSF
THTIKAMEPFFSVLLSAIFLGELPTVWVVLSLLPIVGGVGLASLTEASFNWAGFWSAMAS
NVTFQSRNVLSKKVMVKKENEE-----SLDNINLFSIITVMSFFLLAPVTFFTEG--VKI
TP----TFLQSA---GLD---VKLVLTRSLLAALCFHAYQQVSYMILERVSPVTHSVGNC
VKRVVVIVTSVLFFRTPVSPINSLGTAIALAGVFLYSQLKRL-----KPKP--KTA----
>Sita_Si030085m_396
--MQSAAAIGLVRPCAA--------RPLVACPSHRR------------------GGAVAG
EGIRPVLPQR----------GIRLSARAGLVPASPLEEEEKRRCRAERDVSAAAAAAQEA
GEEAGGG----------FAKTLQL--GALFGLWYLFNIYFNIYNKQVLKV--LPYPINIT
TVQFAVGTAIAMFMWITGILK--RPKISGAQLLAILPLAIVHTMGNLFTNMSLGKVAVSF
THTIKAMEPFFSVLLSAIFLGELPTPWVVLSLLPIVGGVALASLTEASFNWAGFWSAMAS
NVTFQSRNVLSKKLMVKKE--E-----SLDNINLFSIITVMSFCLLAPVTLLTEG--VKV
SP----AVLQSA---GLN---LKQIYTRSLIAAFCFHAYQQVSYMILARVSPVTHSVGNC
VKRVVVIVTSVLFFRTPVSPVNSLGTGVALAGVFLYSQLKRL-----KPKA--KAA----
>Sita_Si022164m_417
--MQSMAASCSSSSRAW-------AAARRSYPAPALPPSSHVAFSS--------SPPSTH
GCRWPVAGSGGPALPLGIRGGLRPLPSPLLPAGVGRAGAAARTRTAAAAAASLPAEDGGG
KPEGAAG----------ISRTLQL--GAMILVWYMLNIYFNIYNKLVLKA--VPFPYTIT
TFQFASGSFFITLMWLLNLHP--KPRLSLKQYAKILPLALIHMLGNVFTNLSLGKVAVSF
THTIKAMEPFFSVLLSVLFLGETPSLLVLGSLVPIVGGVLLASMTEVSFNWIGFWSAMAS

```
NLTNQSRNVFSKKLLADKE--D-----NLDDINLFSIMTIMAFLLSAPLMLSVEG--IKF
SP----SYLQSA---GVN---VKELCVKAALAGTCFHFYQQVSYSLLARVSPVTHSVTNS
LKRVVVIVSTVLFFRTPISPINALGTGVALAGVFLYSQFKKA-----KPKA--KAA----
>Phal_Pahal.F02194.1_403
--MQSAAAFRPCPARPL------VGRSPSRPLLPARPIRD--------------STGAGA
GAVAAAASTRCGA-------VGPRGLGHGLLPVSTDREGKARQRTVACAAAGKAGKAEEE
GGG--------------FLKTLQL--GAFFGLWYLFNIYFNIYNKQVLKV--FPYPINIT
EVQFAVGAAVAVFMWITGILK--RPKISGAQLLAIVPLAIVHTMGNLFTNMSLGKVAVSF
THTIKAMEPFFSVVLSAIFLGELPTVWVGLSLLPIVGGVALASLTEASFNWAGFWSAMAS
NVTFQSRNVLSKKLMVKKEDQE-----SLDNINLFSIITVMSFFLLAPVTFFTEG--VKM
TP----SFLQSA---GLN---VNQVLTRSLLAALCFHAYQQVSYMILAVVSPVTHSVGNC
VKRVVVIVTSVLFFRTPVSPINSLGTAIALAGVFLYSQLKRL-----KPKP--KTA----
>Phal_Pahal.B02192.1_398
--MQSAAAIGLVRPCAA--------RQLVACPSHRRGG----------------AVAVAG
GGIRPVLPLR----------GLRLSARAGLVPASPLEEEEKRRCRDVAASASAAAAAQGA
GEEAGGG----------LLKTLQL--GALFGLWYLFNIYFNIYNKQVLKV--LPYPINIT
NVQFAVGTAIALFMWITGILK--RPKISGAQLLAILPLAIVHTMGNLFTNMSLGKVAVSF
THTIKAMEPFFSVLLSAIFLGEFPTPWVVLSLVPIVGGVALASLTEASFNWAGFWSAMAS
NVTFQSRNVLSKKLMVKKE--E-----SLDNINLFSIITVMSFFLLAPVTLLTEG--VKV
SP----AVLQSA---GLN---LKQIYTRSLIAAFCFHAYQQVSYMILARVSPVTHSVGNC
VKRVVVIVTSVLFFRTPVSPVNSLGTGVALAGVFLYSQLKRL-----KPKA--KAA----
>Zmay_GRMZM2G174107_T01_395
--MQSAAAFRPCPTRLL-------VSSPCRPLLSARPL----------------RASAAG
AVATRSSA------------VGPRGLGLGLLPASPDRDGKCRQRQVSCSAAGDAVAAPKA
EEGGG------------LMKTLWL--GSLFGLWYLFNIYFNIYNKQVLKV--FPYPINIT
EVQFAVGTVAALFMWITGIIK--RPKISGAQLVAILPLAIVHTMGNLFTNMSLGKVAVSF
THTIKAMEPFFSVILSAIFLGELPTIWVVSSLLPIVGGVALASLTEASFNWAGFWSAMAS
NVTFQSRNVLSKKLMVKKE--E-----SLDNLNLFSIITVMSFFLLAPVTFFTEG--VKI
TP----TFLQSA---GLN---VNQVLTRCLFAGLCFHAYQQVSYMILAMVSPVTHSVGNC
VKRVVVIVTSVLFFRTPVSPINSLGTAIALAGVFLYSQLKRL-----KPKP--KTA----
>Zmay_GRMZM2G047404_T01_390
--MQSAAAIGLLRPCAA--------RPLAAYTSPRR------------------GAGACS
GGTQPLITPR----------GIRLSARPGLVPASPLEEKENRRCRASMHAAASAGEEAGG
G----------------LAKTLQL--GALFGLWYLFNIYFNIYNKQVLKV--LPYPINIT
TVQFAVGSAIALFMWITGIHK--RPKISGAQLFAILPLAIVHTMGNLFTNMSLGKVAVSF
THTIKAMEPFFSVLLSAIFLGELPTPWVVLSLLPIVGGVALASLTEASFNWAGFWSAMAS
NVTFQSRNVLSKKLMVKKE--E-----SLDNINLFSIITVMSFFLLAPVTLLTEG--VKV
SP----AVLQSA---GLN---LKQVYTRSLIAAFCFHAYQQVSYMILARVSPVTHSVGNC
VKRVVVIVTSVLFFRTPVSPINSLGTGIALAGVFLYSQLKRL-----KPKP--KTA----
>Zmay_GRMZM2G103047_T01_397
--MQSTAAIGLVRPCAA--------RPLPTYPSPRR------------------GAGACS
GAAQPILAPR----------GLCLSARASLLPDSPLEEEYRRCRAGRHVAAAGKVAAADG
AVEEAGGG---------LVKTLQL--GSLFGLWYLFNIYFNIYNKQVLKV--LPYPINIT
TVQFAVGSAIALFMWITGILK--RPKISGAQLFAILPLAIVHTMGNLFTNMSLGKVAVSF
THTIKAMEPFFSVLLSAIFLGELPTPWVVLSLLPIVGGVALASLTEASFNWAGFWSAMAS
NVTFQSRNVLSKKLMVKKE--E-----SLDNINLFSIITVMSFFLLAPVTLLTEG--VKV
SP----AVLQSA---GLN---LKQIYTRSLIAACCFHAYQQVSYMILARVSPVTHSVGNC
VKRVVVIVTSVLFFRTPVSPINSLGTGIALAGVFLYSQLKRL-----KPKP--KAA----
>Zmay_GRMZM2G066413_T01_415
--MMQGAAAGGTSVSGA---------SWARATRGRAAVLASRHVGV--------GASSSD
YYYNHFGPRGATAAPLLRARGGGRLRPLPLLSGSGKNGEVAKAAAAAASVPADDASAAAV
TTDGGG-----------IAATAQL--GAMIVAWYLLNIYFNIYNKQVLGALPLPLPYTIT
AFQLAFGSLLIFLMWATRLHP--APRLSAAQLGKIAPLAVGHMLGTVFTNMSLGKVAVSF
THTIKASEPFFTVVLSALFLGEVPSLPVLGSLVPIVGGVALASFTEVSFNWTGFWSAMAS
NLTNQSRNVLSKKLLAGDK--D-----VMDDINLFSVITVLSFLLSCPLMIFAEG--IKF
TP----GYLQST---GLN---LQELCVRAALAGLCFHGYQKLSYLILSRVSPVTHSVANC
VKRVVVIVSSVLFFSTPISPVNALGTGAALAGVFLYSRLTRT------KKP--KDA----
>Sbic_Sobic.004G353100.1_397
--MQSAAAFRPCPAGAG---------AGAGQLVSRNPS----------------RGPLLP
VPARPLRVVVSA--------ATTRALGLGRLRLSASPDDRSGQRQVSCGAAGDAVAAPSA
EEGGG------------FMKTLWL--GSLFGLWYLFNIYFNIYNKQVLKV--FPYPINIT
EAQFAVGSVVSLFFWTTGIIK--RPKISGAQLAAILPLAIVHTMGNLFTNMSLGKVAVSF
THTIKAMEPFFSVLLSAIFLGEFPTVWVVASLLPIVGGVALASLTEASFNWIGFWSAMAS
NVTFQSRNVLSKKLMVKKE--E-----SLDNLNLFSIITVMSFFVLAPVTFFTEG--VKI
TP----TFLQSA---GLN---VNQVLTRSLLAGLCFHAYQQVSYMILAMVSPVTHSVGNC
VKRVVVIVTSVLFFRTPVSPINSLGTAIALAGVFLYSQLKRL-----KPKP--KTP----
>Sbic_Sobic.002G159900.1_393
--MQSAAAVGLVRPCAA-------------RPLPTYPS----------------PRRGAG
ACSPPLLTTPR---------GLRLSARAGLVPASPLEEENRRCRHVAAAAGKVAAADTAG
EEAGGG-----------LAKTLQL--GALFGLWYLFNIYFNIYNKQVLKV--LPYPINIT
TVQFAVGSAIALFMWITGILK--RPKISGAQLFAILPLAIVHTMGNLFTNMSLGKVAVSF
THTIKAMEPFFSVLLSAIFLGELPTPWVVLSLLPIVGGVALASLTEASFNWAGFWSAMAS
NVTFQSRNVLSKKLMVKKE--E-----SLDNINLFSIITVMSFFLLAPVTLLTEG--VKV
SP----AVLQSA---GLN---LKQVYTRSLIAAFCFHAYQQVSYMILARVSPVTHSVGNC
```

VKRVVVIVTSVLFFRTPVSPINSLGTGIALAGVFLYSQLKRL-----KPKP--KAA----
>Sbic_Sobic.003G050800.1_418
--MQRGAAAAGTTSVSG-------ASSWTRAATRGLASRHVGVA----------SSSSSF
FGPRGATAAAQRLPLLRVRGGDGRLRPLSLLSDSGGKNGEVAKAVAAAAAASVPADDASA
AAVTGDRGG--------IAATAQL--GAMIVAWYLLNIYFNIYNKQVLGALPLPLPYTIT
AFQLAFGSLLIFLMWATRLHP--VPRLSAAQLGKIAPLAVGHMLGTVFTNMSLGKVAVSF
THTIKASEPFFTVVLSALFLGEVPSLPVLGSLVPIVGGVALASFTEVSFNWTGFWSAMAS
NLTNQSRNVLSKKLLAGDK--D-----VMDDINLFSVITVLSFLLSCPLMFFAEG--IKF
TP----GYLQST---GLN---LQELCVRAALAGLCFHGYQKLSYLILSRVSPVTHSVANC
VKRVVVIVSSVLFFSTPISPVNALGTGAALAGVFLYSRLTRT------KKP--KDA----
>Sbic_Sobic.009G062500.1_401
--MQSMAASCSSSSSSS--------SSRAWAAARRSPSPP--------------SLAFSS
SSSFPSSSHGCRWPV-----APRGIRGVLRPLPSPLLAPCVARAGAAAAASPPAAGKPEG
AAG--------------ISRTLQL--GAMILVWYLLNIYFNIYNKLVLKA--IPFPYTIT
TFHFASGSFFITLMWLLNLHP--KPRLSLKQYAKLLPLALIHMLGNVFTNMSLGKVAVSF
THTVKAMEPFFSVLLSVLFLGQTPSLLVLGSLVPVVGGVVLASMTEVSFNWIGFWSAMAS
NLTNQSRNVYSKKILADKE--D-----SLDDINLFSIITIMAFLLSAPLMLSVEG--IKF
SP----SYLQSA---GVS---VKELCVRAALAGTCFYFYQQVSYSLLARVSPVTHSVANS
LKRVVVIVSSVLFFRTPISPINALGTGVALAGVFLYSQFKKL-----KPKT--KAA----
>Bdis_Bradi4g27550.1_405
--MQSAAAIGLLRPCAA--------RPFLKNPSPGGA-----------------RLPASR
SALRLSAVAPRAGIA-----AAAGLGRIRLVPLSPEQEERSGRCSRDVAAAAASGKAAAG
EESGEEGGAA-------LAKTLQL--GVFFGLWYLFNIYFNIYNKQVLKV--FPYPINIT
TVQFAVGTVISLFLWITGILK--RPKISGAQLVAILPLAIVHTMGNLFTNMSLGKVAVSF
THTIKAMEPFFSVLLSAMFLGELPTPWVVLSLLPIVGGVALASISEASFNWAGFLSAMAS
NVTFQSRNVLSKKLMLKKE--A-----SLDNINLFSIITVMSFFLLAPVTLLTEG--VKV
TP----TFLQSA---GLN---LQQVYTRSLIAAFCFHAYQQVSYMILARVSPVTHSVGNC
VKRVVVIVTSVLFFKTPVSPINSIGTAIALAGVFLYSQLKRL-----QPKP--KAA----
>Bdis_Bradi2g04447.3_397
--MQRAAASLSSSRAAA----------WAAAASSRHAAG---------------ASCSAA
AGRRENTMAPP---------PLRILRGQQLLPLLPLLSGNRARRAVTASAAAAAELPAGD
DAAAGG-----------IAGAVEL--GAMIVAWYLLNIYFNIYNKLVLQA--LPFPYTMT
AFQLGFGSLVIFFMWAARLHP--APKLSAAQLARIAPLAAGHMLGTVFTNMSLGKVAVSF
THTVKASEPFFTVLLSAFFLGETPSLLVLGSLVPIVGGVALASLTEVSFNWVGFWSAMAS
NLLNQTRNVLSKRLLGGQQEEE-----SMDDINLFSVITVLSFLMSCPLMLLAEG--VKF
SP----AYLQST---GLN---LPELCVRAALAGLCFHGYQKISYMILARVSPVTHSVANC
VKRVVVIVSSVLFFRTPISAVNALGTGAALGGVYLYSRLKKS-----KP----KSI----
>Bdis_Bradi2g34300.1_418
--MQSMA-ATAAPAASSSYSASKGWAALRRCPSPSLAA--RHVAFSSSSSSARCTVAAAG
APVLPLGIRG----------SCLLLQSPLLTDGGKNGAATRRTVAAEAAAVASPSAEGDG
EPEAAG-----------IPRTVKL--GAMILVWYLLNIYFNIYNKLVLKA--VPFPYTIT
TFQFASGSFFITLMWLLNLHP--KPRLSLQQYAKILILALIHMMGNVFTNMSLGKVAVSF
THTIKAMEPFFSVLLSVLFLGETPPLPVLGSLVPVVGGVVLASMTEVSFNWIGFWSAMAS
NVTNQSRNVFSKKLLADKE--E-----TLDDINLFSIMTVMSFLLSIPLMLYVDG--IKF
SP----AYLQST---GIN---LQDLCLKAAIAGTCFHFYQQVSYSLLARISPVTHSVANS
VKRVVVIVSSVLFFRTPISPINAFGTGLALLGVFLYSRFKKA-----KPKV--KAA----
>Bsta_Brast05G093500.1_405
--MQSTAAIGLLRPCAA--------RPLLKNPSPGGA-----------------RLPAAR
GALRLSAVAPRAGIS-----AAAGLGRIGLLPLSPEQEERSGRCSRDVAAAAASGKAAAG
EGSGEEGGAA-------LAKTLQL--GVFFGLWYLFNIYFNIYNKQVLKV--FPYPINIT
TVQFAVGTVISLFLWITGILK--RPKVSGAQLVAILPLAIVHTMGNLFTNMSLGKVAVSF
THTIKAMEPFFSVLLSAMFLGELPTPWVVLSLLPIVGGVALASISEASFNWAGFLSAMAS
NVTFQSRNVLSKKLMLKKE--A-----SLDNINLFSIITVMSFFLLAPVTLLTEG--VKV
TP----TVLQSA---GLN---LQQVYTRSLIAAFCFHAYQQVSYMILARVSPVTHSVGNC
VKRVVVIVTSVLFFKTPVTPINSIGTAIALAGVFLYSQLKRL-----QPKP--KAA----
>Bsta_Brast01G368500.1_401
--MQRAAASLSSSRAAA--------WAAAAASSRHAPG----------------ASSCSA
ADRRDAAIAPP---------PLRVLRGQQLLPLRPLSGNRARRAVTASAAAAAAELPAGD
EAAVDTDGG--------IAGAVEL--GAMIVAWYLLNIYFNIYNKLVLQA--LPFPYTMT
AFQLAFGSLVIFFMWAARLHP--APKLSAAQLARIAPLAAGHMLGTVFTNMSLGKVAVSF
THTVKASEPFFTVLLSAFFLGETPSLPVLGSLVPIVGGVALASLTEVSFNWVGFWSAMAS
NLLNQTRNVLSKRLLGGQQEEE-----SMDDINLFSVITVLSFLMSCPLMLLAEG--VKF
SP----AYLQST---GLN---LPELCVRAVLAGLCFHGYQKISYMILARVSPVTHSVANC
VKRVVVIVSSVLFFRTPISPVNALGTGAALGGVYLYSRLKKS-----KP----KSI----
>Bsta_Brast08G188700.1_413
--MPSMAATAASSSYAA---------SKGWAVLRRCPSPSLAARHVAFSSSSSSSRCPVA
GAVAPVLSLGIR--------GGRLLLQSPLLPDGGKNGAATGRTVAAAAVASPSAEGGGE
PKAAG------------IPRTVQL--GAMILVWYLLNIYFNIYNKLVLKA--VPFPYTIT
TFQFASGSFFITLMWLLNLHP--KPRLSLQQYAKILILALIHMMGNVFTNMSLGKVAVSF
THTIKAMEPFFSVLLSVLFLGETPPLLVLGSLVPVVGGVVLASMTEVSFNWIGFWSAMAS
NVTNQSRNVFSKKLLADKE--E-----TLDDINLFSIMTVMSFLLSIPLMLYVEG--IKF
SP----SYLQST---GIN---LQDLCLKAAIAGTCFHFYQQVSYSLLARISPVTHSVANS
VKRVVVIVSSVLFFRTPISPINAFGTGLALLGVFLYSRFKKA-----KPKV--KAA----
>Osat_LOC_Os08g25624.1_407

```
-MQSAAAAFGLVRPCPA------------RPPLQLGPGSSSCRP----------ILLHAR
PLAAGIASSSRGPAAVAARSLGRLLLLPPPPPISPDRAGRGRARHVACGAAAGDAKAEEE
ESG--------------LAKTLQL--GALFGLWYLFNIYFNIYNKQVLKV--FPYPINIT
TVQFAVGTVVALFMWITGILR--RPKISGAQLFAILPLAVVHTMGNLFTNMSLGKVAVSF
THTIKAMEPFFSVLLSAIFLGELPTVWVILSLLPIVGGVALASLTEASFNWAGFWSAMAS
NVTFQSRNVLSKKLMVKKE--E-----SLDNINLFSIITVMSFFLLAPVAFLTEG--IKI
TP----TVLQSA---GLN---VKQVLTRSLLAALCFHAYQQVSYMILARVSPVTHSVGNC
VKRVVVIVTSVLFFRTPVSPINSLGTAIALAGVFLYSQLKRL-----KPKP--KAA----
>Osat_LOC_Os08g25624.2_401
-MQSAAAAFGLVRPCPA------------RPPLQLGPGSSSCRP----------ILLHAR
PLAAGIASSSRGPAAVAARSLGRLLLLPPPPPISPDRAGRGRARHVACGAAAGDAKAEEE
ESG--------------LAKTLQL--GALFGLWYLFNIYFNIYNKQVLKV--FPYPINIT
TVQFAVGTVVALFMWITGILR--RPKISGAQLFAILPLAVVHTMGNLFTNMSLGKVAVSF
THTIKAMEPFFSVLLSAIFLGELPTVWVILSLLPIVGGVALASLTEASFNWAGFWSAMAS
NVTFQSRNVLSKKLMVKKE--E-----SLDNINLFSIITVMSFFLLAPVAFLTEG--IKI
TP----TVLQSA------------VLTRSLLAALCFHAYQQVSYMILARVSPVTHSVGNC
VKRVVVIVTSVLFFRTPVSPINSLGTAIALAGVFLYSQLKRL-----KPKP--KAA----
>Osat_LOC_Os09g12600.1_408
--MQSAAAVGLLRPCGA--------TTAAAPLQLRNPS----------------PRGFGV
GVGQPLLPPRGLRLSAVAPRAGISARRIGLVPASPEQEDERRRGARDVAVAATAAAAGEA
GAEEGGG----------LAKTLQL--GALFGLWYLFNIYFNIYNKQVLKV--FPYPINIT
NVQFAVGTVIALFMWITGILK--RPKISGAQLAAILPLAMVHTMGNLFTNMSLGKVAVSF
THTIKAMEPFFSVLLSALFLGEMPTPFVVLSLVPIVGGVALASLTEASFNWAGFWSAMAS
NVTFQSRNVLSKKLMVKKE--E-----SLDNITLFSIITVMSFFLLAPVTLLTEG--VKV
TP----TVLQSA---GLN---LKQIYTRSLIAAFCFHAYQQVSYMILARVSPVTHSVGNC
VKRVVVIVTSVLFFRTPVSPINSLGTGVALAGVFLYSQLKRL-----KPKP--KTA----
>Osat_LOC_Os05g07870.1_414
--MQAVAAATSRAWAAS-------PRRRRHVASCSSPPPPTTTATTTTSSLNRCPVAGAG
APVLPL--------------GIRGGRMLLAPPLLWNSGAAARKAAVATAAAASPPAEGGG
KANGGAVAGG-------ISRTVQL--GAMILVWYLLNIYFNIFNKLVLKS--VPFPYTIT
TFQFASGSFFITLMWLLNLHP--KPRLSLGQYAKILPLALVHTMGNVFTNMSLGKVAVSF
THTIKAMEPFFSVLLSVLFLGETPSFLVLGSLVPIVGGVVLASMTEVSFNWIGFWSAMAS
NLTNQSRNVFSKKLLADKE--E-----TLDDINLFSIMTVMSFLLSAPLMLSVEG--IKF
SP----SYLQSN---GVN---LQELCMKAALAGTCFHFYQQVSYSLLARVSPVTHSVANC
VKRVVVIVSSVLFFRTPISPINALGTGVALAGVFLYSRFKKA-----KPKA--KTA----
>Osat_LOC_Os01g07730.1_393
--MQRAAAASRATA-----------WSTARHGAARVTA----------------SASFSG
GGGIVAGAA-----------LPLRVRGGQLMSLPLLSGGRAVTARVAAAEAPLPADDADA
AAGRERGA---------LAETAQL--GAMIVAWYLLNIYFNIYNKQVLQP--LPFPYTIT
AFQLAFGSFVIFLMWALKLHP--APRISISQLAKIAPLAAGHMLGTVFTNMSLSKVAVSF
THTIKASEPFFTVLLSAFFLGETPSLLVLGSLVPIVGGVALASLTELSFNWIGFWSAMAS
NLLYQSRNVLSKKLLGGEE--E-----ALDDINLFSILTILSFLLSLPLMLFSEG--VKF
SP----GYLRST---GLN---LQELCVRAALAGFCFHGYQKLSYLILARVSPVTHSVANC
VKRVVVIVASVLFFRTPISPVNALGTGVALGGVFLYSRLKRT-----KP----KNA----
>Macu_GSMUA_Achr2T14180_001_420
MHLRSAAFSAARPPLPM---------LRAGSNLPDGSHCSVPTVSSSSSSSFSTDLPSFL
PRIRPLSCRPTRFFL-----TPRLANQKSSLGLTTVVKAAEDVAAAAAGGTENHGEAAVS
SYGSSSTSG--------MVKKLQL--GFLFGLWYLFNIYFNIYDKQVLKV--YPFPITIT
TIHFAIGTVLIWLMWVTNLYK--RPKISSKQLAAIVPLAIVHTLANLFTNMSLGKVAVSF
THTIKALEPFFTVVLSAMFLGELPTLWVLISLVPIVGGVAMASLTEASFNWAGFWSAMAS
NLTNQSRNVLSKKIMDKDE--E-----TMDHITLFSIITVMSFFLLAPVSLLVEG--IKF
TP----SYMRFA---GLN---LKEVYVRSFLAGVCFHAYQQASYMILAEVSPITHSVGNC
VKRVVVIISSVLFFRTPVSPLNSIGTGVALIGVFLYSRVKRT-----KS----KSA----
>Macu_GSMUA_Achr3T28500_001_368
--MGAAVILSTVTFASC-------------------------------------AVAAAS
--------------------LTAATVRAGTVVAPDLAASDVQSWGTVTNSQAAAASLRER
RD---------------LLQTLLL--GSLFGLWYLFNIYFNIYNKQVLKV--FQFPLTIT
LLQFSIGTFLVLFMWTTNLYK--RPKISPMQLAAILPLALVHTMGNLFTNMSLGRVSVSF
THTIKAMEPFFSVLLSALFLGEVPTIWVILSLMPIVGGVALASLTEASFNWAGFWSAMAS
NLTFQSRNVLSKKVMVKKE--E-----SLDNINLFSIITIMSFFLLAPVTLFVEG--IKI
TP----TYLESA---GLN---LKQIYLRSLIAGLCFHAYQQVSYMILSRVSPVTHSVGNC
VKRVVVIVTSVLFFRTPVSPINALGTGVALGGVFLYSRVKRI-----KP----KST----
>Macu_GSMUA_Achr1T25520_001_398
--MQSAAISPSAAPPLL--------RSSTKPPAPRWSPLLL-------------PLHRGG
PRLRPLAAVPSFSGCCRRCSSGRCLVSGTSSTSTSTSTISRHVSPRRLDAFSCKPGTGYS
NSSWPD-----------TVTLLKAEPGRIFA------------NTRVLKG--FTFPLTIT
TVQFAVGTFLVLFMWTTNLYK--RPKISASQLAVILPLAMVHTMGNLFTNMSLGKVAVSF
THTIKAMEPFFSVLLSALFLGEMPTIWVLLSLVPIVGGVGLASLTEASFNWAGFWSAMAS
NLTFQSRNVLSKKAMVKKE--E-----SLDNINLFSIITIMSFCLLAPATFFVEG--IKV
TP----SYLQSA---GLN---FKEIYLRSLLAALCFHAYQQVSYMILARVSPVTHSVGNC
VKRVVVIVTSVLFFRTPVSPINSLGTGVALAGVFLYSRVKKI-----KP----KSA----
>Atri_v1.0_scaffold00096.30_407
--MQSSA-ISLSSCNPF---------LKSKSPCKPTSG----------------FPPRFS
RGLDPLPPKGLHLRASIGRSDLRQRISFGNGVSPSMQKPLFEGPDFRVGASASVPESADS
```

```
KSSSSS-----------LIQTLQL--GALFGLWYLFNIYFNIYNKQVLKV--FPFPITIT
TLQFAVGTVLVLIMWSTGLYK--KPKINSSQLIAILPLAAVHTLGNLFTNMSLGKVAVSF
THTIKAMEPFFSVVLSALFMGELPTLWVVSSLVPIVGGVALASLTEASFNWAGFWSAMAS
NLTNQSRNVLSKKFMLKKE--E-----SLDNITLFSIITIMSLIMLAPVTVFTEG--IKF
TP----SYMELA---GLN---VEQVIQRSLLAGLCFHAYQQVSYMILARVSPVTHSVGNC
VKRVVVIVSSVLFFKTPVSPINSLGTGIALAGVFLYSRVKKI-----KPKPEAKSA----
>Smoe_172535|172535_307
------------------------------------------------------------
------------------------------------------------------------
-----------------MMRTLQL--GSLFGLWYLFNIYFNIYNKQVLKV--FPFPITIT
EIQFAIGSAAVLFMWTTGLYK--RPSLTTAQVVAILPLALVHTMGNLFTNMSLGKVAVSF
THTIKAMEPFFSVLLSAMFLGEAPSAWIIASLLPIVGGVALASLTEASFNWAGFLSAMAS
NVTFQSRNVLSKKLMVKKE--G-----SLDNINLFSVITILSFFLLAPVTLFFEG--VKF
TP----EYLTSM---GLD---VKVVMLRALVAGLCFHSYQQVSYMILQRVSPVTHSVGNC
VKRVIVIVTSVIFFRTPVSTINALGTALALAGVFAYSRAKRI-----KPAK--KSA----
>Mpus_3748_320
------------------------------------------------------------
------------------------------------------------------------
-------------------KTLIL--GVLFAGWYACNIVFNICNKQVLGA--YPFPLTST
LWQFAAGVAFTALLQMTGIHRINKDALTMESLRAIAPLAIVHTLGNVLTNVSLGKVAVSF
THTIKAMEPFFSVLLSSLFLGDVPSAAVIATLVPIVGGVAAASVTEASFNWPGFLAAMGS
NVTFQSRNVLSKKLIGGDGCSQACPAIPMDNIDLFSIITIMSLALTLPAAVVLEG--VRF
TPGAIAAYAASAGA-AFS---PAVIFQKAMIAGACFHMYQQISYMILARVSPVTHSVGNC
VKRVVVISFSVLFFKNAVSPVNAVGTAAALGGVYAYTRVKRA-----ERDA--AAA----
>Crei__Cre06.g263850.t1.1_401
--MASLLGRKACIPATC----------VQKSLTPPAPL----------------RLGLAC
SALQRHQVQ-----------TAIASRVARQQEENGRAAAIVPRQRTVCQAAAVPADGESD
KGKD-------------MSGMMVL--GLMFVAWYGTNIFFNIYNKQLFKV--FPFPLTTT
NIQFFIGSCLSMVFWVTGIVK--LPKIDMALVKSIYPLAIINVLGNVLTNVSLGHVAVSF
THTVKAMEPFFSVIFSAIFLGDVPPVPVLLTLVPIVGGVVIASLTEATFNWTGFLSAIFS
NMTFQSRNVLSKKLMIKKG--------AVDNMNLFQIITIMSFLMLLPVSTMVEGGAALL
TP----ESLANL---GLNEAAREQMFMRLLSAGICFHSYQQLSYMILSRVAPVTHSIGNC
VKRVVVIVASLIAFQNPISMQNAIGTGIALFGVFLYSQAKRKYKGKGDVKP--EAA----
>Crei_Cre08.g379350.t1.1_399
--MSSLLKTRVVPSLAA-------------------------------------RDVAFC
HPLVFTTSSQR---------VARASGQSSAFPLRSAVSGVSSRRPFTCLAVAASAGDVSD
GSSHTE-----------MMQTLVL--GSMFAGWYAANIAFNIYNKQLLKA--FAFPLTIT
EAQFLVGSCVTLVAWGSGLQR--APKITWSTIKNVLPLAVVHTLGNLLTNMSLGAVAVSF
THTIKAMEPIFSVALSALFLGDQPSPLVLATLLPIIGGVAMASMTEATFNWFGFLSAMGS
NLTFQSRNVLSKKLMLKKKDKDGNAEAPLDNMALFSVITLLSAALLLPATLLFEG--WKL
SP----VGLAEM---GVR--SPNGVLAHAAMAGLCFHLYQQVSYMILSRVSPVTHSIGNC
VKRVVVIAASVLFFRNPVSLQNALGTALALAGVFLYGTVKRQ-----QAIAAGKKIAASE
>Mpol_Mapoly0083s0074.1_440
--MVSQALAMLQPQACCVSSSRC--AGLSGAAFSSASGVKSIVSSYAVSRRDGRALSSKA
AASKFGSSFLSGPAV-----SRSGLFPLNASLNSRLEERRPRKGSLVVRASAVESGADEG
AAPDGSTSPVVPAQPSELAKTLQL--GSLFGLWYLFNIYFNIYNKQVLKV--YTFPVTMT
NMQFAVGGVIVMIMWLTGLHK--RPKITTSQLLAILPLAIVHTMGNLFTNMSLGKVAVSF
THTIKAMEPFFSVLLSALFLGEVPNPWVVASLLPIVGGVALASLTEVSFNWAGFLAAMAS
NVTFQSRNVLSKKLMVKKE--G-----SLDNINLFSVITVMSFFLLAPVTYLMEG--VKF
TP----AVIQAA---GLD---VKVIATRALLAGLCFHSYQQVSYMILQRVNPVTHSVGNC
VKRVVVIVTSVLFFKTPVSPINALGTGIALSGVFAYSRVKALGKGSKKEEK--KEA----
```
