# Supplementary material for: What Matters for C4 Transporters: Evolutionary Changes of Phosphoenolpyruvate Transporter for C4 Photosynthesis
Source: Front Plant Sci. 2020 Jun 30;11:935. doi: 10.3389/fpls.2020.00935 (PMC7338763; doi:10.3389/fpls.2020.00935)
Supplement: Supplementary file 3 [file DataSheet_2.pdf]

```
CLUSTAL multiple sequence alignment by MUSCLE (3.8)


####MEM1 B submodule present in PEPC, PPT1 and PPDK of Ftri (C4)

Ftri_PEPC       GAGCTGTACTTACTCACTAA----------------------------------------
Ftri_PPDK       -TTTTCGTCCTTGGCACTAACTTTGTTAGTTTTTACAGTTAATAAGAGTATTCAGGTCAT
Ftri_PPT1       -ATTACCATCTACCCACTAA----------------------------------------
                          *   ******

Ftri_PEPC       -------------AACAAACAAAAAA--------------------------------CT
Ftri_PPDK       TTTAAATTTAGGGACTAGTTATGTAAATTATATTAGATTAAGATAATTGTTGTAAATTTT
Ftri_PPT1       -------------------TATATAA--------------------------------TT
                                    *   **                                 *

Ftri_PEPC       AAAACAAACAAAAAAC -1498
Ftri_PPDK       TAAAACAACAAAAAAA -1500
Ftri_PPT1       AAAGAAAACAAAAAAC -2740
                 **   *********


####### PPT1 from Fram (C3-C4) and Ftri (C4)

Ftri_PPT1A_3k      TTCATCTTATAACTTTATTTATTTTTTATAATTAATTGTTTATTTAATTTATAACGCTTT
Fram_PPT1A_4k      ---------------------TTTTCTGCAGGAAAACGTCTG--CAGTCTGCG-------
                                        **** *  *   **  ** *    * * *

Ftri_PPT1A_3k      AATTATATTAAAAAA-------ACCAACCGGTGTGGGCCTAAACCGGTTTTCAAACCGAC
Fram_PPT1A_4k      GACCACATCTGCAGACATCTGCAGCAGAAGAGGTGGACCAAA---TGTCTGTAGTCTG--
                    *  * **    * *       * **   *  **** ** **    ** *  *  * *

Ftri_PPT1A_3k      CCAAACCGATTTAAAACCCAAACTGGTTTTAAC--------CATGGTTGACCAAACCGGT
Fram_PPT1A_4k      -CAAGA------AAAAGACTGTTTGTTTTTAACATCTGCAGAATGGTAAA--AAATATTC
                    ***        ****  *    ** *******         *****  *  ***

Ftri_PPT1A_3k      TTTAACCATGGTTGACCAAACCGGTTTTAAAAAAACTCCCGATTTGTAC----ACCATTA
Fram_PPT1A_4k      TGTAATTA-----GATTAAACCTATTTTTTAACACTTGTTTTCTTGGACTTGTACCA---
                   * ***  *     **  *****  ****  ** *  *      *** **    ****

Ftri_PPT1A_3k      CCATCTACCCACTAATATATAATTAAAGAAAACAAAAAACA----TAAAAATGTTTTCAT
Fram_PPT1A_4k      -CACACACACATCCGTACGTACATACAACATATATACAACCGTATTGTATATACTTTTGT
                    **   ** **    **  **  ** *  * * * * ***     *  * **  ***  *

Ftri_PPT1A_3k      A----CACATGATAAACTCACGTGCA-------CTCAACCCAAGTAAATTGATGTTGCAC
Fram_PPT1A_4k      GTTACCATTTTATATACTTTTGTGTCTCTTTTTTTTGTCTCACACGTATTG----TATAT
                        **  * *** ***   ***          *   * **     ****    *  *

Ftri_PPT1A_3k      TT---CAACAAACAATTCAATTCATTCACCTAAGTGACCCGTGTATTTCCCATCTTATTT
Fram_PPT1A_4k      TTGTACACCACACTATGCAATACAATAACTTAAG--------GCATTCTATATTATATAA
                   **   ** ** ** ** **** ** * ** ****        * ***    **  ***

Ftri_PPT1A_3k      AGTTTTCTATGTATATGACTATTTAAT--GCATAAAAGTTGGAC--ACAAGAATTTTCCT
Fram_PPT1A_4k      GGTTTTTTAT-TAAATTATTATGGGATCGTCTTTAATAGTAAACCGACAAGAATTGTAGT
                    ***** *** ** ** * ***   **   * * **   *  **  ********* *  *

Ftri_PPT1A_3k      ATTTAATGTAGTG--------------AAGTCTAGACACATCCAA-----------ACAC
Fram_PPT1A_4k      A-GTACTGTAGTAGTATTTTTTTTATTAAATCGAATCGAATGGAATGGAATGGAGTGGAA
                   *  ** ******               ** ** *  *  **  **             *

Ftri_PPT1A_3k      TAGTTTATAAATCGGATGAATAGATACATAGGGATATCGTTATGC---------------
Fram_PPT1A_4k      TATATTAAGAATCGAATCGGTGGGGGTGGTGGGGCGGCGGTGGTCCGGCCGGTGGGGGTG
                   **  ***  ***** **   * *       ***    ** *   *

Ftri_PPT1A_3k      ------------------------------------------------------------
Fram_PPT1A_4k      GTGGGGCGGCGGTGGTCCGGCCGGTGGGGGTGGTGGGGCGGCGGTGGTCCGGCCGGTGGG


Ftri_PPT1A_3k      ------------------------------------ACATGATGTACTCGA---------
Fram_PPT1A_4k      GGGAGGTGGGGCGGCGGTGGTCCGGCCGGTGGGGGGAGGTGGGGCGGCCGATGGGGGGAG
                                                       *  **  *    ***

Ftri_PPT1A_3k      ----------TCAAGGCGATCCAAACATATTAGAAGTTTTTAAAACGATCAATGT---TA
Fram_PPT1A_4k      GTGGGGGAGGTGAGGGAGGTGAGAAGAGGTTAGAAGATCTCAGAA-GACATAGGTCCACA
                             * * ** * *   ** *  ******* * * * ** **   * **    *

Ftri_PPT1A_3k      TTTTTTAAGATCAATCATACGTTGCGTA-ATATTTTAAATTTATAGAAGGTTT----GAG
```

```
Fram_PPT1A_4k       TCTTCT----CCATGCAGACCTCCTGCAGACATTTT---TTAATGAAATGTCTTTCAGAA
                    * ** *     **  ** ** *   * * * *****   ** **  ** ** *    **

Ftri_PPT1A_3k       GACCGATAGTGTTGTACTAT--------------------------------ATTCATAG
Fram_PPT1A_4k       AACAAACAGT-TTGCGCAATCAAAACCTCTTTCCGCGTCTGCGTGCGCGCAGATGAATAG
                     **  * *** ***  * **                                **  ****

Ftri_PPT1A_3k       GTG------------------------GCCTTTGT----------------CTATGTTTA
Fram_PPT1A_4k       GTGGGGAAATGAAGAGGTGGGGAAGATGTTTTGGTAAAAAACAAACAACACCTATGTTTG
                    ***                        *  ** **                ******** 

Ftri_PPT1A_3k       GTGTTCGTTTATAAGAGAGTTGATTATTTTATCCTTTTGTATTGCATTTATAATACATTT
Fram_PPT1A_4k       GTGTTCGTTTACAAAAGAGTTGATT-TTTTATCATTTTGTATTGCAGTTATAATACA-TT
                    *********** ** ********** ******* ************ ********** **

Ftri_PPT1A_3k       TTTTTCATATTATGTGAGTTTATTATAGTTTATTGACTACGGAAAACATCATAACCACTT
Fram_PPT1A_4k       TCCTTCATATTATGTGAGTCTATTATAGTTTACTGACTACTGAAAACATCATAACCACTT
                    *  **************** ************ ******* *******************

Ftri_PPT1A_3k       ACCACATGTCACGACTCATGGCCACCCACTGCATTTGCACTTTTGTCATGATGACAACTT
Fram_PPT1A_4k       ATCACATGTGGCGACTCATGGCCACTCATTGCATTTGCATTTTTGTCATGATGACAATTT
                    * *******  ************** ** ********** ***************** **

Ftri_PPT1A_3k       CATAGTACAAGAGTCCCGGCCATCGTATATTTTAACATTTTGCGTCGTATCAATAGAATT
Fram_PPT1A_4k       CACAGTACACGAGTCCCAACCATCGTATATTTTAATGTTTTGCGTCGTATCAATGGAGTT
                    ** ****** *******  ****************  ***************** ** **

Ftri_PPT1A_3k       TAACGTTGAGCGTTGTTAGTGTTCGCACCTTCTCGCCTTTAGATAAATTTATAACATCTT
Fram_PPT1A_4k       TAATGTTGAACGTTGTTAGCGTTCACACCTTCTCG-CTTTAGATAAACTGATAACATCTA
                    *** ***** ********* **** ********** *********** * ********* 

Ftri_PPT1A_3k       GAAGTTGTTTTCACCTTGTTAATTTAGTGATAGGCGTTAACAATACAATATGGCATTAGT
Fram_PPT1A_4k       GAAGTTGTTTTCACCTTGTTAATTTAGTGATATGCGTTAACAATATAATATGACGTCGGT
                    ******************************** ************ ****** * *  **

Ftri_PPT1A_3k       AATCAATTGTGCTTTTTTGAGAGACATTTTCCCTGATAGAGAGACATATTGGGGATGAAG
Fram_PPT1A_4k       AATCAACCATGTTTGTTTGAGAGACATTTT-CCTGATAGAAAGACGTGTTGGGGATGAAG
                    ******   ** ** *************** ********* **** * ************

Ftri_PPT1A_3k       TCTAAAATAATTACTTTGGTTGTAGTTTATGCATTGTTAAAACATGATGATCCAAATCAT
Fram_PPT1A_4k       TCCAAAATAATT-CTTTAGTAGTAGTATATGTATTGTTAAAACATGATGATCCAAAACAT
                    ** ********* **** ** ***** **** ************************ ***

Ftri_PPT1A_3k       CTAATAAAACACTTATTTTACGGGATGTCCTAGCACCATCCATTTTTTTGAGCAAAACAT
Fram_PPT1A_4k       CTAATAGAGCGATTCTTTTACGCGTTGTCCCACCACTACCCATTTATTTGAGCAAAACAT
                    ****** * *  ** ******* * ***** * *** * ****** **************

Ftri_PPT1A_3k       GGATTACATTCGAACCATGTCGAGCTATTTTTGGAGTATTATTTTGTTTAAATCGCTTGC
Fram_PPT1A_4k       GGATTACATTCGAACCATATCGTGTTAATTAAGGCGTATTATTTTGTTCAATTCATTTGC
                    ****************** *** * ** **  ** ************* ** **  ****

Ftri_PPT1A_3k       CAAATACGTAAAGTAAAAACAACTTTCAGACACCCTCAAATTAACATAAGACTAGAAAAA
Fram_PPT1A_4k       CA-------AAGGTAAAAGCAACTTTCAGATGCTCTCAAGTTAACATAAGACTAGAAATT
                    **       ** ****** ***********  * ***** ******************  

Ftri_PPT1A_3k       TTGTTGTAAAAAATATCATATGATTTTTT----------------TAACATTTTTAATTA
Fram_PPT1A_4k       TTATTGGAACATGTACAAGATAAATTCTTATTAATGCTTGCTTAGAAACATATTTAATCC
                    ** *** ** *  **  * ** * ** **                 ***** ******  

Ftri_PPT1A_3k       -ATTATTACAAGTAACTAGATGTTGATGTATCTCACAATAT-----ACATATTATTGAGA
Fram_PPT1A_4k       TGATATTATAAAT-CCTAGA----AATCTAATTTACGATAGAAATAACATGTTAATGTTA
                       ***** ** *  *****     ** **  * ** ***      **** *** **  *

Ftri_PPT1A_3k       -ATAAAATTTGA----TACACAAAATCATTATCTTGAAATATAACACAACCAAACTGC--
Fram_PPT1A_4k       TATAACTCTTAAGTAGTTCCCATAAAGACACTCCATATCTACAACAAAGACACTTTGTTC
                     ****   ** *    * * ** **  *   **   *  ** **** *  **   **   

Ftri_PPT1A_3k       -----CGTTATTTTTTGTGATTGCT-----------ATGTAGT-TATTTTTTGTG---AT
Fram_PPT1A_4k       ATTTACTTTAGCTTTCATAATTGTTTTATTGAATAAATATAATATATTTTAAGTGTTTAT
                         * ***  ***  * **** *           ** ** * ******  ***   **

Ftri_PPT1A_3k       TGTTATTT--------------TTACTATATA-ATGTGG-----AATATAATGTG-----
Fram_PPT1A_4k       CGTCATTCAAACACCAAAAACATTACAATATGCATGTGGTAATTAGTATACTGTGAACTA
                     ** ***               **** ****  ******     * **** ****     

Ftri_PPT1A_3k       GTCTCTACGAGTTGTG------------------------ATATCTAGTGATG---AACG
```

```
Fram_PPT1A_4k      GTTTTTAGAAACCGTGTGAATGAATATATTAAAGTAATATTTATCTATTAATATTTAAAA
                   ** * **  *   ***                         ****** * **     **

Ftri_PPT1A_3k      AGTTAAAAAT--ATTCTTGGTTGAGGGTTGGTGA----AAGTTTGGTAAGGGGGTTGGGG
Fram_PPT1A_4k      AATAAAAAATAAATTTTGAGATAAATACGATTGAAATTAAGTATAATTAGTTACTTTAGG
                   * * ******  *** *  * * *       ***    **** *  * **    **  **

Ftri_PPT1A_3k      ---------------AAGGTCTTTTTA----------------------TAGCGTATGTT
Fram_PPT1A_4k      TAATTAAACAGTAACAATATTTTTTTAAACTTTCGTCATTAAAAATTTTTTGCTTAAAAA
                                  **  * ******                      * ** **

Ftri_PPT1A_3k      TTTGTGGTTTTA------------TTTAATACATTAAT----------------------
Fram_PPT1A_4k      TTTGCTTTTTTAAATACTCCAAGTTTTAAAAAAATAATATTTTAAAAAAATGTTTTTTAA
                   ****   *****            ***** * * ****

Ftri_PPT1A_3k      --TTCTTTTTGTAAAA---------------------------TTTTTAAAAGAGGAAAA
Fram_PPT1A_4k      AGTTCTTTGTATAAAAATACTTCATATTCTATAAAACTTTATTTTTTTAAAAAATTACAA
                     ****** * *****                           ********* *  * **

Ftri_PPT1A_3k      TATATTAGTATATACGTGACATACATCTCTCTACTTTAATAAAATGATAAATGTGAGTGA
Fram_PPT1A_4k      -ATTTTAATAAAAA---GAAACATATCTATCTACTCTAATTAAAATGCATCTTGGGATGA
                    ** *** ** * *   ** * * **** ****** **** ***    *  *  *  ***

Ftri_PPT1A_3k      TACGTGTTAGCGTCACAATCATTCTCACCAAAAGTTTTTCGTCTCTCTTTCATATTAAAT
Fram_PPT1A_4k      CACATGACAACACCATAATCATTCTCATCAAAATTTTCCCGCCTCTCTTTCTTATTAAAT
                    ** **  * *  ** *********** ***** ***  ** ********* ********

Ftri_PPT1A_3k      AATTATCTTAATGACTAATTATATTGTTTTAATACATTTTAATATTTAATAAATTAAGAA
Fram_PPT1A_4k      AATTACCTTAATTGCTAATAATATTA-ATTAACACCTTTTTAGATTT-----ATTAAGAA
                   ***** ******  ***** *****   **** ** **** * ****     ********

Ftri_PPT1A_3k      AGAAATCTCTTTCTTAAGGTTATTTCTAATAAATTGA--TATTATTAAAAAGTCAATT--
Fram_PPT1A_4k      ATAAATCTATTTATTAATGTTATTTTAAATAAATAAAGTTTTTATAAAATAATAAATTTT
                   * ****** *** **** *******  *******  *  * **** *** * * ****

Ftri_PPT1A_3k      --TAAAAAAAAAAGT----------AATCAAAT---------TTGGGAGTTGTAATAAGA
Fram_PPT1A_4k      TCTAAAGATAAAACTTTTTTTTAAAAATGAAATATTTTAAGATTGGTATTTTTAAAAAGT
                     **** * **** *          *** ****         **** * ** *** ***

Ftri_PPT1A_3k      AACTTTTTAAAAATAACA-----------TT-----TTATACGGTTTGTTTTAAAACAAA
Fram_PPT1A_4k      CATTTTTTAAAAACAAAAAATAATCAATTTTGAGAGTTATACGGTTTGTTTTAAAACAAA
                    * ********** ** *           **     ************************

Ftri_PPT1A_3k      ATGTTTATTTTT------------------------------------------------
Fram_PPT1A_4k      ATGTTTATTTGTATTAGAATATCCTTTTACAATTAATAAATTTTTATATTAATATTTTAT
                   ********** *

Ftri_PPT1A_3k      -----------------------------------------------------AAAAAT-
Fram_PPT1A_4k      TTATTCAACCCGTGTAATACACGGTTTACTAAATCTAGTTTTTAATAAACCAAAAAAACT
                                                                        *****

Ftri_PPT1A_3k      -------------AAAAATAAAAATAAAAT--------TTTAA-----------------
Fram_PPT1A_4k      ATTTTATAATTTGAAAAATATAAATAATATGATTAGCATTTAATGTATCATTAGATGGTT
                                ******* ****** **        *****

Ftri_PPT1A_3k      ------------------------------------------------------------
Fram_PPT1A_4k      AACGCTAATGATCTCTTGCTTTAGTGGTTCAAGGGGAAGGTGGGAGGTTTTGCCTCTTTC


Ftri_PPT1A_3k      --------AAAGTTGA-----TATCACTTTTGTATTATTA--------------------
Fram_PPT1A_4k      GGATATCCAAAGTTCAAACCTTAGCATTGGTGAATTATTACTAACTAGGTGTTGCTAACT
                           ****** *     ** ** *  ** *******

Ftri_PPT1A_3k      ------------------------------------------------------------
Fram_PPT1A_4k      TCTAACAACTAATAAGCCGTTCGAAAAAAAGAGAAAAATATTTTTAATCAATATTAATGA


Ftri_PPT1A_3k      ----TAGTAATTTTTATACGTAATTTCAGT-------------CATATCA----------
Fram_PPT1A_4k      CACGTGTTAAATCCTAGGTGTAGCTTCGGTGTACAATGCAAAAAATATCATGTGACTTTT
                       *  *** *  **   ***  *** **              ******

Ftri_PPT1A_3k      ------------------------------------------------------------
Fram_PPT1A_4k      TAACATTTTTAATTAATTAGTATAAATAACTAGATGTAATTCATGTATCTCACAATATAT


Ftri_PPT1A_3k      ------------------------------------TATCTTAAAATATTA---------
```

```
Fram_PPT1A_4k      ATATTATTGAGAATAAAATTCGATTCACAAAATCATTATCTAGAAATATAACACAACCAA
                                                       *****  ****** *

Ftri_PPT1A_3k      ----------------------------------TTTTATAATTAATAAATTTATTATAT
Fram_PPT1A_4k      AATGTCGTTATTTTTGTGATTGCTATGTAGTTGTTTTTGTGATTTTTATTTTTATTATAT
                                                     **** * ***  **  **********

Ftri_PPT1A_3k      A-----------------------------------------------------------
Fram_PPT1A_4k      AATATGGAATAGGCCCGGTGGTCTCCACCGAGTTGTGATATCTAGTGATGAAAGAGTTAA
                   *

Ftri_PPT1A_3k      -AATATTTTA--------------------------------------------------
Fram_PPT1A_4k      GAATATTCTAGATTGAGGGTTGGTGAAAGTTTGGTGAGGGGGTTGGGGAAGGTCTTTTTA
                    ****** **

Ftri_PPT1A_3k      -----------TTTATTCAACCCATGTAATACACGGGTTT---------ACAAAGCTA--
Fram_PPT1A_4k      TAGCGTATGTTTTTGTTGGTTTTATTTAATACATTAATTTCTTTTTGTAAAAATGTTAAA
                              *** **      ** *******    ***         * ** * **

Ftri_PPT1A_3k      -------------------------------GTACATAAATAACACTTAAATTTTAAAGA
Fram_PPT1A_4k      AGAGGAAAATATATTAGTATACTTGATACACATACCTAAATACAACTTAAATTTTAAAGA
                                                   *** ******  ****************

Ftri_PPT1A_3k      TTTGATTGAATAAATTGAAATTTATTACAAGTAACTATAATTACCTAAAATAGCAATTCC
Fram_PPT1A_4k      TTTGATTGAATAAATTGAAATTTATTACAAGTAACTATAATTACCTAAAATAGCAATTCC
                   ************************************************************

Ftri_PPT1A_3k      ATTTCAAATTTGTTACATTTTTAGTAAAATATTTAAAGTTGCAGTTTGTATTCTGTAAAA
Fram_PPT1A_4k      ATTTCAAATTTGTTACATTTTTAGTAAAATATTAAAAGTTGTAGTCTGTACTCTGTAAAA
                   ********************************* ******* *** **** *********

Ftri_PPT1A_3k      AATTTCAATTACAAACCAATTAAACCAAAAATC-AACTAAATCAAAATTGTGTTTCACCC
Fram_PPT1A_4k      ATTTTCAATTACA--------AAACCGAAAATCGAATTAAGTCAAAATTGTGTTTCACCC
                   * ***********        ***** ****** ** *** *******************

Ftri_PPT1A_3k      ATTTCAAATTTGTTACTCAGTTAAGAACTTCAATTTGTTAAACTGAAAAAACCTTACATT
Fram_PPT1A_4k      GTCCCAAATTTGTTACTCAGTTAAAAACTTTAATTTGTTAAACTGAAAAAACCTTACATT
                    *  ******************** ***** *****************************

Ftri_PPT1A_3k      ACTCTAGTAAAACATTTAAAAAACTTGATTACAATAAAATGACCACTCTAAGAATATTTT
Fram_PPT1A_4k      ACTCTAGTAAAACATTTAAAGAACTTGATTACAATAAGATGAATAGGCTATGAATTTTTT
                   ******************** **************** ****  *  *** **** ****

Ftri_PPT1A_3k      TTATTTTTCTGTACACCTCGTTAAAAGTTTAGTCCTTTAACCGAGACCGAGTAAACCAAA
Fram_PPT1A_4k      TTATTTTTTTCTACAACCCGTTAAAAATTTAGTCCTTTAACAGAAATCGAGGAAACCAAG
                   ******** * **** * ******** ************** ** * **** *******

Ftri_PPT1A_3k      CTGATAAAAATCCAAAACCCCGAA----------------ACCCAAAACAATCTAGTTGT
Fram_PPT1A_4k      CTTAT-AAAATCCAAAACCCCGAAACCCAAACCAATAAACACCCAAAATAATCAAGTTGT
                   ** ** ******************                ******** **** ******

Ftri_PPT1A_3k      TTTTCAA--------TCAACACGCTCATCAAACCTACGATCATGTTTTACCTCACAGTCG
Fram_PPT1A_4k      TTTTCAATCAACAACTCAACACACTCATCAAACCTACGATCATACTTTACCTCACAGTCC
                   *******        ******* ********************  **************

Ftri_PPT1A_3k      ACAGTAAAACTCGAACCTACAACCCCGAAGAAACATATGCATATCATTTACAAGTTGAAA
Fram_PPT1A_4k      ACAGTAAAACTCGAACCACCAACCTCGAAGAAACATATACAT------------------
                   *****************  ***** ************* ***

Ftri_PPT1A_3k      ATAGCACAAGACTATTATAATTGCTAGGTTGGTGAGTCCAAATCTGATATCCCAAGAATA
Fram_PPT1A_4k      -----------------------------------------ATATGATATCCCAAGAATA
                                                            ** ****************

Ftri_PPT1A_3k      TCCCCAATATAAAAAACACCGAAAAAACGATAT-AAAACAAAGCAAAACCCCAACACAAA
Fram_PPT1A_4k      TCCCCAATATAAAAAACACCGAAAAAACGATATAAAACTAAACAAAACCCCAA-ACAAA
                   ********************************* ***** ** *********** *****

Ftri_PPT1A_3k      TTCAGTCTTCACTGCATAGGCGGAGCCTCCAAGACACCTTCTTCGTCTACGGTATCTACC
Fram_PPT1A_4k      TTCACTCTTCACTGCATAGGCGGAGCCTCCAAGACACCTTCTTCATCTACGGTATCTACC
                   **** *************************************** ***************

Ftri_PPT1A_3k      TACTCCGAGCATCAATCGGAGATCTCACAATA
Fram_PPT1A_4k      TACTCCGATCATCAATCGGAGATCTCACAATA
                   ******** ***********************
```

```
####### PPT1 four species

Frob_PPT1_3.5k      ---------------------CTTTTTAC-------------------------------
Fson_PPT1_3k        --------GGGGTTGTTGTTGCTTTGTGAGGTT---------------------------
Ftri_PPT1A_3k       TTCATCTTATAACTTTATTTATTTTTTATAATTAATTGTTTATTTAATTTATAACGCTTT
Fram_PPT1A_4k       TTTTCTGCAGGAAAACGTCTGCAGTCTGCGG-----------------------------
                                            * *

Frob_PPT1_3.5k      -CCCATATGAA------AAGTCAGTTTG--------------------------------
Fson_PPT1_3k        -ATTATATCA-------TAGCAAGTTTTGCCCACAAGTGTTGTTACAACGCTATCAAAG-
Ftri_PPT1A_3k       AATTATATTAAAAAAACCAACCGGTGTGGGCCTAAACCGGTTTTCAAACCGACCCAAACC
Fram_PPT1A_4k       -ACCACATCTG------CAGACATCTGCAGCAGAAGAGGTGGACCAAATGTCTGTAGTCT
                        * **          *

Frob_PPT1_3.5k      -----AAAAATCCTTTTG------AGTTTCCAA--------------------ACACTTT
Fson_PPT1_3k        -----AAGACTGCATCTACCGTGCAGCTATCATCAGC----AATTGTTGCACTAAA---T
Ftri_PPT1A_3k       GATTTAAAACCCAAACT-------GGTTTTAACCA---------TGGTTGACCAAACCGG
Fram_PPT1A_4k       GCAAGAAAAAGACTGTT-------TGTTTTTAACATCTGCAGAATGGTAAA--AAATATT
                         ** *       *        * *   *                     * *

Frob_PPT1_3.5k      TTTTGACTT-----------ATTAACTTTTGAAAAGTTAATAAGTCAAAAGTAACTTGAA
Fson_PPT1_3k        CTATAACTGCAGATGCAAAAATAAATGGATTAAAATTCTATTATTCAACATTTTTTTTGT
Ftri_PPT1A_3k       TTTTAACCATGGTTGACCAAACCGGTTTTAAAAAAACTCCCGA------------TTTGT
Fram_PPT1A_4k       CTGTAATTA-----GATTAAACCTATTTTTTAACACTTGTTTTCTTGGA------CTTGT
                     * * *              *          ** *                     *

Frob_PPT1_3.5k      AATAAGCAATCCCAAACACCCACTAAACGAATGTGTGCTTC-ACATTCAATCGGGGTTTC
Fson_PPT1_3k        ATTGTGCATTGTAAAATACCGGT---ACGAGTGTTCATCTTGAAATCCATGAGCAGGGCA
Ftri_PPT1A_3k       AC--ACCATTACCATCTACCC-----ACTAATATATAATTA-AAGAAAACAAAAAACATA
Fram_PPT1A_4k       ACCACACA----CACACATCCGT---ACGTACATACAACAT-ATATACAACCGTATTGTA
                    *     **     *   * *      **     *        *     *

Frob_PPT1_3.5k      GGTATGGTAGTGAGGGCGTGGAAGTTAGGGATGAGATC---CGCACTAATACTGAACCAA
Fson_PPT1_3k        GTAGTGCTTGTGACCACTT---------TGATACGCTT-TGTGTGCATATTTTAAACATG
Ftri_PPT1A_3k       AAAATGTTTTCATACACAT----------GATAAACTCACGTGCACTCAACCCAAGT-AA
Fram_PPT1A_4k       TATACTTTTGTGTTACCAT------TTTATATACTTTTGTGTCTCTTTTTTTTGTCTCAC
                           *        * *           **    *

Frob_PPT1_3.5k      ATTATACTGATTCTTAAAACATAAAAAAACAAATATTGGTACTGGTACCGATTGTGTCGA
Fson_PPT1_3k        ATTCATAGTA--------CCAACCACCTTCAATTTCTGTTATTCTAGCTAAACATGTGTA
Ftri_PPT1A_3k       ATTGATGTTGCACTT---CAACAAACAATTCAATTCATTCACCTAAGTGACCCGTGTATT
Fram_PPT1A_4k       ACGTATTGTATATTTGTACACCACACTATGCAATACAATAACTTAAG--------GCATT
                    *                       *      * *      *              *

Frob_PPT1_3.5k      TCGTGATGGTACTGATGGGGTTCAGTCGATGCCGAATTGGTGCTGTACCTGTGCTTGATA
Fson_PPT1_3k        AGGTGTT-----TCCTGTTGTAGTATACATGTAATTTTAAAGTTAGAATAGCCCTAAACA
Ftri_PPT1A_3k       TCCCATC-----TTATTTAGTTTTCTATGTATATGACTA----------TTTAATGCATA
Fram_PPT1A_4k       CTATATT-----ATATAAGGTTTTTTAT-TAAATTATTA----TGGGATCGTCTTTAATA
                                   *   **    *   *       *                * * *

Frob_PPT1_3.5k      AAA---------AAGAATGTCCATCCCTA--GTAGAAGCGG-----GTGAGAGGTATAGA
Fson_PPT1_3k        GCAATGTTAGTTAATTATAGTTTTGTGCAGATTAGAAGTTACCTTTTTGTTTCTTGTAGT
Ftri_PPT1A_3k       AAAGTTGGACACAAGAATTTTCCTATTTAATGTAG--------------TGAAGTCTAGA
Fram_PPT1A_4k       GTAAACCGAC--AAGAATTGTAGTA-GTACTGTAGTAGTATTTTTTTTATTAAATCGAAT
                      *         **  **     *    *   ***                   *  *

Frob_PPT1_3.5k      TAGATGAAA-----------------------ATACGGTATAGAGAAGTATAGACAGACA
Fson_PPT1_3k        TGATTCAGA------------------ATCTCATAAAGCAATGTAAGGTTTGATTCAGCA
Ftri_PPT1A_3k       CACATCCAA-----------ACACTAGTTTATAAATCGGATGAATAGATACATAGGGATA
Fram_PPT1A_4k       CGAATGGAATGGAATGGAGTGGAATATATTAAGAATCGAATCGGTGGGGGTGGTGGGGCG
                        *   *                         *  * *

Frob_PPT1_3.5k      GACTCGGAC---------------------------------------------------
Fson_PPT1_3k        ACTTTGTTT---------------------------------------------------
Ftri_PPT1A_3k       TCGTTATGC---------------------------------------------------
Fram_PPT1A_4k       GCGGTGGTCCGGCCGGTGGGGGTGGTGGGGCGGCGGTGGTCCGGCCGGTGGGGGTGGTGG


Frob_PPT1_3.5k      --------------------------------------------------------GCAA
Fson_PPT1_3k        -------------------------------------------------CTAGTTGGAAG
Ftri_PPT1A_3k       ------------------------------------------------------------
Fram_PPT1A_4k       GGCGGCGGTGGTCCGGCCGGTGGGGGGAGGTGGGGCGGCGGTGGTCCGGCCGGTGGGGGG


Frob_PPT1_3.5k      AGAAGAGATTTTCAT-------------------AACAGGTGATGGAGAGACA----AAA
Fson_PPT1_3k        AGAAGCTACTTC----------------------AGATGGTGGT----AGATGCTTGAAC
```

```
Ftri_PPT1A_3k       ACATGATGTACTCGA------------------TCAAGGCGATCCAAACATATTAGAA-
Fram_PPT1A_4k       AGGTGGGGCGGCCGATGGGGGGAGGTGGGGGAGGTGAGGGAGGTGAGAAGAGGTTAGAA-
                    *   *                                 ** * *    * *      **

Frob_PPT1_3.5k      TGAGAGCTCATGTGAAGATTGTTGAGGTATGTGGAGTTTT---TGCGGATGGTTTGACAT
Fson_PPT1_3k        CTGATTTTGATTTTAGATGGATGCAGTTAGTTACAGCTTCTACTGCGGCTGATA------
Ftri_PPT1A_3k       --------GTTTTTAAAACGATCAATGT-----TATTTTTTAAGATCAATCATACGTTGC
Fram_PPT1A_4k       --------GATCTCAGAA-----GACATAGGTCCACATCTTCTCCATGCAGACCTCCTGC
                              * * *         *  *      *  *

Frob_PPT1_3.5k      G-AAGATTCATGAACCATGATGGGTTGTCAATGCTTTAAAAAGAATAAAATTTTAGGGGT
Fson_PPT1_3k        A-AAGATATAATCTCTGTTA---------TACCTTTGAATTAGGGTT----------GGT
Ftri_PPT1A_3k       GTAATATTTTAAATTTATAG---------AAGGTTT----GAGGACC----------GAT
Fram_PPT1A_4k       A-GACATTTTTTA---ATGA---------AATGTCTTTCAGAAAACA----------AAC
                       * **          *            *    *     *

Frob_PPT1_3.5k      GGTTGTACGACATGAGAAACTC-------------------------------------A
Fson_PPT1_3k        AG--ATGCGCTATGCAT------------------------------------------G
Ftri_PPT1A_3k       AGTGTTGTACTAT-----------------------------------------------
Fram_PPT1A_4k       AGT-TTGCGCAATCAAAACCTCTTTCCGCGTCTGCGTGCGCGCAGATGAATAGGTGGGGA
                     *   *     **

Frob_PPT1_3.5k      AACAAACATATCAAGTCTTCGATTTGGT------------ACTTCGATGTTCA---TTAG
Fson_PPT1_3k        GATTAATATAT---------ACTTCAGT--------TGCAGAACCGTTGGAAATAATCAG
Ftri_PPT1A_3k       -ATTCATAGGTG--------GCCTTTGT----------------CTATGTTTAGTGTTCG
Fram_PPT1A_4k       AATGAAGAGGTGGGGAAGATGTTTTGGTAAAAAACAAACAACACCTATGTTTGGTGTTCG
                     *   * *  *            *  **                *  **       *  *

Frob_PPT1_3.5k      GTTGTAGAACATCTTTGATTTCTTCAAATTCTTTGTTCATCTTATTTGGAAAAGCACTA-
Fson_PPT1_3k        TTAATGCTAAT--GTAAACCATTTTG---CTTTTATGAATTATATTTGATAGCTGATGAT
Ftri_PPT1A_3k       TTTATAAGAGA--GTTGATTATTTTA---TCCTTTTGTATTGCATTT--------ATAAT
Fram_PPT1A_4k       TTTACAAAAGA--GTTGATTTTTTA----TCATTTTGTATTGCAGTT--------ATAAT
                     *      *     *  *    **        ** *  **   * **        *  *

Frob_PPT1_3.5k      ACATCATCATCGAAAATCTCATTTTTGGTTGAT---TAATAGGGTA--TTTAATCGTGTG
Fson_PPT1_3k        AGATTAGC--------TTCGAGGTTAAGAGACT---TGATAACCTACCCTAAACCTTAAA
Ftri_PPT1A_3k       ACATTTTT--------TTTCATATTATGTGAGTTTATTATAGTTTA--TTGACTACGGAA
Fram_PPT1A_4k       ACATTTCC---------TTCATATTATGTGAGTCTATTATAGTTTA--CTGACTACTGAA
                    * **                *  **  *    *   * ***   **   * *

Frob_PPT1_3.5k      TACATCTTCA-----------------CTCA------ATTGTTGTTCATTAATATTCAA
Fson_PPT1_3k        CACATAATGAACAAAAAGAAAGACGAATCTCACCCCAAAT---ACTTCATTTCTACCCGA
Ftri_PPT1A_3k       AACATCATAACCA---------------CTTACCACATGTCACGACTCATGGCCACCCAC
Fram_PPT1A_4k       AACATCATAACCA---------------CTTATCACATGTGGCGACTCATGGCCACTCAT
                     ****  * *                  ** *       *      ****    *  *

Frob_PPT1_3.5k      TTGGATTGTATGTCATCATTGTTTGTACATCTTCA------------CTCAATTGTTGTT
Fson_PPT1_3k        TGCAATTACAAGCTATTGAGAGTATGCGTATTTGA------AAGAGAAACAACT------
Ftri_PPT1A_3k       TGCATTTGCA--CTTTTGTCATGATGACAACTTCATAGTACAAGAGTCCCGGCCATCGTA
Fram_PPT1A_4k       TGCATTTGCA--TTTTTGTCATGATGACAATTTCACAGTACACGAGTCCCAACCATCGTA
                    *    **  *     *               ** *              *

Frob_PPT1_3.5k      CATCATCAT-------------TCAATCGACTATTCAATTG-------------------
Fson_PPT1_3k        ------------------------AATAAAGCGAGAAATTG-------------------
Ftri_PPT1A_3k       TATTTTAACATTTTGCGTCGTATCAATAGAATTTAACGTTGAGCGTTGTTAGTGTTCGCA
Fram_PPT1A_4k       TATTTTAATGTTTTGCGTCGTATCAATGGAGTTTAATGTTGAACGTTGTTAGCGTTCACA
                                            ***  *        ***

Frob_PPT1_3.5k      ----------------------GATTGTA------TGTTCAGAAGTGTATACGTATGTTG
Fson_PPT1_3k        --TTCTCCACTTTGAAACCATGGGTTTCACTTACTTTTTCTGATGT-AGTTTTCAAGTTC
Ftri_PPT1A_3k       CCTTCTCGCCTTTAGATAAATTTATAACA--------TCTTGAAGT-TGTTTTCACCTTG
Fram_PPT1A_4k       CCTTCTCG-CTTTAGATAAACTGATAACA--------TCTAGAAGT-TGTTTTCACCTTG
                                            *   *        *   ** **   *    *  **

Frob_PPT1_3.5k      GTGAGT-------ATGTATTAAAATTATAA------------------------------
Fson_PPT1_3k        TTGAGCTGCAG--AGGAGTCGTCATTGAAAT----CATCATCAACCCAATTTATTTTCCC
Ftri_PPT1A_3k       TTAATTTAGTGATAGGCGTTAACAATACAATATGGCATTAGTAATCAATTGTGCTTTTTT
Fram_PPT1A_4k       TTAATTTAGTGATATGCGTTAACAATATAATATGACGTCGGTAATCAACCATGTTTGTTT
                     * *         * *  *    *  *  **

Frob_PPT1_3.5k      ---------------------ACAAAAACAAAAAGTTAATGGATT---AACTGACTACTC
Fson_PPT1_3k        ACAGTTAACACTTGGTATATCAAAGAAGCCACAAAGCAATGCAAATCAAAGTAA--ACTC
Ftri_PPT1A_3k       GAGA--GACATTTTCCCTGATAGAGAGACATATTGGGGATGAAGTCTAAAATAATTACTT
Fram_PPT1A_4k       GAGA--GACATTTT-CCTGATAGAAAGACGTGTTGGGGATGAAGTCCAAAATAATT-CTT
                                         * * *  *         *** *     ** * *   **
```

```
Frob_PPT1_3.5k      TATCT----CTCAGGCAAAAATAAAATAAAATAA-AAACAGCATTTTACGGGATACTTCT
Fson_PPT1_3k        TAAAT----CAAAAGCAA----AACACATAAGAACACACAAAAATTGATTGAAAACTT--
Ftri_PPT1A_3k       TGGTTGTAGTTTATGCATTGTTAAAACATGATGATCCAAATCATCTAATAAAACACTTAT
Fram_PPT1A_4k       TAGTAGTAGTATATGTATTGTTAAAACATGATGATCCAAAACATCTAATAGAGCGATTCT
                    *           * * *     ** * *  *  *   * *  *  * *        **

Frob_PPT1_3.5k      TGTACACA----------AGTATGTA-TGTTTTAGGTAATAAGGCGAGTAT---------
Fson_PPT1_3k        ------------------ACGACTTCATGGTTTAAGAGAAAAGGGGTCTGTGAATCCCAA
Ftri_PPT1A_3k       TTTACGGGATGTCCTAGCACCATCCATTTTTTTGAGCAAAACATGGATTAC--------A
Fram_PPT1A_4k       TTTACGCGTTGTCCCACCACTACCCATTTATTTGAGCAAAACATGGATTAC--------A
                                       * *     *  ***  *  * *    *  *

Frob_PPT1_3.5k      ---------CATCGA------------ATATTATACT----AAAGCATTTTACGTTATTT
Fson_PPT1_3k        TACGAA---CGTCGA---------------TTACCCA--TGAACCCTCTTTACGAATT--
Ftri_PPT1A_3k       TTCGAACCATGTCGAGCTATTTTTGGAGTATTATTTTGTTTAAATCGCTTGCCAAATACG
Fram_PPT1A_4k       TTCGAACCATATCGTGTTAATTAAGGCGTATTATTTTGTTCAATTCATTTGCCAAA----
                               ***                ***        **  *  **  *

Frob_PPT1_3.5k      TTAGATAAAATCTCGGATTGAATTGTAGATTAAATTTAAATTTTGAAGTTTTTAAATTTT
Fson_PPT1_3k        ---GATGAGCGC-------AGATCGAGGACGAACCCTGAGCAAGAAATTGATTGAAAATT
Ftri_PPT1A_3k       TAAAGTAAAAAC-------AACTTTCAGACACCCTCAAATTAACATAAGACTAGAAAAAT
Fram_PPT1A_4k       ---GGTAAAAGC-------AACTTTCAGATGCTCTCAAGTTAACATAAGACTAGAAATTT
                         * *   *          *    **                 *    *  **   *

Frob_PPT1_3.5k      TATTTCATC---------CATTAATTTTT-------------------AAATTTTAATTTT
Fson_PPT1_3k        GATCAC-CCATGAACCATGTTACCTTCT-------------------------TCAATGG-
Ftri_PPT1A_3k       TGTTGTAAAAAATATCATATGATTTTTT----------------TAACATTTTTAATTA-
Fram_PPT1A_4k       TATTGGAACATGTACAAGATAAATTCTTATTAATGCTTGCTTAGAAACATATTTAATCCT
                      *                * *  *  *                        * ***

Frob_PPT1_3.5k      AGAATACTATCTAATTAAAAATTAAATACTGTTTAAATAGAATACACAAGATTCTTTTTA
Fson_PPT1_3k        ------ATGAGCAGTTGAACACAAA------TGATGAAAGGAAATAGAAGATGAGGCGGT
Ftri_PPT1A_3k       ATTATTACAAGTAACTAGATGTTGATGTATCTCACAATA-----TACATATTATTGAGAA
Fram_PPT1A_4k       GATATTATAAATCCTAGAAATCTAAT-----TTACGATAGAAATAACATGTTAATGTTAT
                                       *    *      *    * *      * *   *

Frob_PPT1_3.5k      A-AA---CTATATAGCTAATAAAATGGCAC------------------------------
Fson_PPT1_3k        G-GAAGGTTGGGCGGAGAATGCAA---CAC------------------------------
Ftri_PPT1A_3k       TAAAATTTGATA---------------CAC------------------------------
Fram_PPT1A_4k       ATAACTCTTAAGTAGTTCCCATAAAGACACTCCATATCTACAACAAAGACACTTTGTTCA
                       *                       ***

Frob_PPT1_3.5k      ------------GTGATAATTGT------------ATGCTATA-----AAGTTGTCTTGT
Fson_PPT1_3k        ------------GTATCACTTGT------------ATAGGAAATACCATAAACATCCTTG
Ftri_PPT1A_3k       ---------------AAAATCATTATCTTGAA---ATATAACACAACCAAACTGCCGTTA
Fram_PPT1A_4k       TTTACTTTAGCTTTCATAATTGTTTTATTGAATAAATATAATATATTTTAAGTGTTTATC
                                     * *  *            **   * *      *

Frob_PPT1_3.5k      AGTAACATAGTACTTA---AAATCTCATTACGTGCCTTAACCCCTTTCTTTTATTATATA
Fson_PPT1_3k        GTCACTGTAGCCACCGACCAAGAATAATAATATGCGTTTG----GTAGTT----AATATA
Ftri_PPT1A_3k       TTTTTTGTGATTGCTA---TGTAGTTATTTTTTGTGATTG----TTATTTTTACTATATA
Fram_PPT1A_4k       GTCATTCAAACACCAA---AAACATTACAATATGCATGTG----GTAATT----AGTATA
                                            * *     **           *  **      ****

Frob_PPT1_3.5k      TTG-AGACTAGCTTTAGTAAACCTGTGTATTACACGGGTTGAATAAAAAT----------
Fson_PPT1_3k        TTGTGAACCAGTTTTTAGAAACACGTGTAGTAAACGGG-TAAATGAAAATATTAAAGTAA
Ftri_PPT1A_3k       ATGTGGAATA-------TAA---TGTGGTCTCTACGAGTTG-------------------
Fram_PPT1A_4k       CTGTGAACTAGTTTTTAGAAAC-CGTG------------TGAATGAATATATTAAAGTAA
                     **   *  *         **   ***            *

Frob_PPT1_3.5k      --------TATTAA-----ATAGAATAAAAATTTATAATTTATAAATCGATATTATAGA-
Fson_PPT1_3k        TATTTATTTATTAATATTTAGAAAATAAAAAATAATTTTTTAAAATAAAATATGATCGAA
Ftri_PPT1A_3k       -----------TGATATCTAGTGATGAACGAGTTA-----------AAAATATTCTTGG-
Fram_PPT1A_4k       TATTTATCTATTAATATTTAAAAAAATAAAAAATAA--ATTTTGAGATAAATACGATTGAA
                               * *     *   *  **  * * *              ***   * *

Frob_PPT1_3.5k      ATAAGATATGATTGAAAATTTTCTTATAATAAATAATAAAAGTTTTATTTTAACACAAAA
Fson_PPT1_3k        ATTACGTATAATTAATTACTTTAGGTAATTAAACAATAATAATATTTTTTTCAAAC-TTT
Ftri_PPT1A_3k       TTGAGGGTTGGTGAA--AGTTTGGTAAGGGGGTTGGGGAAGGTCTTTTTATAGCGTATGT
Fram_PPT1A_4k       ATTAAGTATAATTAGTTACTTTAGGTAATTAAACAGTAACAATATTTTTTTAAAC--TTT
                     * *    *  *     * ***                *   * ** ** *

Frob_PPT1_3.5k      CGTTTTAAAAAATGTCATACTTAAAA----ACTTTCTTATTGTAA--TTTTCAA------
Fson_PPT1_3k        CGTCATTAAAAAAATTTTGATTAAAA----ATTTGTTTTTTTTAAATACTCCAAGTTT--
Ftri_PPT1A_3k       TTTTGTG------GTTTTATTTAATACATTAATTTCTTTTTGTAAAATTTTTAAAAGAGG
Fram_PPT1A_4k       CGTCATTAAAAAATTTTTTGCTTAAAA----ATTTGCTTTTT-TAAATACTCCAAGTTTTA
```

```
                      *  *        *  *  **** *    * **   ** ** ***    *  **

Frob_PPT1_3.5k      ------------------------------------------------------------
Fson_PPT1_3k        ------------------------------------------------------------
Ftri_PPT1A_3k       AAAATATAT---------------------------------------------------
Fram_PPT1A_4k       AAAAAATAATATTTTAAAAAAATGTTTTTTAAAGTTCTTTGTATAAAAATACTTCATATT


Frob_PPT1_3.5k      ---------------------------------TTTTAATTA--------------TTTT
Fson_PPT1_3k        ---------------------------------TTCTAAAAA-----------------T
Ftri_PPT1A_3k       ---------------------------------TAGTATATACGTGACATACATCTCTCT
Fram_PPT1A_4k       CTATAAAACTTTATTTTTTTAAAAAATTACAAATTTTAATAAAAAGAAACATATCTATCT
                                                     *  **   *                 *

Frob_PPT1_3.5k      TGTTTATTTAAAATTTA-------------------------------------------
Fson_PPT1_3k        ACTTTATTTAAAC-----------------------------------------------
Ftri_PPT1A_3k       ACTTTAATAAAATGATAAATGTGAGTGATACGTGTTAGCGTCACAATCATTCTCACCAAA
Fram_PPT1A_4k       ACTCTAATTAAAATGCATCTTGGGATGACACATGACAACACCATAATCATTCTCATCAAA
                      * ** * ***

Frob_PPT1_3.5k      ------------------------------------------------------------
Fson_PPT1_3k        ------------------------------------------------------------
Ftri_PPT1A_3k       AGTTTTTCGTCTCTCTTTCATATTAAATAATTATCTTAATGACTAATTATATTGTTTTAA
Fram_PPT1A_4k       ATTTTCCCGCCTCTCTTTCTTATTAAATAATTACCTTAATTGCTAATAATATTAATTAAC


Frob_PPT1_3.5k      --CTTTTTA------AAAATACCAATCATAAAATATTTTG--------TATTTTTAAAAA
Fson_PPT1_3k        -------------------------------AATGTTTTTTTAAAGCTCTTTGTATATA
Ftri_PPT1A_3k       TACATTTTAATATTTAATAAATTAAGAAAGAAATCTCTTTCTTAAGGTTATTTCTA-ATA
Fram_PPT1A_4k       ACCTTTTTA------GATTTATTAAGAAATAAATCTATTTATTAATGTTATTT-TAAATA
                                                   *** * **         * *** ** * *

Frob_PPT1_3.5k      ATTTGATGTTTTA-------------------------AAAAATTTTCT-----------
Fson_PPT1_3k        A-------------------------------------AAAAACTTTAT-----------
Ftri_PPT1A_3k       AATTGATATTATT-------------------------AAAAAGTCAATTTAAAAAAAAA
Fram_PPT1A_4k       AATAAAGTTTTTATAAAATAATAAATTTTTCTAAAGATAAAACTTTTTTTTAAAAATGAA
                    *                                     ****  *   *

Frob_PPT1_3.5k      --------------AATTTTATAAAAAGTTA-TTTCCTTAAAAATAATA-----------
Fson_PPT1_3k        ---------------ATTTCATAGAACTTTATTTTTTTTTAAAAATTACA-----------
Ftri_PPT1A_3k       AGTAATCAAATTTGGGAGTTGTAATAAGAAA--CTTTTTAAAAATAACA-----------
Fram_PPT1A_4k       ATATTTTAAGATTGGTATTTTTAAAAAGTCA--TTTTTTAAAAACAAAAAATAATCAATT
                                      *  **  *    *   *  *******  * *

Frob_PPT1_3.5k      ------------------TTAGTAAAAAGATCTATT------------------------
Fson_PPT1_3k        --------------TTTTTTAATAAAAAAACATGTT------------------------
Ftri_PPT1A_3k       -----TTTTATACGGTTTGTTTTAAAACAAAATGTT------------------------
Fram_PPT1A_4k       TTGAGAGTTATACGGTTTGTTTTAAAACAAAATGTTTATTTGTATTAGAATATCCTTTTA
                                       *  *****  *  * **

Frob_PPT1_3.5k      -----------------TCTTAATTTATT-------------------------------
Fson_PPT1_3k        -----------------T-TTAATATACC-------------------------------
Ftri_PPT1A_3k       -----------------------TATTTT-------------------------------
Fram_PPT1A_4k       CAATTAATAAATTTTTATATTAATATTTTATTTATTCAACCCGTGTAATACACGGTTTAC
                                           * *

Frob_PPT1_3.5k      -----------------------AAGAAATTATTT--TAATCTAACAAAT-TAAATAATA
Fson_PPT1_3k        -----------------------AAAAAATTGTTTTATAATTTGAAAAGTATAAATAATA
Ftri_PPT1A_3k       -----------------------TAAAAAT--------------AAAAATAAAAATAAAA
Fram_PPT1A_4k       TAAATCTAGTTTTTAATAAACCAAAAAAACTATTTTATAATTTGAAAAATATAAATAATA
                                            * ***               * ** *  ****** *

Frob_PPT1_3.5k      CTGTTAGTAATTAATGTGATTATTAAATAGGAGA--------------------------
Fson_PPT1_3k        TGATTAGCATTTAATGT-ATTATTAGATTATTA---------------------------
Ftri_PPT1A_3k       TTTTAAAAAGTTGATATCACTTTTGTATTATTA---------------------------
Fram_PPT1A_4k       TGATTAGCATTTAATGT-ATCATTAGATGGTTAACGCTAATGATCTCTTGCTTTAGTGGT
                       * *  * ** ** * *   **  **

Frob_PPT1_3.5k      -----------------------------------------------GAGAATCGAAAAA
Fson_PPT1_3k        ---------------------------------------------------------GAT
Ftri_PPT1A_3k       ------------------------------------------------------------
Fram_PPT1A_4k       TCAAGGGGAAGGTGGGAGGTTTTGCCTCTTTCGGATATCCAAAGTTCAAACCTTAGCATT


Frob_PPT1_3.5k      AATTAATGAGTAGTAACATGGTGTTAACAAGTGTCCCTCCTAGATATCTTTT--------
Fson_PPT1_3k        GGTTAATATTTATTC---------------------------------------------
```

```
Ftri_PPT1A_3k       ------------------------------------------------------------
Fram_PPT1A_4k       GGTGAATTATTACTAACTAGGTGTTGCTAACTTCTAACAACTAATAAGCCGTTCGAAAAA


Frob_PPT1_3.5k      -----------------------TATTAGA-----GTGTGGAGATTTTACGTGTTTACCT
Fson_PPT1_3k        -----------------------TAGTAATGACGCATGTTCAAATCCTAGTTG--GAGTT
Ftri_PPT1A_3k       -----------------------TAGTAAT--------------TTTTATACG--TAATT
Fram_PPT1A_4k       AAGAGAAAAATATTTTTAATCAATATTAATGACACGTGTT-AAATCCTAGGTG--TAGCT
                                           ** **                *  **   *   *  *

Frob_PPT1_3.5k      TTATTTTAAAATA----AAAAACATTATGATATGTG-----ATTGGTAGTTAATTTATTT
Fson_PPT1_3k        TAGGTGGATAGTGTAACAAAAATATCATGTGGTTTTTAACATTTTTGATTAATTAGTAT
Ftri_PPT1A_3k       TCAGTC---------------ATATCAT---------------------------------
Fram_PPT1A_4k       TCGGTGTACAATGCA--AAAAATATCATGTGACTTTTTAACATTTTTAATTAATTAGTAT
                    *   *                * ** **

Frob_PPT1_3.5k      TGACATACTAGATGTAATTCATGCATCCCACAATATACATATTAATGAGAGTAAACTTTG
Fson_PPT1_3k        AAATA-ACTAGACGTAATTCATGTATCTCACAATATACATATTATTGAGAATAAAAT---
Ftri_PPT1A_3k       ------------------------ATCTTAAAATAT-TATTTTATAATTAATAAA-----
Fram_PPT1A_4k       AAATA-ACTAGATGTAATTCATGTATCTCACAATATATATATTATTGAGAATAAAATTCG
                                            ***  * *****  ** ***     * ****

Frob_PPT1_3.5k      GTACAC-CAGTCAATATCTAGAAAATTTATTATAACACAACCAAAATGTATTT-------
Fson_PPT1_3k        ------------ATTATCTAGAAA------TATAACACAACCGAAATGTCGTTATTTTTG
Ftri_PPT1A_3k       ------------------------------------------------------------
Fram_PPT1A_4k       ATTCACAAAATCATTATCTAGAAA------TATAACACAACCAAAATGTCGTTATTTTTG


Frob_PPT1_3.5k      --------------------------------TTTTTCTTATATAA-ATAGAATAGGCCC
Fson_PPT1_3k        TGATTTTTATGTAGTTATTTT-----TTGCGATTTTTATTATATAATATGGAATAGGCCC
Ftri_PPT1A_3k       ----------------------------------TTTATTATATAA--------------
Fram_PPT1A_4k       TGATTGCTATGTAGTTGTTTTTGTGATTTTTATTTTTATTATATAATATGGAATAGGCCC
                                                      *** ********

Frob_PPT1_3.5k      GGTGGTCTCCACCGTGTTGTGAACTTGTGATATCTAGTGACGAAAGAGTTAAGAATTTTC
Fson_PPT1_3k        GGTGGTCTCCACCGAG--------TTGTGATATCTAGTGATGAAAGAGTTGAGAATATTC
Ftri_PPT1A_3k       ------------------------------------------------------ATATTT
Fram_PPT1A_4k       GGTGGTCTCCACCGAG--------TTGTGATATCTAGTGATGAAAGAGTTAAGAATATTC
                                                                          ** **

Frob_PPT1_3.5k      TAGGTTGAGGGTTGGTGAAAAGTTTGGTGGGGGGAGTTGGGGAAGGTCTTTTT-TAGCGT
Fson_PPT1_3k        TAGTTTGAGGGTTGGTG-AAAGTTTGGTAAGGGG-GTTGGGGAAGGTCTTTTTATAGCGT
Ftri_PPT1A_3k       TA----------------------------------------------------------
Fram_PPT1A_4k       TAGATTGAGGGTTGGTG-AAAGTTTGGTGAGGGG-GTTGGGGAAGGTCTTTTTATAGCGT
                    **

Frob_PPT1_3.5k      ATGTTTTTATTGGTTTTATTTAATGCATTAAATTTATTTTAT--ACATGTTAAAAGAGGA
Fson_PPT1_3k        ATGTTTTTGTTGGTTTTATTTAATACATTAATTTCTTTTTGTAAAAATTTTAAAAGAGGA
Ftri_PPT1A_3k       -----TTTATTCAACCCATGTAATACACGGGTTT----------------------ACAA
Fram_PPT1A_4k       ATGTTTTTGTTGGTTTTATTTAATACATTAATTTCTTTTTGTAAAAATGTTAAAAGAGGA
                         *** **      ** **** **      **                      *  *

Frob_PPT1_3.5k      AGATATATAAATATACGTGATACACATACCTAAATACAACTAAAATTCTAAAGGCATGAA
Fson_PPT1_3k        AGATATATTAATATACGTGATACACATACCTAATTACAACTTAAATTTTAAAGATTTAAT
Ftri_PPT1A_3k       AGCTA--------------------GTACATAAATAACACTTAAATTTTAAAGATTTGAT
Fram_PPT1A_4k       AAATATATTAGTATACTTGATACACATACCTAAATACAACTTAAATTTTAAAGATTTGAT
                    *  **                     *** *** **  *** ***** *****   * *

Frob_PPT1_3.5k      TGTAAAAATTGAAATTTATAATAAAGCAAATATAATTACCTAAAATAG-ATTTTTTTTC
Fson_PPT1_3k        TGAATAAATTGAAATTTATTAC-AAGTAAATATAATTACCTAAAATAGCAATTCCATTTC
Ftri_PPT1A_3k       TGAATAAATTGAAATTTATTAC-AAGTAACTATAATTACCTAAAATAGCAATTCCATTTC
Fram_PPT1A_4k       TGAATAAATTGAAATTTATTAC-AAGTAACTATAATTACCTAAAATAGCAATTCCATTTC
                    ** * ************** *  *** ** ****************** * **   ****

Frob_PPT1_3.5k      AAATTTGTTACATTTTAGTAAAATACTAGCTTTAGTAACCCATGTGTTGCACGGATTAAA
Fson_PPT1_3k        AAATTTGTTACATTT---------------------------------------------
Ftri_PPT1A_3k       AAATTTGTTACATTT---------------------------------------------
Fram_PPT1A_4k       AAATTTGTTACATTT---------------------------------------------
                    ***************

Frob_PPT1_3.5k      TAAATAAAATATTAAATAAAATAAAACTTTATTAATTATAAAATAATATTTTAAGATAAT
Fson_PPT1_3k        ------------------------------------------------------------
Ftri_PPT1A_3k       ------------------------------------------------------------
Fram_PPT1A_4k       ------------------------------------------------------------
```

```
Frob_PPT1_3.5k      ATATAATTGAAATTACGTATTAGTAAGAATATAAGTAACAAAACAATGATATTAACTTTT
Fson_PPT1_3k        -------------------TTAGTACTA--------------------------------
Ftri_PPT1A_3k       -------------------TTAGTAAAA--------------------------------
Fram_PPT1A_4k       -------------------TTAGTAAAA--------------------------------
                                       ******  *

Frob_PPT1_3.5k      TAAATTTTTTGTTTGAAAAAAACTAATCATTTTATTTCAAAACAAACCATTTTAAAAAAA
Fson_PPT1_3k        ------------------------------------------------------------
Ftri_PPT1A_3k       ------------------------------------------------------------
Fram_PPT1A_4k       ------------------------------------------------------------


Frob_PPT1_3.5k      TTTCATTATAATAATTTTAATTATTTTTTATTTATAAATATTTTAATTTTTAAAAACATT
Fson_PPT1_3k        ------------------------------------------------------------
Ftri_PPT1A_3k       ------------------------------------------------------------
Fram_PPT1A_4k       ------------------------------------------------------------


Frob_PPT1_3.5k      AATTTTAAAATATTTTAATGTTAAAAAAATTATTTTATAAAAAAATATTTTTATAAATAA
Fson_PPT1_3k        ------------------------------------------------------------
Ftri_PPT1A_3k       ------------------------------------------------------------
Fram_PPT1A_4k       ------------------------------------------------------------


Frob_PPT1_3.5k      CATTAACAAAAAGAATGTTTATTTAATTTATTAAAAGTATTTGTGGTTTATAAAAAATAA
Fson_PPT1_3k        ----------------------------TATTAAAAG-----------------------
Ftri_PPT1A_3k       ----------------------------TATTTAAAG-----------------------
Fram_PPT1A_4k       ----------------------------TATTAAAAG-----------------------
                                                **** ****

Frob_PPT1_3.5k      ATAATATTATTTGTAATTATAGTAACTATTAATTAGAAAAGATATGCAGGAAAATTTTGA
Fson_PPT1_3k        ----------TTGTACT-------------------------------------------
Ftri_PPT1A_3k       ----------TTGCAGT-------------------------------------------
Fram_PPT1A_4k       ----------TTGTAGT-------------------------------------------
                              *** * *

Frob_PPT1_3.5k      TAAGAAGTAATGTGGTGCTGCCACGTATCCACTTTATTTAATATTTTATTAGAGTAGAGA
Fson_PPT1_3k        ------------------------------------------------------------
Ftri_PPT1A_3k       ------------------------------------------------------------
Fram_PPT1A_4k       ------------------------------------------------------------


Frob_PPT1_3.5k      GATATTAAAAGTTGTAGTATGAAGAGGTTTTCAAATATCATTTACAAACCGATTACACCG
Fson_PPT1_3k        -----------CTGCACTCCATAGAAATTTTCAATTTTCAATTACAAACCGATTACACCA
Ftri_PPT1A_3k       -----------TTGTATTCTGTAAA-------AAATTTCAATTACAAACCAATTAAACCA
Fram_PPT1A_4k       -----------CTGTACTCTGTAAA-------AATTTTCAATTACAAA--------ACCG
                                ** * *    * *       ** * *** *******        ***

Frob_PPT1_3.5k      AAGACCGAATTTAACCAAAATTATCTTTCAACCGTACCGATTTAATTACTCATCTAAAAT
Fson_PPT1_3k        AAAATCAAATTAAACCAAACCTGTATTTCAGCCGTACCGAATTAATCACTCAATTAAAAA
Ftri_PPT1A_3k       AAAATC-AACTAAATCAAAATTGTGTTTCACCCATTTCAAATTTGTTACTCAGTTAAGAA
Fram_PPT1A_4k       AAAATCGAATTAAGTCAAAATTGTGTTTCACCCGTCCCAAATTTGTTACTCAGTTAAAAA
                    ** * * ** * *  ****  * * ***** ** *  * * **  * *****  *** *

Frob_PPT1_3.5k      TTTACTAATTTAAAGTTTTTAAATATTAACTTAAAGTGAGAAAACTTTAAATTACTCTAG
Fson_PPT1_3k        CTT--CAATTTG------------------TTAAACCGAGAAAACTTTATATTACTTTAG
Ftri_PPT1A_3k       CTT--CAATTTG------------------TTAAACTGAAAAAACCTTACATTACTCTAG
Fram_PPT1A_4k       CTT--TAATTTG------------------TTAAACTGAAAAAACCTTACATTACTCTAG
                     **   *****                   *****  ** ***** *** ****** ***

Frob_PPT1_3.5k      T-AAAATATTTAAA-AACTTGATTGCGGTCATACGACCAGGCTATGAACCTTTT--ATTT
Fson_PPT1_3k        TAAAAACATTTAAAAAACTTGATTACAACTAGATGATCTGTCTATGAACTTATTAACTTT
Ftri_PPT1A_3k       T-AAAACATTTAAAAAACTTGATTACAATAAAATGACCACTCTAAGAATATTTTTTATTT
Fram_PPT1A_4k       T-AAAACATTTAAAGAACTTGATTACAATAAGATGAATAGGCTATGAATTTTTTTTATTT
                    * **** ******* ********* *    * * **     *** ***  * **   ***

Frob_PPT1_3.5k      TCTTGTACAAATCGCTAAAAATTTAGTCCTTATA---AAATCAACGAAGCCAAGCTAAT-
Fson_PPT1_3k        TTTTGTACAACCTGTT--------------------------------------------
Ftri_PPT1A_3k       TTCTGTACACCTCGTTAAAAGTTTAGTCCTTTAACCGAGACCGAGTAAACCAAACTGATA
Fram_PPT1A_4k       TTTTCTACAACCCGTTAAAAATTTAGTCCTTTAACAGAAATCGAGGAAACCAAGCTTAT-
                    *  * ****    * *

Frob_PPT1_3.5k      AAAATCCAAAAACCAGAAACCCAAACTAATAAATGCCCAAAACAATTAAGTATTTTTTTA
Fson_PPT1_3k        AAAATCCAAAA-CCCGAAACCCAAACCAGTAAACACCCAAAACAACAAAATTGGTTTTTA
Ftri_PPT1A_3k       AAAATCCAAAACCCCGAAA----------------CCCAAAACAATCTAGTTGTTTTTCA
Fram_PPT1A_4k       AAAATCCAAAACCCCGAAACCCAAACCAATAAACACCCAAAATAATCAAGTTGTTTTTCA
```

```
                    *********** ** ****                 ******* **   * *   **** *

Frob_PPT1_3.5k      AACGACGACTC-ACGTGCTCATCAAATCTACTCTCATACTTTACTCCATAGTCTACAACA
Fson_PPT1_3k        AACAACAACTC-ACACTTTCATCAAACCTACTCTCATACTTTACCG-------GACAATA
Ftri_PPT1A_3k       ATCA--------ACACGCTCATCAAACCTACGATCATGTTTTACCTCACAGTCGACAGTA
Fram_PPT1A_4k       ATCAACAACTCAACACACTCATCAAACCTACGATCATACTTTACCTCACAGTCCACAGTA
                    * *         **    ******** ****  ****  *****          ***  *

Frob_PPT1_3.5k      AAACTCGAACCCACCACGTATAACAAAGAT----ATATCAATTAC-ACTTCAAAATACCA
Fson_PPT1_3k        AAACTCGAACCGACAACCTGGAAGAAAGATAG--ATATCATTTACAACTTCAAAATACCA
Ftri_PPT1A_3k       AAACTCGAACCTACAACCCCGAAGAAACATATGCATATCATTTACAAGTTGAAAATAGCA
Fram_PPT1A_4k       AAACTCGAACCACCAACCTCGAAGAAACAT----ATA-----------------------
                    ***********  * **    ** *** **    ***

Frob_PPT1_3.5k      CTAGAC---TATAATTCTTAGGTTGGTAAGGCCAAATCTGATATCCCAAGAATAT-CCCA
Fson_PPT1_3k        CTAGAC---TATAATTTTTAGGTTGGTGAGGCCAAATCTGATATCCCAAGAATATCCCC-
Ftri_PPT1A_3k       CAAGACTATTATAATTGCTAGGTTGGTGAGTCCAAATCTGATATCCCAAGAATATCCCCA
Fram_PPT1A_4k       --------------------------------CATATATGATATCCCAAGAATATCCCCA
                                                    ** ** ***************** ***

Frob_PPT1_3.5k      ATATAAAAAACACCGGAAAAACGATAT-AAAACTTAACAAAAAACCA-------------
Fson_PPT1_3k        ATATAAAAAACACCGT-AAAACGATAT-AAAACTAAACAAAAACCCA-------------
Ftri_PPT1A_3k       ATATAAAAAACACCGAAAAAACGATAT-AAAACAAAGCAAAACCCCAACACAAATTCAGT
Fram_PPT1A_4k       ATATAAAAAACACCGAAAAAACGATATAAAAACTAAACAAAACCCCAA-ACAAATTCACT
                    ***************  ********** *****  * *****  ***

Frob_PPT1_3.5k      ------------------------------------------------------------
Fson_PPT1_3k        ------------------------------------------------------------
Ftri_PPT1A_3k       CTTCACTGCATAGGCGGAGCCTCCAAGACACCTTCTTCGTCTACGGTATCTACCTACTCC
Fram_PPT1A_4k       CTTCACTGCATAGGCGGAGCCTCCAAGACACCTTCTTCATCTACGGTATCTACCTACTCC


Frob_PPT1_3.5k      ----------------------AACA
Fson_PPT1_3k        --------------------------
Ftri_PPT1A_3k       GAGCATCAATCGGAGATCTCACAATA
Fram_PPT1A_4k       GATCATCAATCGGAGATCTCACAATA


#########PPT2 from four species


Fson_PPT2_3k      ------TAGCTATATAATTGCTA-------------------------------------
Ftri_PPT2_3k      -------------ATAAATGCCC-------------------------------------
Frob_PPT2_3k      ------------------------------------------------------------
Fram_PPT2_3k      ACCCATTCCTTCATCAAACGCCCAACCCACCCACTCGGCCCAGCCTGATGTTGTCCCGGC


Fson_PPT2_3k      ------------------------------------------------------------
Ftri_PPT2_3k      ------------------------------------------------------------
Frob_PPT2_3k      ------------------------------------------------------------
Fram_PPT2_3k      CCAACAGACCTCTACACCACCTATCCACCACCAAGTCCCTCCCCCATCTCCAGCCACCAA


Fson_PPT2_3k      ------------TGATGATAAAAAAAGATTTAAGCCTGAATTTCGAAATTACATTCATTT
Ftri_PPT2_3k      ------------------------------------------------------------
Frob_PPT2_3k      ------------------------------------------------------------
Fram_PPT2_3k      CACCCCATAATTTTATTTTAAAAGGATCTTAAAGTCAATTAAAGAATTTTACAAAAAAAA


Fson_PPT2_3k      GTAAAAACGCATAAAATATTTGGCAACTTGAATGGACTATATACT----CTTGCAAGATT
Ftri_PPT2_3k      ------------------------------------------------------------
Frob_PPT2_3k      ------------------------------------------------------------
Fram_PPT2_3k      AGAAAGAAAAGAAAGAAAGAAACGTAAGTGCATAGGCTCTATTTTAAGCTTCATGTGGAT


Fson_PPT2_3k      GGTAGTGAAAAAAAACGTTTTTCAATTACTTTGGTGTATCATGGATTGAAGTTATT----
Ftri_PPT2_3k      ------------------------------------------------------------
Frob_PPT2_3k      ------------------------------------------------------------
Fram_PPT2_3k      GATGTCATGAAAACATTTGCATTATCCCTTAAAATTTGTTATGTGATGTACTTGTTTGAT


Fson_PPT2_3k      -------------------------ATAAGTTCTAATGGTCGCAACAAGTAGTAATAGAT
Ftri_PPT2_3k      ------------------------------------------------------------
Frob_PPT2_3k      ------------------------------------------------------------
Fram_PPT2_3k      ATGAATGGAGATTGACTATGTGTTAAGTAATTTGAGTAGGTGTGATAATTTTTCAGAG--
```

```
Fson_PPT2_3k      GTTTTCATTTAATGAAGTATGTGAAGCCGAATTACATAACCAAATTGGTGGTCAATATTT
Ftri_PPT2_3k      ------------------------------------------------------------
Frob_PPT2_3k      ------------------------------------------------------------
Fram_PPT2_3k      ---TTTAATATAACAAATATGTTAAAGACAGTTAAGTATATAACTAATCTTTCTCTTTTT

Fson_PPT2_3k      GAAT---GATGATTGCATTTGTTACATAAAAATAAATTGCTTAAGAATGTTTCTATCAAA
Ftri_PPT2_3k      ------------------------------------------------------------
Frob_PPT2_3k      ------------------------------------------------------------
Fram_PPT2_3k      GTTTCTGGTTGATGTGCTCTTTCATGAGACGGACCGTTTGTTCCATATGTTGTTATCTAT

Fson_PPT2_3k      GATGTAATAGATAGTTTTGAAAATA-----------------------------------
Ftri_PPT2_3k      ------------------------------------------------------------
Frob_PPT2_3k      ------------------------------------------------------------
Fram_PPT2_3k      CCTCGAATTTATATTTCTTGGTATGATTCAATTCGGCTAGGTTTTAGTAGTGGTTCATTG

Fson_PPT2_3k      ----------------------------------------------------TGAAGACT
Ftri_PPT2_3k      ------------------------------------------------------------
Frob_PPT2_3k      ------------------------------------------------------------
Fram_PPT2_3k      CACTATGGTTGTGAGGTAATTCCTCCTTCTTTCATTATGGTCTTTTCGGCTCTTCAAAAT

Fson_PPT2_3k      TCCAGAGAACAAGTGCAATAAATAGTGTGCATCCGTTGAAGATGTTCAAGCACTTGACTT
Ftri_PPT2_3k      ----------------------TCATGTGCA----------------AGGCACATGA---
Frob_PPT2_3k      ------------------------------------------------------------
Fram_PPT2_3k      TAAACCCTCTTCTTGGTCTAGTTATTGTGCATCCGTTGAAGCTGTGCGAGCACTTGACTT

Fson_PPT2_3k      GGATTTTCTTGATTTTCTGGTCGGTTCACTTTATCGTCATTGGTGGGGCATGCTATTTGG
Ftri_PPT2_3k      -------TTGGATTTAACGGAAAATTTTAACGTCCGTTACCGAAAAGG------------
Frob_PPT2_3k      ------------------------------------------------------------
Fram_PPT2_3k      AGACTTTCTTGATTTTCTGGTCAGTTGGCTTTCGCGTCATTGGTGGGGCATGTTGTTT--

Fson_PPT2_3k      GTTATGGATTTCTAGAAGAAACGATTGTGATAATGAGAATTCAATAAACGGTTGGGGATG
Ftri_PPT2_3k      ------------------------------------------------------------
Frob_PPT2_3k      ------------------------------------------------------------
Fram_PPT2_3k      ------------------------------------------------------GGGGTG

Fson_PPT2_3k      GTAGTGAAATTGTAAAGACGAAAAGATGAGCGTTTGAGATTGGTAGTCAAGTTACAGCGC
Ftri_PPT2_3k      --ACTGAACGTGTAACAACATTTAGACG--------------------------------
Frob_PPT2_3k      ------------------------------------------------------------
Fram_PPT2_3k      ATAGTGAAATTGTAAAGACCAAAATATGAGCGTTGGAGATTGGTAGTCAAGTTATAGTGG

Fson_PPT2_3k      CGAAAATATCGACTAGTTATTGCTCTTATTTCTACGCCATCTAGGCTAGTTAGAGTATCT
Ftri_PPT2_3k      ------TAAAGGACAATTATTGTAATTTCCT---------------TCGTTTAAGGACAA
Frob_PPT2_3k      ------------------------------------------------------------
Fram_PPT2_3k      CGAAAACATCGACTGGTTACTGCTCTTACTTTTATGGCATCTAGGCTCGTTAGAGGATCT

Fson_PPT2_3k      CG-GAGTCATTTTCTAGCAATTAAGAGAGGTAGAATAGAAAATCCATTATGAAAAATTAT
Ftri_PPT2_3k      TATTTGAAATTTGCTAT--------------------AAAGATAAATGATGAAAAAT---
Frob_PPT2_3k      ------------------------------------------------------------
Fram_PPT2_3k      CGTAAGCCATTTTCTAGCAATTGAGGGAGGTAAAATGGAAAATCCATTATGAAAAATTGT

Fson_PPT2_3k      CTTGATTATTGACTATTTGTAGGTTTTGGATTTTTTTTTTTTTAAAG--GTGAAAGATCT
Ftri_PPT2_3k      ------------------------------GTAATTTACTTTAAAAAATGTGAAATATCT
Frob_PPT2_3k      ------------------------------------------------------------
Fram_PPT2_3k      CTTGATTATTATGTATTTATAGGTTTTGGGGTTTTTTGAAAAAAAAAATGTGAAAGATCT

Fson_PPT2_3k      ATTA-GGCGGTCACCATTTGACCGTTCATGAACGGTAACCAAGGCAGTGGGTTGTCGTTG
Ftri_PPT2_3k      GTAAGGGCAGCCACCATTTGACCTTTCATGAACGGTAACTAAGGTAGTGGGTTGTCGTTG
Frob_PPT2_3k      ------------------------------------------------------------
Fram_PPT2_3k      GTTAGGGCGGCCACCATTTGACCTTTCATGAACGGTAACTAAGGCAGTGGGTTTTCGTTG

Fson_PPT2_3k      GTACTCGTCTCTCGCCAAAACCACCGCTAGTGATGCAC----------------------
Ftri_PPT2_3k      GTACCCATCTTTCATCAACATCCCCATCAACAGAGATAGAGCTTGGATATTTTCTTAAGG
```

```
Frob_PPT2_3k      ------------------------------------------------------------
Fram_PPT2_3k      GTACCCATCTCTCGTCAACGTCC-------------------------------------


Fson_PPT2_3k      ------------------------------------------------------------
Ftri_PPT2_3k      GGACGGGAGGATGTAATTAAATTTTTTTAGGGGGTGAAATCGTAATATAGAAAAAAAGTT
Frob_PPT2_3k      ------------------------------------------------------------
Fram_PPT2_3k      ------------------------------------------------------------


Fson_PPT2_3k      ------------------------------------------------------------
Ftri_PPT2_3k      ACACTACTAGACCAAAAATTAATAATACTGACGAAAAAAAGAAGAGCGGGGGGCCCTCCC
Frob_PPT2_3k      ------------------------------------------------------------
Fram_PPT2_3k      ------------------------------------------------------------


Fson_PPT2_3k      -----------------------CCCATCAGCAACCAAGAG-TGACATCCTCTCCTTCTA
Ftri_PPT2_3k      AGCCCTTTAATGCTTAGTCCATGCCCATCAACAACCAATAGGTGACGTCCTCTCCTTTTA
Frob_PPT2_3k      ------------------------------------------TGACGTCCTCTCCTTCTG
Fram_PPT2_3k      -----------------------CCCATCAGCAACCAAGAGGTGACGTCCTCTCCTTCTG
                                                            **** ********** *

Fson_PPT2_3k      GATGCCATGACAACAGCCTCCTCTAACGTGAAACAC-TAAAATAGTAAAGTA--------
Ftri_PPT2_3k      GATGCCACGAACACAAGCTTTCTTAACGTGAAATACAAAAAATAGTAAAGTA--------
Frob_PPT2_3k      GATGCCATGACCAC-----CCCCTAACGTGAAAAAC-TAAAATAGTAAAATACCATATAT
Fram_PPT2_3k      GATGCCATAACCACAACC-TCCCCAACGTGAAAAAC-TAAAATTGTAAAATA--------
                  *******  *  **          ********* **  ***** ***** **

Fson_PPT2_3k      ACATGTATATATTATC--TAATAAGTACTAACATATTTTGTATCTA-GTCATATTTTATT
Ftri_PPT2_3k      ACATGTATATATTATCTATACTAAGTACTTAGATATTTTCTATCTAGGTTTTATTTTATT
Frob_PPT2_3k      ATATATATATATAATC--TACTAAGTACCTACCTATTTTGTATCTA-GTTATTTTTTATT
Fram_PPT2_3k      ACATGTATATAT-ATC--TAACAAGTACTAAGATATTTTGTATCTG-GTTTT-TTTTATT
                  * ** ******* ***  **  ******  *  ****** *****  **  * *******

Fson_PPT2_3k      GACCCATGTCTTTGGA----TATTGTACATTAGTGTGTACACTTTTCTTTGTTAATAATA
Ftri_PPT2_3k      GACCCGTGTCTTTGGA----TATTGTACATTATAGTGTACACTTTTCTTTGTTAAAAAAA
Frob_PPT2_3k      GACACACGTCTTTGGATATTTATTGTACATTATAGTGTACACATTTCTTTGTTAATAAAA
Fram_PPT2_3k      GACCCATGTCTTTGGA----TATTGTACATTATAGTGTACACTTTTCTTTGTTAATAAAA
                  *** *  *********    ************  ******** ************ ** *

Fson_PPT2_3k      CATGTGTAAACTTTTTATAACATTCTTTATATTTAAAATAATATTAGTATCCAAATATAA
Ftri_PPT2_3k      CATGGGT-AACTTTTTATAACATTCTGTATATTTAAAATTATATT--TATCCAAATATAA
Frob_PPT2_3k      CATGTGT-AACTTTTTATAACATTCTTTATATTTATAATAATATTGGTGTCCAAATATAA
Fram_PPT2_3k      CATGGGT-AACTTTTCATAACATTCTATATATTTAAAATTATATTAGTTTCCAAATATAA
                  **** ** ******* ********** ******** *** *****  * ***********

Fson_PPT2_3k      TCATGACA-GTGTAAAACTAT-AAAGGAAGGTTGTCCATGTACTATTTATCCGCAATCAA
Ftri_PPT2_3k      TCATAACA-TTGTAAAACTACAAAAGGAAGG-TGTCCACAAGCTATTCATCCGGAATCAA
Frob_PPT2_3k      TCATAACATTTGTAAAACTAC-AAAGGAAGGTTGTCCACATACTATTCATCCGCAATCAA
Fram_PPT2_3k      TCATAACA-TTGTAAAACTACAAAAGGAAGGTTGTCCACAAGCTATTCATCCGGAATCAA
                  **** ***  **********  ********* ******    ***** ***** ******

Fson_PPT2_3k      AGGCTCAACTTAATTGAGAATTCTGGACCACAAACATACCAAGGACCAAACAATTTGCCT
Ftri_PPT2_3k      AGGCTCAACTTAATTGGGAATTCTGGACCACAAACATACCAAGGACCAAACAATTTGCCT
Frob_PPT2_3k      AGGCTCAACTTATTTGAGAATTCTGGACCACAAACATACCAAGGACCAAACAATTTGCTT
Fram_PPT2_3k      AGGCTCAACTTAATTGAGAATTTTGGACCACAAACATACCAAGGACCAAACAATTTGCCT
                  ************ *** ***** *********************************** *

Fson_PPT2_3k      CACACTTATTGGTCAATAGGCTCACTCACGTGCATTGCACGTGACTTTGGTTACTCCCCA
Ftri_PPT2_3k      CACACTTATTGGTCAATAGGCTCACTCACGTGCATTGCACGTGACTTTGGTTACTCCACA
Frob_PPT2_3k      CACACTTATTGGTCAATAGGGTCACTCACGTGCATTGCACGTGACTTTGATTACTCCCCA
Fram_PPT2_3k      CACACTTATTGGTCAATAGGCTCACTCACGTGCATTGCACGTGACTTTGGTTACTCCACA
                  ******************** **************************** ******* **

Fson_PPT2_3k      TGGAACTAGCATGCCTCTAGAGTGTTGCATCCTATTAAATCTTTTACCCAAACACCTTTT
Ftri_PPT2_3k      TGGAACTAGCATGCTTCTAGAGTGTTGCATCCTATTAAATCTTTTACCCAAACACCTTTT
Frob_PPT2_3k      TAGAACTAGCATGCCTCTAGAGTGTTGCATCCTATTAAATCTTTTACCCAAACACCTTTT
Fram_PPT2_3k      TGGAACTAGCATGCTTCTAGAGTGTTGCATCCTATTAAATCTTTTACTCAAACACCTTTT
                  * ************ ******************************** ************

Fson_PPT2_3k      AAGTTGCAAAAAGTGTGTTATTGGTA-------TAAATTGTTGTTG-AAAGATTTA-GCA
Ftri_PPT2_3k      AAGTTGCAAAAAGTGTGTTA-------------TATATTGTTGTTGAAAAGATTTTGGGA
Frob_PPT2_3k      AAGTGGCAAAAAATGTGTTAATGGTATAAATTCTAAATTCTTGTTGTAAAAGACTTAGCA
Fram_PPT2_3k      AAGTTGCAAAAAGTGTGTTATTGGTA-------TAAATTGTTGTTGAAAAGATTTAGGAA
                  **** ******* *******             ** *** ****** ***    *  * *
```

```
Fson_PPT2_3k      ATGTGTTGAAGGAGATAGAAAGAATTAATTCTTAATAAATTTTAGAATTCTAATTTCAAA
Ftri_PPT2_3k      ACATGTTTAAGGAGATA-AAAGAATCAATCTTTATATAATTCCAAAATTGTAA-TTCAAA
Frob_PPT2_3k      ATATGTTTAAGGAGATAAAAATA--TGATTCTTATAAAATTCTAAAATTATAT-TCCAAA
Fram_PPT2_3k      ATATGTTTAAGGAGATA-AAAGAATTAATCTTTATAGAATTCCAAAATTGTAA-TTCAAA
                  *  **** ********* *** *    **  ***   ****  * **** **  * ****

Fson_PPT2_3k      CATTTATTAGTTGATTACTGAATTTTACAAACTATAATTAACT-TTTTATGTCATTTTAT
Ftri_PPT2_3k      CATTTTGTAGTTGATTATTGAATTTAGCAAATTATAATTAACT-ATTTATGCTTTTTTAT
Frob_PPT2_3k      CCTCTATTAGTTGATCACTCAATTTAACAAATTATAATTAACTCTTTTATGTCTTTTTAT
Fram_PPT2_3k      CATTTTGTAGTTGATTATTGAATTTAGCAAATTATAATTAACT-ATTTATGT-TTTTTAT
                  * * *  ******** * * *****  **** ***********  ******   ******

Fson_PPT2_3k      TGTATGTAATTATGACTAAACTAAAATGAAAAAGATGTAAGAAAAGGTTAAAAGTGTTAA
Ftri_PPT2_3k      TGTATGTGATTATACATAAACTAAAATGAAAAAGATGTAAGAAAAGGTAAAAACTAATAA
Frob_PPT2_3k      TGCATGTAATTATAATTGAACTAAGGTGAAGTA-ATGTAAGAAGAGGTTAAAAGTGTCAA
Fram_PPT2_3k      TGTATGTGATTATAAATAAACTAAAATGAAAAACTTGTAAGAAAAGGT------------
                  ** **** *****   * ******  ****  *  ******** ****

Fson_PPT2_3k      GAGCATT-----------------------------------------------------
Ftri_PPT2_3k      GAGCATTCACAACCTATTCTTCATCTTTATTCATATAATTACACTAAAAATCACTACTTT
Frob_PPT2_3k      GAGCTTC-----------------------------------------------------
Fram_PPT2_3k      ------------------------------------------------------------


Fson_PPT2_3k      ------------------------------------------------------------
Ftri_PPT2_3k      TTCTCTCTTATTTTTAACTTTATCATTACTTTTTCTCTCTCATTCACTCACAACCACTTA
Frob_PPT2_3k      ------------------------------------------------------------
Fram_PPT2_3k      ------------------------------------------------------------


Fson_PPT2_3k      ------------------------------------------------------------
Ftri_PPT2_3k      AAATATATTAAAACCATTATATGATTTTGAATAGTGATTCCCCAAAATATAAATTGAACA
Frob_PPT2_3k      ------------------------------------------------------------
Fram_PPT2_3k      ------------------------------------------------------------


Fson_PPT2_3k      ------------------------------------------------------------
Ftri_PPT2_3k      GTAAATTTACCATAGCTTTCACTCACAACTACTTATAACCATACCATATAATATAAGAAA
Frob_PPT2_3k      ------------------------------------------------------------
Fram_PPT2_3k      ------------------------------------------------------------


Fson_PPT2_3k      -------------------------------------------AACAGATAAAAAAACAT
Ftri_PPT2_3k      TACACACATATAAATATAAGGAACCGATTATAAATGCTCTAAAAGCAAGGTAAAAAGCAT
Frob_PPT2_3k      -------------------------------------------AACAAGGTGAAAAACGT
Fram_PPT2_3k      ---------------------------------------------------AAAAAGCAT
                                                                      **** * *

Fson_PPT2_3k      CAAAGATCTTTATGTCAGCTTTCT-ACTAGCTATACCACATATGACTCTCTTATCCTTTC
Ftri_PPT2_3k      GAGGGACCTCTATGTCAGCTTTCCCATTGGCTCTATC----------------CTCTCTC
Frob_PPT2_3k      CAAGGACCTCTATGTCAGCTTTCTCCCTAGCTTTATCACATTCAACTACTATTTTCTTTC
Fram_PPT2_3k      CAGGGACCTCTATGTCAGCTTTCCCATTGGCTCTATC-----------------------
                   *  ** ** *************    * *** ** *

Fson_PPT2_3k      TTTC------TATCCTCTCTCTTACGGTCTCTCACTTCCT----------------TTGT
Ftri_PPT2_3k      TCTCTTACGGTCTCTCACTTCTTAATGTTATTTTCTTTTTAAATTAATTTCAATTTTTCT
Frob_PPT2_3k      TTTCTTTTGGTCATCTCTTTCTTGCAATCTTTTTTTTTTT------------TTTTTTGT
Fram_PPT2_3k      ----------------------------------CTTATT----------------TTGT
                                                     **  *                ** *

Fson_PPT2_3k      TCGTGGTTGTCCATAGCTATAGATAACCCTCCCCC---------------CCGCCGCTCC
Ftri_PPT2_3k      TCCAAATGTTTAATAAAAATTATTATTTTCTTATAAAATATTAAAAATATCAGTTTATCT
Frob_PPT2_3k      TCATGGTTATCCTTAGCTATAACAAACTCACTGCCCCCCACCCCCCCTCGCTCTCTCTCC
Fram_PPT2_3k      TC----------------------------------------------------------
                  **

Fson_PPT2_3k      CCTGATGATCCATAGCCTCCCTCTC--TCCTTTCTGTCAGCGCCCATAGAGAGA----CT
Ftri_PPT2_3k      TCAAATGTTTCATAAATTGTTTCAACTTTACTCT-----------------AAAATATTT
Frob_PPT2_3k      CTTGATGATCCATAGCCTCTCTCTCCTTGCTTCATGTCAGCGCCACTAGAGCGACTGCCT
Fram_PPT2_3k      ------------------------------------------------------------


Fson_PPT2_3k      ACCATTCCTAT---------GGACGATGATTGCGGATGATCAAACAAAAATGCTATATCT
Ftri_PPT2_3k      ATTATTTTGAATTTAAGTTGAAACAACTAATTAAACTTTTGGATTAAAATTGAAATAAAT
Frob_PPT2_3k      GCCATTCGCAA---------GAATGACGATTGTAGATGGTCAAACAAAAATGCTATATCT
Fram_PPT2_3k      ------------------------------------------------------------
```

```
Fson_PPT2_3k      TCCTCAAAATAAGAAGAAAATTATACTCCCACGCATAAGTTAAAGGGCCCTAACGAGGTT
Ftri_PPT2_3k      TTTAAAACTTTGAAGATAAATT-----TATATATATAATCT-----------------TT
Frob_PPT2_3k      TCCTC----CTAAAAATAAATCATACACCCACTCATAAGTTAAAAAGCCCTAATGAGGTT
Fram_PPT2_3k      ----------------------------------------------------------TT
                                                                            **

Fson_PPT2_3k      CAAATTAATGTTATTGTGTTCTTTATTATATTTTAAATATTTTTTTCATTTTCTTATGAT
Ftri_PPT2_3k      TAGATAAAAATGATTA---TTTTTATCAAAACTCAAGGAG-----TCCTTTTCTTATTAT
Frob_PPT2_3k      TAAATTAATGTTATTATG-TCTTTATTAAAATTGAATAAT--TTTTTCTTTTCTTATTAT
Fram_PPT2_3k      TATAT--------------------TTA-AATCTAAATATTTTTTTCCTTTTCTTATTAT
                   * **                    * * *    **  *      *  ********* **

Fson_PPT2_3k      GTATTGCATTAGGC--TTTTTTACAACCCATA-------ACTCACATTCTATTAAATAAT
Ftri_PPT2_3k      GTATTGTATTAGGC-TTTTTTTACAACCAATG-------ACTTACACGCCATTGAATAA-
Frob_PPT2_3k      GTATTGCATTAGTTTTTTTTTTACAACCGCTACTCACACAGTCACACGTCATTGAATAA-
Fram_PPT2_3k      GTATTGTATTAGGCTTTTTTTTACAACCAGTA-------ACTCACACGCCATTGAATAA-
                  ****** *****    ************  *        * * ***    *** *****

Fson_PPT2_3k      GATTGATTTTAACTGTATACATTTGTTTACGATAAGA-----------------------
Ftri_PPT2_3k      ---TGATTCTAACTGTACATATTTATTTACGGTAGGA-----------------------
Frob_PPT2_3k      ---TGATTTTAAGTGTACACATTTATATACGATAAGACTTGAACCTTCAATCATAAAACA
Fram_PPT2_3k      ---TGATTCTAACTCTACACATTTATTTATGATAAGA-----------------------
                     ***** *** * ** * **** * ** * ** **

Fson_PPT2_3k      ------------------------------------------------------------
Ftri_PPT2_3k      ------------------------------------------------------------
Frob_PPT2_3k      AATTTGTAACAAATTTTATGCTACAAGATAACATACCACTTGCTTAAAGAAATGACGAAT
Fram_PPT2_3k      ------------------------------------------------------------


Fson_PPT2_3k      ------------------------------------------------------------
Ftri_PPT2_3k      ------------------------------------------------------------
Frob_PPT2_3k      TAATATCTATTCTGTTAATTTTGACTTATAACCCATTAAATTATATTACCATTAAACGCA
Fram_PPT2_3k      ------------------------------------------------------------


Fson_PPT2_3k      ------------------------------------------------------------
Ftri_PPT2_3k      ------------------------------------------------------------
Frob_PPT2_3k      TTTTACGTGATTGTAATTATATGTAAAGAGTTATAATTTTGTCATGGCTGTATGATGTAA
Fram_PPT2_3k      ------------------------------------------------------------


Fson_PPT2_3k      ------------------------------------------------------------
Ftri_PPT2_3k      ------------------------------------------------------------
Frob_PPT2_3k      TATATGCTAGGTGTTAATTCTCGACACACGTTTGGGTCGCTTGGCATAACCCGTTTATTA
Fram_PPT2_3k      ------------------------------------------------------------


Fson_PPT2_3k      ------------------------------------------------------------
Ftri_PPT2_3k      ------------------------------------------------------------
Frob_PPT2_3k      CATAGATAAACTCAAAAATGATGTGTTTAATATATTAATTAACCAAAAAAACAGTGTGAC
Fram_PPT2_3k      ------------------------------------------------------------


Fson_PPT2_3k      ------------------------------------------------------------
Ftri_PPT2_3k      ------------------------------------------------------------
Frob_PPT2_3k      TCGTTAACTCGATCATTTTATTTAAAATTCTAAAAAAATACATTTCTAGATTTTAGATGT
Fram_PPT2_3k      ------------------------------------------------------------


Fson_PPT2_3k      ------------------------------------------------------------
Ftri_PPT2_3k      ------------------------------------------------------------
Frob_PPT2_3k      GGTTATCTAAAAGTTGCTAACATTTAAAGTTTAATGTCTTGCTTTAAACGAAAATAAAGG
Fram_PPT2_3k      ------------------------------------------------------------


Fson_PPT2_3k      ------------------------------------------------------------
Ftri_PPT2_3k      ------------------------------------------------------------
Frob_PPT2_3k      AGTGCAGCCTACAGTGCCCATGTAACGGACAATTGAGCTGCTCACACAGCCTCGCTTTTT
Fram_PPT2_3k      ------------------------------------------------------------


Fson_PPT2_3k      ------------------------------------------------------------
Ftri_PPT2_3k      ------------------------------------------------------------
```

```
Frob_PPT2_3k      GCTCATTTTTGAGCAATTTTATCAATATAGCTAAAGTTTAATGCTTTTTGATTGTGAATT
Fram_PPT2_3k      ------------------------------------------------------------


Fson_PPT2_3k      ------------------------------------------------------------
Ftri_PPT2_3k      ------------------------------------------------------------
Frob_PPT2_3k      TTTGAAACGACGCGAACCTGCTAGCGCGACAGATCGCAGGTTTGGGTTTAGTGAAAACGG
Fram_PPT2_3k      ------------------------------------------------------------


Fson_PPT2_3k      -----------TTTTGAATCGATACCGTGAACCCAATTAACTAAACAGGTTGTGTTCGTA
Ftri_PPT2_3k      -----------TTTTGAATCGAACCTCTGAACCCATTTAACTAAATGGGTTGTGTTCATA
Frob_PPT2_3k      ATCCTAGTAAGTTTCGAACCGAGCCTGTGAACCCATTTAACAAAATGGATTGTGTTCATA
Fram_PPT2_3k      -----------TTTTGAATCGAGCCCGTGAACCCATTTAACTAAACGGGTTGTGTTCATA
                             *** *** ***  *  ******** ***** ***  * ******** **

Fson_PPT2_3k      TCAACCAGACATGATTCATAGGTTGACGTTTTATAACACATTTACTTTTTATT----TTT
Ftri_PPT2_3k      TCAACCCAGCCGAATTTGCAGGTTGACATGTT-TAACACATTTAGTTTTTA------TTT
Frob_PPT2_3k      TCAATCCGACGGGATTCGTAGGTTGACGTGTT-TAACACATTTAGTTTTTATTTTTATTT
Fram_PPT2_3k      TCAACCAGACCGGATTTACAGGTTGACATGTT-TAACATATTTAGTTT---------TTT
                  **** *   *   ***   ******** * ** ***** ***** ***         ***

Fson_PPT2_3k      TTAAATATGTTTTATGTCATAAATTAAATTGGTTAGGTGTATTATTCTAAAATTTTAAAC
Ftri_PPT2_3k      TTTAATACACTTTGTGTCATAAATAAAATTGGTTAGGTGTGTTATTCTAAAC-TTTAAAC
Frob_PPT2_3k      TTAAATATGTTTTATGTCATAAATTAAATTGGTTAGGTGTATTGTTCTAAAA-TTTAAAC
Fram_PPT2_3k      TTTAACACGTTTTGTGTCATAAATTAAATTGGTTAGGTGTGTTATTCTAAAC-TTTAAAC
                  ** ** *   *** ********** *************** ** *******  *******

Fson_PPT2_3k      TTAATAGAGAAATTGACATAAGATTTTGTAG--TT--AAAATAATTATTTTATATAAGAC
Ftri_PPT2_3k      TTAATAGAGTAATTAACATAAAATTTTGTAATTTTAAAAAATAACTACTT-ATATAAAAT
Frob_PPT2_3k      TTAATAGAGAAATTGACATAAAATTTTGTAA--TT--AAAATAATTCTTTCGTATAAAAC
Fram_PPT2_3k      TTAATAGAGAAATTAACATAAGATTTTGTAA--TTAAAAAATAATTATTT-ATATAAGAT
                  ********* **** ****** ********   **  ******* *  **  ***** *

Fson_PPT2_3k      TGTAATTTTTGTAATTATAAAGTATTAATAATGTGGAATAAT------AAATTAAAATTT
Ftri_PPT2_3k      TATAATTATTGTAATTATAAAGTATTAA---TATGTAAAAAAATATAAAAAATAAAAAAT
Frob_PPT2_3k      ---------TGTAATTATAAAGTATTAATA-TATGGGAAAAA--TTAAAAATTAAAAATT
Fram_PPT2_3k      TATAATTATTGTAATTATAAATTATTAA-ATTGTGGAAAAAAATATAAAAACTAAAAATT
                           ************ ******   * **  * **       *** *****  *

Fson_PPT2_3k      TTGAGTTAAACAACTCATTTTCATGTTAACTTGTGA----ATTTTTATGTTGTGTTCAAG
Ftri_PPT2_3k      TCAAGTTAAA-GGGTCATGTTCATGTTAACTTGTGA----ATTTTTATGGTGTGTTCAGG
Frob_PPT2_3k      TCAAGTTAAACGGGTCATGTTCGTGTTAACTTATTATTATTTTTTTATGTTGTGTTCAGG
Fram_PPT2_3k      TCGAGTTAGACGGGTCATCTTCGTGTTAACTTGTGA----ATTTTAAAGTTGTATCCAGG
                  *  ***** *    **** *** ********* * *     **** * * *** * ** *

Fson_PPT2_3k      TTCATATGTCTAATACGTTTTTCACGCTCAGGTTGTGTCCGAATTCGTGTCGAGTTAGAC
Ftri_PPT2_3k      TTCATACG-CTAACACGTTTTTCATGTCCAGGTCTTGTCTTGATTTGTGTCAGGTTAAAA
Frob_PPT2_3k      TTCATAAGCCTAA---------------CTAGGTCGTGTCTGGATTCGTGTCGGGTTAGAC
Fram_PPT2_3k      TTCATACG-CTTACACATTTTCCATGTCCAGGTCGTGTCCGGATTCGTGTCAGGTTAGAC
                  ****** * ** *                ****  ****   *** *****  **** *

Fson_PPT2_3k      CCGTCAATCCACAAACACTATCCATTTAGTGTCCGTTTAAAATGAGTTTCCTCATCATTT
Ftri_PPT2_3k      TCGTCAGTCCACAAACACCATTTGTTTTG---CTAGTTAAAATGAGTTTCCTCTTCATTT
Frob_PPT2_3k      CCGTTAGTCCACAAACACTATCCGTTTAGTGCCCATTTAAAATGAGTTTCCTCTTTATTT
Fram_PPT2_3k      ACGTCAGTCCACAAACACCATCCATCTAGTG-CCGGTTAAAATGAGTTTCCACTTTATTT
                   *** * *********** **   * * *   *   *************** * * ****

Fson_PPT2_3k      TTTTCAGTTTTATATATCACTCTTAAATACT-------TATTTTCACCTTTCCATTCTTC
Ftri_PPT2_3k      GTAGCAGTCTTATCTATCACTCTTAACTATTTAATATATATTTTCACCGTTCCACTCTAC
Frob_PPT2_3k      TTAACAGTTTTATCTATCACTCTTAACTATT-------TATTTTCATCTTTCCATTCTAC
Fram_PPT2_3k      -TAGCAGTCTTATCTATCACTCTTAACTATT-------TATTTTCACCATTCCATTCTAC
                   *  **** **** ************ ** *       ******** * ***** *** *

Fson_PPT2_3k      AATCTTTTCTATCCGGTCCTTTAATATCAAGCTCCAT-----------------------
Ftri_PPT2_3k      AATCTTGTCTATCCGGTCCTCTAATATCAAGCTATATATATTATATATATATATATATAA
Frob_PPT2_3k      AATCTTTTCTATCCTGACCCCTAATATCAAGCTCCATATAT----------------AAA
Fram_PPT2_3k      AATCTTTTCTATCCGGTCCTCTAATATCAAGCTATATATAT----------------AAA
                  ****** ******* * **  ************  **

Fson_PPT2_3k      ---CAAACTTTCTAATTGAGATTTGAG-CTTGAGTCATTGGTAATTTAAATTGTTACCAA
Ftri_PPT2_3k      ACCCAAACTTTTTAATTGAGATTAAAAGCTTGAGTCGTTGGTTATGAAAATTGTTACCAA
Frob_PPT2_3k      ACCCAAACTTTTTAATTAAGACTTAAG-TTTGAATCATTGGTTATTTAAACTGTAACCAA
Fram_PPT2_3k      ACCCAAACTTT-TAATTGAGTTTTAAGCTTTGAGTCATTGGTTATTTAACTTGTTACCAA
                     ******** ***** **  *  *   **** ** ***** **  **  *** *****
```

```
Fson_PPT2_3k      ACCATCAACTATGACTCATATCCACAATAAACTTTAAAAAGGGCAGCCAAAACACAAATC
Ftri_PPT2_3k      ACCATCA----------------ACAATAAACTTCAAAAAAGACAAC--AAGCACAAATC
Frob_PPT2_3k      GCCATCAACTAATACTCATATCCACAATAAACTTTAAAAAGAGCAACCAAAACACAAATC
Fram_PPT2_3k      ACCATCAACTATTACTCATATCCACAATAAACTTTAAAAAGGGCAACCAAAACAAAAAAC
                   ******                ***********  *****   ** *  ** ** *** *

Fson_PPT2_3k      -AGTGAATCATACACAACA-TGAGTTTATATATACTCTTATTAGA---------GTTGAT
Ftri_PPT2_3k      AAAAGAATCATACACAACA-CGAGTTTAATCAAA-------------------GTTGAT
Frob_PPT2_3k      -AATGAATCATACACAACA-TGAGTTTAAATGTACCCTAAATGTACCCCTGTCGGTTGAT
Fram_PPT2_3k      -AAAGAATCATACACAACATTGAATTTTA-------------------------------
                   *  ***************  ** ***

Fson_PPT2_3k      ACCTTTAACACAAATAAACATAATG-----------------------------------
Ftri_PPT2_3k      ATATTAAACACAAATAAGCATAATG-----------------------------------
Frob_PPT2_3k      ATTTTTAACACAAATAAACATAATGCCCATGTTTGGGATTTTTCATTTTATCTAACAGTA
Fram_PPT2_3k      ATCTTTAACACAAATAAACATAATA-----------------------------------
                  *  ** *********** ******

Fson_PPT2_3k      ------------------------------------------------------------
Ftri_PPT2_3k      ------------------------------------------------------------
Frob_PPT2_3k      ACTTTTCAAAAGTTACTTCTATTAACTATTCAAATTTAACTTATTAACTTTTTAAAATCA
Fram_PPT2_3k      ------------------------------------------------------------


Fson_PPT2_3k      ------------------------------------------------------------
Ftri_PPT2_3k      ------------------------------------------------------------
Frob_PPT2_3k      ACTTTTTAAAATTTTGTCAAACATAATTATTAACTTGTTAACTTTTTAAAAGTTAATAAG
Fram_PPT2_3k      ------------------------------------------------------------


Fson_PPT2_3k      -------------------------------------------ATAATCGTCGGGTTCAC
Ftri_PPT2_3k      -------------------------------------------ATGATCGTTAAGTTCAC
Frob_PPT2_3k      TCAAAAGTTACTCAAAAAATTGGTTTGCCAAACATGACTAATAATCATCCTTGTATTCAC
Fram_PPT2_3k      -------------------------------------------ATCATCGTCGGGTTCAC
                                                             ** *** *    *****

Fson_PPT2_3k      CTGTTCAACCC-------------------------------------------------
Ftri_PPT2_3k      CTGTTAAACCCG--------------TAACAATTAACACCCT---AACATTTGAAGGATT
Frob_PPT2_3k      CAGTTCAACCCTTCGACCATAACAATTAACAATTAACACCCT---AACATAAGAAGAATT
Fram_PPT2_3k      CTGTTCAACCCGTCTACCG-------TAACAATTATCACCCTATAAACATTTGAAGGATT
                  * *** *****

Fson_PPT2_3k      ATTGGAGGACAATTTTTTCTGTCTCATACCAAGGAGTCCAAATGTGGA-AACTAAAGAAA
Ftri_PPT2_3k      GTTGGAGGACAA-TTTTTATGTCTCCTACCAAAGAA-CCAAATGTGGA-AACTACAGAAA
Frob_PPT2_3k      GATGGAGGATAATTTTTTCTCTCTCATACCAAAGAATCCAAATATGGATAAGTAAAGAGA
Fram_PPT2_3k      GTTGGAGGACAATTTTTTCTGTCTCATACCAAAGAATCCAAATGTGGA-AACTAAAGAAA
                    ******* ** ***** * **** ****** **  ****** **** ** ** *** *

Fson_PPT2_3k      ACAAAACATAAAACACTTGTCACGTTTTGTTCTGAAAAAACATTTATGGTGACTAAATGA
Ftri_PPT2_3k      A-AAAACATAAAACACTAGTCACGTTTTGTTCTGAAAACACATTAATGGGGACCAAATGA
Frob_PPT2_3k      A-AAAACAT-AAACACTAGTCACGTTTTGTTCTGGAAAAACATTTATGGTGACTAAATGA
Fram_PPT2_3k      A-AAAACACAAAACACTAGTCACGTTTTGTTCTGAAAAAACATTTATGGGGACTAAATGA
                  * ******  ******* **************** *** ***** **** *** ******

Fson_PPT2_3k      CAGCCATAAATGTTTGTAGTACACTTACATTTACAGTCA---------------------
Ftri_PPT2_3k      CAGCCATAAATGTATGTGCTACACTTACATTCACAGTCACTCATGATTTATATATGTTTT
Frob_PPT2_3k      CAGCCATA----------------------------------------------------
Fram_PPT2_3k      CAGCCATAAATGTATGTACTACACTTACATTCACATTCATTCATGATTTATATA--TTTT
                  ********

Fson_PPT2_3k      ------------------------------------------------------------
Ftri_PPT2_3k      GGATTTATACTCCATGCAAACTATGCCACAATAAAGATCTTCCTTTCTAGGCCACATATA
Frob_PPT2_3k      ------------------------------------------------------------
Fram_PPT2_3k      GGATTTATACTTCATGCAAACTATGCCACAATAAAGATCTTCCTCTCTAGGCCACATATA


Fson_PPT2_3k      ---------------------------------------
Ftri_PPT2_3k      CACAAGCAAAATCTTCTTTGATCTTCCACAAGCCATCAA
Frob_PPT2_3k      ---------------------------------------
Fram_PPT2_3k      CACAAGCAAAATCTCCTTTGATCTTCCACAAGCCATCA-
```
